# Population Genetic Structure and Potential Incursion Pathways of the Bluetongue Virus Vector *Culicoides brevitarsis* (Diptera: Ceratopogonidae) in Australia

**DOI:** 10.1371/journal.pone.0146699

**Published:** 2016-01-15

**Authors:** W. T. Tay, P. J. Kerr, L. S. Jermiin

**Affiliations:** 1 CSIRO, Black Mountain Laboratories, Canberra, ACT, 2601, Australia; 2 School of Biological Sciences, The University of Sydney, Sydney, 2006, Australia; National Cheng-Kung University, TAIWAN

## Abstract

*Culicoides brevitarsis* is a vector of the bluetongue virus (BTV), which infects sheep and cattle. It is an invasive species in Australia with an assumed Asian/South East Asian origin. Using one mitochondrial marker (i.e., part of the cytochrome oxidase subunit I gene) and six nuclear markers, we inferred population genetic structure and possible incursion pathways for Australian *C*. *brevitarsis*. Nine mitochondrial haplotypes, with low nucleotide sequence diversity (0.0–0.7%) among these, were identified in a sample of 70 individuals from seven sites. Both sets of markers revealed a homogeneous population structure, albeit with evidence of isolation by distance and two genetically distinct clusters distributed along a north-to-south cline. No evidence of a cryptic species complex was found. The geographical distribution of the mitochondrial haplotypes is consistent with at least two incursion pathways into Australia since the arrival of suitable livestock hosts. By contrast, 15 mitochondrial haplotypes, with up to four times greater nucleotide sequence diversity (0.0–2.9%) among these, were identified in a sample of 16 individuals of the endemic *C*. *marksi* (sampled from a site in South Australia and another in New South Wales). A phylogenetic tree inferred using the mitochondrial marker revealed that the Australian and Japanese samples of *C*. *brevitarsis* are as evolutionarily different from one another as some of the other Australian species (e.g., *C*. *marksi*, *C*. *henryi*, *C*. *pallidothorax*) are. The phylogenetic tree placed four of the species endemic to Australia (*C*. *pallidothorax*, *C*. *bundyensis*, *C*. *marksi*, *C*. *henryi*) in a clade, with a fifth such species (*C*. *bunrooensis*) sharing a common ancestor with that clade and a clade comprising two Japanese species (*C*. *verbosus*, *C*. *kibunensis*).

## Introduction

Accurate delineation of a vector species’ status and its population and evolutionary genetics are fundamental to biosecurity and mitigation of the emergence of exotic diseases. Cryptic species (i.e., morphologically indistinguishable but genetically different species) are widespread among eukaryotes and prokaryotes, including all major orders of insects (e.g., Lepidoptera [1], Diptera [2], and Hemiptera [3]). The detection of cryptic species in the dipteran *Culicoides variipennis* species complex and the subgenus *Culicoides* [4] had major implications for our understanding of vector competence for Bluetongue virus (BTV) [5].

Delineation of cryptic species and rapid identification of morphologically similar species are now feasible using genetic markers such as the nuclear ribosomal RNA (rRNA) genes (e.g., [6–9], but see [10] for caution relating to concerted evolution and the use of rRNA as a genetic marker) and the mitochondrial cytochrome oxidase subunit I (COI) [4, 11–14] and cytochrome *b* (Cyt *b*) [15] genes. Important population genetics features, such as migration, gene flow, and mating behaviour (e.g., [16–18]), all benefit from the use of nuclear DNA (nuDNA) and mitochondrial DNA (mtDNA) (e.g., [19–22]), and the use of mtDNA to delineate, and infer the phylogeny of, closely related species has been augmented by using nuDNA markers and morphological data (e.g., [14, 20, 23]).

Globally, there are currently 1401 recognized species (1355 extant species, 46 fossil species) and 31 subgenera of *Culicoides* [24–26], and many of them are thought to be cryptic species groups. Traditionally, species identification of *Culicoides* has relied on external (e.g., head or wing patterns [12, 14]) and internal (e.g., genitalia [27, 28]) traits. Using external traits to identify species can lead to confusion and hamper the identification and confirmation of virus vector status and competency (e.g., *C*. *brevitarsis*, *C*. *actoni*, and *C*. *suzukii* in the *Avaritia* group [29], and the *C*. *variipennis* species complex [5]). By contrast, different molecular markers have been used successfully to identify and delineate species of *Culicoides* from Africa, Europe, and the USA (e.g., [9, 12, 30–38]), but so far not from Australia.

In Australia, *Culicoides* consists of introduced and endemic species [39]. Dyce *et al*. [27] divided the Australasian species of *Culicoides* (145 described, 120 un-described) into 23 subgenera, with the subgenus *Avaritia* divided into five complexes that contain four introduced BTV vector species (i.e., *C*. *actoni* (*Actoni* complex), *C*. *wadai* (*Boophagus* complex), *C*. *brevitarsis* (*Imicola* complex), and *C*. *fulvus* (*Orientalis* complex)) of Asian or South-east Asian origins [39, 40]. Oviposition sites are highly specialised in *Culicoides* species [41, 42]. Oviposition by *C*. *brevitarsis* appears to be limited to cattle dung [42–44], with adults feeding on both cattle and sheep [45, 46]. Hence, it seems likely that *C*. *brevitarsis* only was capable of founding breeding populations in Australia after the introduction of domesticated cattle by European settlers.

*Culicoides wadai*, *C*. *actoni* and *C*. *fulvus*, while competent vectors of BTV, have only been found in northern Australia [47]. BTV is asymptomatic in cattle across the ranges of *C*. *brevitarsis*, *C*. *wadai*, *C*. *fulvus*, and *C*. *actoni* in Australia. By contrast, sheep are highly susceptible to BTV but are farmed predominantly in the southern parts of Australia, where they overlap geographically with *C*. *brevitarsis* in its southern-most range of New South Wales (NSW). Therefore, a transfer of BTV into sheep could occur in these parts of Australia, posing a risk to this region’s livestock industry.

Given that *C*. *brevitarsis* is a competent vector of BTV and regarded as the most likely vector of BTV between Australian cattle and sheep, it is important that a better understanding of this species be developed. In particular, information on its population genetic structure is needed, but we also need to determine whether there is evidence of it being a cryptic species complex, whose members could have different levels of vector competency for BTV. Finally, because *C*. *brevitarsis* may have been recently introduced to Australia, comparing Southeast Asian and Australian samples of *Culicoides* might reveal the evolutionary relationship among these samples, and point to the likely incursion pathways. Such information could become critical for a successful Australian response to a BTV epizootic event like that in Europe (reviewed in [48–50]). The *C*. *variipennis* species complex illustrates the importance of such information: different subspecies in this complex have different levels of susceptibility to BTV infection and different virus competency rates [5].

Using molecular data obtained from Australian samples of *C*. *brevitarsis* and *C*. *marksi* (included for comparative purposes) as well as published sources (GenBank), this study aims to address some of the issues mentioned above. In particular, it aims to determine whether Australian samples of *C*. *brevitarsis*: (i) are genetically diverse; (ii) form a hitherto unknown cryptic species complex; and (iii) differ genetically from their Southeast Asian counterparts.

## Materials and Methods

### Sampling method, sampling sites and species identification

**Fig 1:**
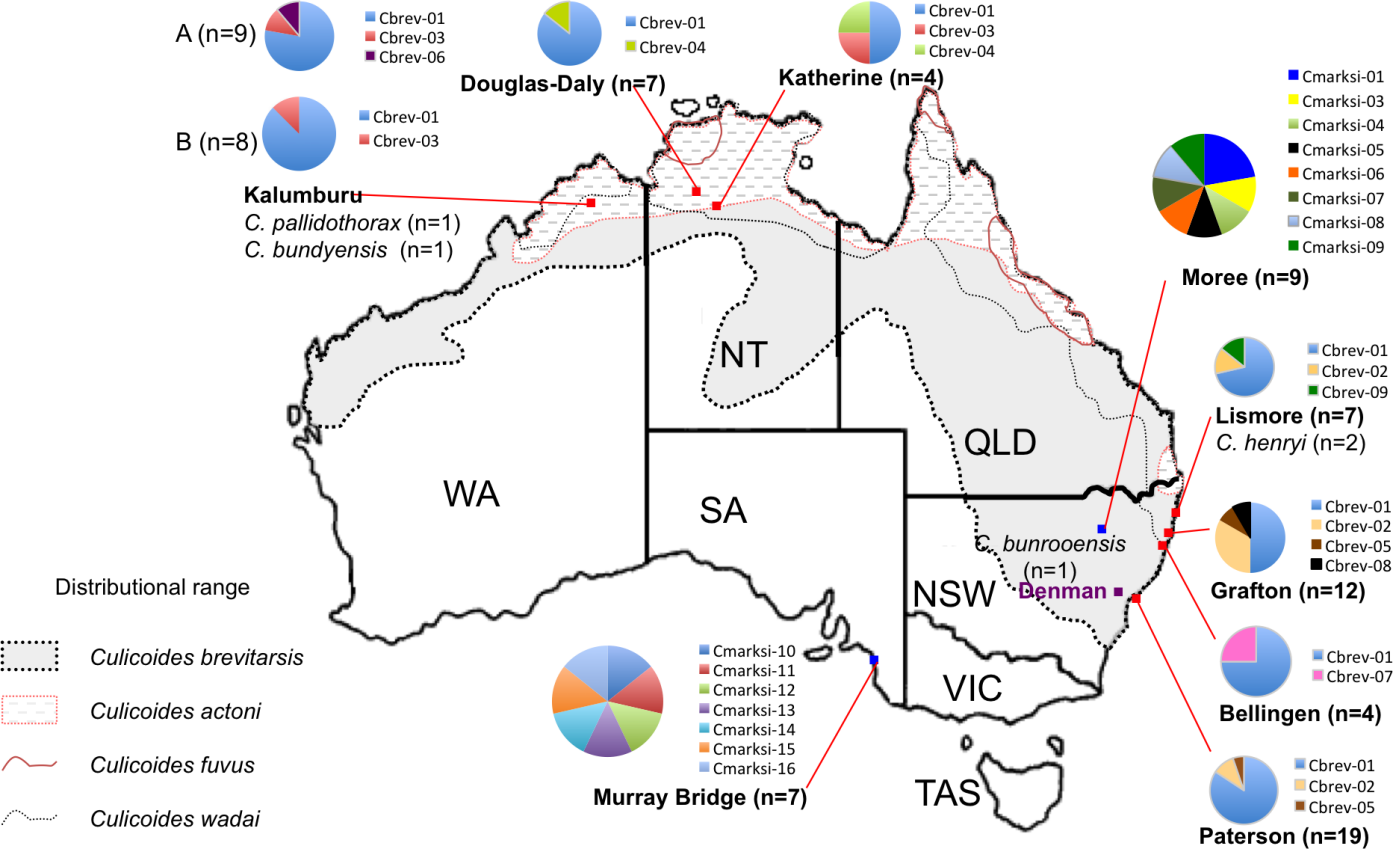
*Culicoides* sampling sites. Geographical range of the four BTV competent *Culicoides* vector species in Australia (modified from [40]) and locations from which samples of *Culicoides* were collected for this study: *C*. *brevitarsis* (from Kalumburu, Western Australia (WA); Douglas-Daly and Katherine, Northern Territory (NT); Lismore, Grafton, Bellingen, and Paterson, New South Wales (NSW)); *C*. *marksi* (from Moree, NSW; Murray Bridge, South Australia (SA)); *C*. *pallidothorax* and *C*. *bundyensis* (also from Kalumburu, WA); *C*. *henryi* (from Lismore, NSW); and *C*. *bunrooensis* (from Denman, NSW). *Culicoides brevitarsis* and *C*. *marksi* mtDNA haplotypes (respectively labelled ‘Cbrev’ and ‘Cmarksi’) identified from all sampling sites are also indicated. *C*. *brevitarsis* and *C*. *marksi* individuals analysed per site are indicated by ‘n’. Kalumburu sampling dates are 29-Dec-08 ‘A’; 25-Jun-09 ‘B’.

*Culicoides brevitarsis* and *C*. *marksi* were collected using light traps at the National Arbovirus Monitoring Program’s (NAMP) monitoring sites in the New South Wales (NSW), Northern Territory (NT), Western Australia (WA), and South Australia (SA) (Table 1). Australian state, territory, and federal government officers are responsible for monitoring the NAMP. Samples obtained for this study were provided in-kind by the relevant government departments via the Officer in Charge, so no specific written permissions were required. The Officer in Charge at the various government departments (e.g., NSW Department of Primary Industry; Department of Agriculture, Forestry and Fishery; Department of Agriculture; and Northern Australia Quarantine Strategy) identified the name of each specimen using the keys by Dyce *et al*. [27]. *Culicoides brevitarsis* were sampled from: Kalumburu in WA (‘A’ 29-Dec-08; ‘B’ 25-Jun-09), Douglas-Daly (28-Nov-08) and Katherine (28-Feb-08) in NT, and Lismore (14-Jan-08), Grafton (14-Dec-07), Bellingen (14-Jan-08), and Paterson (6-Feb-08) in NSW. Two populations of *C*. *marksi* (n = 16), a species endemic to Australia, were also sampled from Murray Bridge in SA (20-Jan-09) and Moree in NSW (10-Feb-08) to enable comparison of nucleotide sequence diversity at the population level (Fig 1). Four other Australian endemic species (i.e., *C*. *henryi* (Lismore; 13-Nov-07), *C*. *bundyensis* (Kalumburu; 19-Dec-08), *C*, *pallidothorax* (Kalumburu; 29-Dec-08), and *C*. *bunrooensis* (Denman, NSW; 13-Mar-08)) were included in the study to allow direct comparison of estimates of inter-species nucleotide sequence diversity and contribute to our understanding of phylogenetic relationships between Australian and Asian/South East Asian *Culicoides* species. Individuals of *C*. *pallidothorax*, *C*. *henryi*, *C*. *bundyensis*, and *C*. *bunrooensis* from this study have been deposited in the Australian National Insect Collection’s database (ANIC Database: No. 29035987, 2903588, 29035989, 29035990, respectively) and thus are available to future research efforts, such as characterisation of the species based on morphological characters (e.g., wing patterns). Wing patterns and partial mtDNA COI sequence [GenBank: KJ162974] of a *C*. *brevitarsis* individual from Grafton (also collected by H. McKenzie on the same date) have been presented [51].

**Table 1:** *Culicoides* samples used in this study including known virus vectored and taxonomic status.

**Location**	***Culicoides* spp.**	**N**	**Subgenus**^**1**^**/Sp. Gp.**^**2**^**/Complex**^**3**^	**Date collected**	**Viruses vectored**
Kalumburu, WA	*pallidothorax*	1	unplaced^1^^a^ / *Victoriae*^1^^a^	29/12/08	?
	*bundyensis*	1	unplaced^1^^a^ / *Victoriae*^1^^a^	19/12/08	?
	*brevitarsis (A)*	9	*Avaritia*^1^^a^/*Imicola*^3^^a^	29/12/08	BTV/BEF/EHDV/AV
	*brevitarsis (B)*	8	*Avaritia*^1^^a^/*Imicola*^3^^a^	25/06/09	BTV/BEF/EHDV/AV
Douglas-Daly, NT	*brevitarsis*	7	*Avaritia*^1^^a^/*Imicola*^3^^a^	28/11/08	BTV/BEF/EHDV/AV
Katherine, NT	*brevitarsis*	4	*Avaritia*^1^^a^/*Imicola*^3^^a^	28/2/08	BTV/BEF/EHDV/AV
Lismore, NSW	*henryi*	2	unplaced^1^^a^ / *Victoriae*^2^^a^	13/11/07	?
	*brevitarsis*	7	*Avaritia*^1^^a^/*Imicola*^3^^a^	13/11/07	BTV/BEF/EHDV/AV
Grafton, NSW	*brevitarsis*	12	*Avaritia*^1^^a^/*Imicola*^3^^a^	14/12/07	BTV/BEF/EHDV/AV
Bellingen, NSW	*brevitarsis*	4	*Avaritia*^1^^a^/*Imicola*^3^^a^	14/01/08	BTV/BEF/EHDV/AV
Paterson, NSW	*brevitarsis*	19	*Avaritia*^1^^a^/*Imicola*^3^^a^	06/02/08	BTV/BEF/EHDV/AV
Denman, NSW	*bunrooensis*	1	unplaced^1^^a^ / Clavipalpis^2^^a^	13/03/08	?
Moree, NSW	*marksi*	9	Marksomyia^1^^a^/*Marksi*^2^^a^	10/2/08	BEF^†^
Murray Bridge, NSW	*marksi*	7	Marksomyia^1^^a^/*Marksi*^2^^a^	20/1/09	BEF^†^
**GenBank Accession Number**	***Culicoides spp*.**	**N**		**Nucleotide positions (start.end)**	
AB360994.1, AB360995.1	*brevitarsis*	2	*Avaritia*^1^^a^/*Imicola*^3^^a^	326.872 (123. . .957)^e^	BTV/BEF/EHDV/AV
KJ162974.1	*brevitarsis*	1	*Avaritia*^1^^a^/*Imicola*^3^^a^	365.704	BTV/BEF/EHDV/AV
KJ162966.1, KJ162972.1, KJ162973.1	*brevitarsis*	3	*Avaritia*^1^^a^/*Imicola*^3^^a^	324.646	BTV/BEF/EHDV/AV
KJ162971.1	*brevitarsis*	1	*Avaritia*^1^^a^/*Imicola*^3^^a^	324.646	BTV/BEF/EHDV/AV
KJ162970.1, KJ162969.1, KJ162967.1	*brevitarsis*	3	*Avaritia*^1^^a^/*Imicola*^3^^a^	324.658	BTV/BEF/EHDV/AV
KJ162975.1	*brevitarsis*	1	*Avaritia*^1^^a^/*Imicola*^3^^a^	324.646	BTV/BEF/EHDV/AV
KJ162968.1	*brevitarsis*	1	*Avaritia*^1^^a^/*Imicola*^3^^a^	324.646	BTV/BEF/EHDV/AV
AB361000.1	*dubius*	1	*Culicoides*^1^^b^	578.1,124	?
AB360979.1	*oxystoma*	8	*Remmia*^1^^a^ / (*Oecacta*^1^^c^)?	4,679.5,225	BTV
AB360978.1, AB360980.1, AB360981.1, AB360982.1, AB360983.1, AB360984.1, AB360985.1				326.872	BTV
AB361001.1, AB361002.1	*japonicus*	2	*Beltranmyia*^1^^b^^,^^e^	326.872	?
AB646612.1, AB646613.1	*pictimargo*	2	*Oecacta*^1^^d^	326.872	?
AB646615.1	*verbosus*	1	*Oecacta*^1^^c^	326.872	?
AB360973.1, AB360974.1, AB360975.1, AB360972.1	*arakawae*	5	*Meijerehelea*^1^^a^^,^^d^/ (*Beltranmyia*^1^^b^)?	541.1,087	BTV
AB361004.1				1,994.2,540	
AB364649.1, AB364650.1	*matsuzawai*	2	*Trithecoides*^1^^b^	326.872	?
AB646611.1	*kibunensis*	2	*Oecacta*^1^^c^	326.872	?
AB646610.1				1,376.1,922	
AB360988.1, AB360989.1	*punctatus*	2	*Culicoides*^b^	326.872	?
AB361005.1	*wadai*	3	*Avaritia*^1^^a^ / *Boophagus*^3^^a^	1,885.2,431	BTV
AB360996.1, AB360997.1				578.1,124	
AB360999.1	*nipponensis*	1	*Culicoides*^b^	326.875	?
AB361003.1	*peregrinus*	1	*Hoffmania*^1^^a^/ (*Culicoides*^1^^b^)?	326.875	BTV
AB360990.1, AB360991.1	*jacobsoni*	2	*Avaritia*^1^^a^/*Jacobsoni*^3^^a^	326.872	?
AB361006.1	*cylindratus*	2	*Culicoides*^1^^b^ / (*Hoffmania*^1^^d^)?	323.869	?
AB361007.1				5,692.6,238	
AB360998.1	*brevipalpis*	1	*Avaritia*^1^^a^/*Imicola*^3^^a^	326.872	BTV
AB364651.1	*humeralis*	3	*Trithecoides*^1^^b^	1,809.2,355	?
AB360992.1, AB360993.1				326.872	
AB360986.1, AB360987.1	*maculatus*	2	*Avaritia*^1^^a^/*Orientalis*^3^	326.872	?
AB360971.1	*actoni*	1	*Avaritia*^1^^a^/*Actoni*^3^	6,015.6,561	BTV
AB360976.1, AB360977.1	*ohmorii*	2	*Hoffmania*^1^^a^ / (*Culicoides*^1^^b^)?	326.872	?

*Culicoides* samples used in this study including known virus vectored and taxonomic status. *Culicoides* species collected from Australian sites (Western Australia (WA); Northern Territory (NT); New South Wales (NSW); South Australia (SA)), as well as from GenBank used in this study. Number of individuals used (N), collection dates, taxonomic classification for subgenus, species group (Sp. Gp.) and complex also indicated. The nucleotide start and end positions for GenBank samples included in the calculation of nucleotide diversities are listed. Viruses vectored are: bluetongue virus (BTV), bovine ephemeral fever (BEF), epizootic haemorrhagic disease virus (EHDV), Akabane virus (AV), unknown (?).

† indicates virus replication detected but transmission status not confirmed.

**Note:** Dyce *et al*. [27]

(a); Encyclopedia Of Life <http://eol.org>

(b); Arthropod Vectors of interest for Animal health database <http://avabase.cirad.fr/>

(c); FLYTREE Assembling the Diptera Tree of Life http://wwx.inhs.illinois.edu/files/9613/9136/7590/CulicoidesSubgenera.pdf

(d). The subgenus status for the following species is uncertain: (i) *C*. *oxystoma* is unclear and has been grouped within the *Remmia* subgenus by Dyce *et al*. [27] or within the subgenus *Oecacta* <http://www.inhs.uiuc.edu/research/FLYTREE/index.html>; (ii) *C*. *peregrinus* and *C*. *ohmorii* are either placed in the subgenera of *Hoffmania* [27] or *Culicoides* <http://avabase.cirad.fr/>; (iii) *C*. *wadai* placed either in the subgenera of *Avaritia* [27] or *Boophagus* (FLYTREE); (iv) *C*. *arakawae* placed either in the subgenera of *Meijerehelea* [27]; FLYTREE) or *Beltranmyia* <http://avabase.cirad.fr/>.

(e) 835bp also used in pairwise sequence divergence estimates between *C*. *brevitarsis* haplotypes from Australia and Japan. *C*. *maculates* (= *C*. *tainanus*)^a^

### DNA extraction, mtDNA PCR markers design, PCR conditions, and DNA sequencing

Genomic DNA (gDNA) was extracted from individual specimens of *Culicoides* using Qiagen’s DNeasy Blood and Tissue Kit (Cat. # 69506) following the recommended protocol. gDNA from individual samples were eluted in 5μl of the elution buffer and stored at -20°C. Forward and reverse primers for PCR amplification of partial mtDNA COI regions (Table 2) were derived from a multiple sequence alignment of mtDNA COI sequences from: *C*. *brevitarsis* [GenBank: AB360994], *C*. *wadai* [GenBank: AB361005], *C*. *dubius* [GenBank: AB361000], and *C*. *oxystoma* [GenBank: AB361002]. PCR amplification of each sample was carried out in a total of 25μL PCR reaction volume that included 2.5μL of gDNA, 0.25mM of both forward and reverse primers, 0.1mM of dNTP’s, 1x PCR buffer (NEB), 1.5mM of MgCl_2_, and 0.025U of Taq DNA Polymerase (NEB). PCR involved an initial 5 minutes template-denaturing step at 95°C, followed by 35 cycles of denaturation at 95°C, primer annealing at 50°C, and template extension at 72°C of 1 minute each. A final template-extension step at 72°C for 5 minutes was included after the 35 PCR cycles, before incubation at 10°C. PCR products (amplicons) were confirmed by gel electrophoresis (1% 1xTAE agarose gel) stained with GelRed (Biotium; cat # 41003) at 85V for 45 minutes.

**Table 2:** Primers for mtDNA COI regions and exon-primed intron-crossing (EPIC) primers for ribosomal protein nuclear genes used to infer population structure of *Culicoides* species.

Primer name	Primer sequence	Temp (°C)	Size (bp)	Fluorescent dye
CO1R1	CCAACWGTAAAYATRTGATGWGCTC	54		
CO1F2A	GCWGTWAATTTYWTWACHACWATTAT	48		
CO1R2	CTATGTTCDGHWGGNGGWARATTTTG	56		
CO1209F	CWATYATAATTGGDGGRTTYGG	51		
CO1265F	GATATRGCWTTYCCKCGWATAAATAA	52		
CO1R1295	AATATCGTCGAGGTATTCCTGA	51		
CO1F272	ATTAGGAGCTCCTGATATAGC	50		
CO1hmbF	GCYATTTTATTAYTWTTATCTTTAC	46		
CO1hmbR	ACATAATGRAARTGWGCTACTAC	50		
RpS2B-f	GGYGTSAARTGCAGCAAGGAAGT		509	FAM
RpS2B-r	TGTGGCCTTKGCRAAGTTACC			
RpS6-f	TAGGYGATGAATGGAARGGCTATGT		234	FAM
RpS6-r	CAATRCAKCCACGAACKGAYTTACG			
RpL27A-f	GAAARAAGACCAGRAAGCTACG		233	TET
RpL27A-r	CGAARTAWCCRGGRTGGTAYTTGTC			
RpS8-f	TGCMACBGGTGGYAAGAAGG		163	TET
RpS8-r	GGYATCHAGACGVAGVGCAC			
Cimi69-f	TCACAAGGAGTCGTTACTTTTGC		232	TET
Cimi69-r	CAAGGATGAATAACAGTAAACAG			
Cimi85-f	CAATAACACTCGCTGACTTGCTG		241	FAM
Cimi85-r	TGCCTATACATGTTAGTAGATAG			

Primer sequences for partial mtDNA COI region, *Culicoides* ribosomal protein (Rp) gene EPIC markers, and microsatellite DNA markers (Cimi; based on *C*. *imicola* sequences [GenBank: DQ008972; GenBank: DQ008973] that contained simple sequence repeat units) used to infer population genetic structure of *Culicoides* species in Australia. Primer annealing temperature (°C) and florescence dye modifications (FAM, TET) added to 5’ end of EPIC-PCR and microsatellite DNA markers are provided. IUPAC nucleotide ambiguity codes used. Approximate expected amplicon size (in bp) for individual nuclear DNA and mtDNA markers are also provided.

**Note:** expected amplicon sizes for: COIF2A/COIR1: 407bp; COIF2A/COIR2: 1,031bp; COIF2A/COIR1295: 839bp; COI209F/COIR1: 673bp; COI209F/COIR2: 1,297; COI209F/COIR1295: 1,105bp; COIF272/COIR1: 630bp; COIF272/COIR2: 1,254bp; COIF272/COIR1295: 1,062bp; COI209F/COIR1295: 980bp; COIhmbF/COIhmbR: 547bp.

Amplicons from individual samples were purified and eluted in 25μL ddH_2_O using Qiagen’s Qiaquick PCR purification kit (Cat. no. 28104). For sequencing of individual samples, 2μL of purified amplicon for each sample was used as template in individual DNA sequencing reactions, which also contained 1.6mM primer, 1.5μL of 5x BigDye sequencing buffer and 1μL of BigDye V3 sequencing reaction mix, made up to a final volume of 10μL. The sequencing profile followed that of Behere *et al*. [16] prior to electrophoresis at John Curtin Medical School of Research, Australian National University, Canberra, Australia.

### Design of exon-primed intron-crossing markers and microsatellite DNA markers

We also designed ribosomal protein (Rp) gene exon-primed intron-crossing (EPIC) markers for *C*. *brevitarsis* based on the methods reported in [52] and [19]. Briefly, six *C*. *sonorensis* Rp mRNA sequence entries in GenBank (RpL27A [GenBank: AAV84242.1], RpS2A [GenBank: AAV84247.1], RpS2B [GenBank: AAV84248.1], RpS4 [GenBank: AAV84250.1], RpS6 [GenBank: AAV84251.1], RpS8 [GenBank: AAV84252.1]) were searched for the presence of introns of between 120bp and 400bp by comparison with the homologs in *Bombyx mori*, and primers anchoring exon boundaries were designed to maximise conserved sequences between *Aedes aegypti*, *Ae*. *albopictus*, *Anopheles gambiae*, and *Culex quinquefasciatus*. EPIC primers were designed using Oligo version 7.17 (Molecular Biology Insights, Inc. Cascade, CO 80809, USA) with criteria that included T_m_ ≥ 60°C, minimal/no primer duplex and hairpin formation, and minimal false priming sites. The resulting primers were examined for PCR amplification efficacies, expected amplicon lengths, and the presence of polymorphism in selected samples of *C*. *brevitarsis* from Lismore, Katherine, Douglas, Kalumburu, and Paterson prior to the population-wide screening and scoring.

Microsatellite DNA markers were designed by searching reported microsatellite loci sequences available in the GenBank. We followed the methods of Tay *et al*. [53] to screen against the potential of incorporating flanking primer-annealing sequences that might be due to Transposable Elements (TEs). Briefly, four microsatellite DNA loci (i.e., Cimi-12 [GenBank: DQ008970], Cimi-66 [GenBank: DQ008971], Cimi-69 [GenBank: DQ008972] and Cimi-85 [GenBank: DQ008973]) were searched for possible target-site duplication sequences, as well as reverse-translated using BLASTX [54] to identify the likely presence of a reverse transcriptase gene, and to search for the presence of target-site duplication sequences that would indicate Class I retrotransposable TE sequence insertions. We note that using BLASTX to identify the presence of reverse transcriptase is only effective against Class I (RNA-associated/‘copy and paste’) TEs but ineffective against Class II (DNA-base/‘cut and paste’) TEs (see [55]).

Conditions used during PCR amplification and expected amplicon sizes for the EPIC and microsatellite DNA markers are provided in Table 2. We used the Fragment Profiler software within the MegaBACE Genetic Profiler suite v1.2 (GE Healthcare Life Sciences, UK) for allele scoring of EPIC and microsatellite DNA loci of biting midges samples in this study. EPIC and microsatellite DNA markers were labelled either with TET or FAM [19], and electrophoresis of PCR amplicons was carried out at the Genetic Analysis Facility, James Cook University (Townsville, Queensland, Australia), as described in Behere *et al*. [19].

### Inter- and intra-species nucleotide diversity and genetic distances in *Culicoides*

Primary sequencing reads were assembled into contigs using Pregap4 and Gap4 from the Staden package [56] and the resulting contigs were then aligned using Se-Al version 2.0a11 (http://tree.bio.ed.ac.uk/software/seal/). To identify unique haplotypes, the contigs were clustered using a K-align rapid multiple sequence alignment (MSA) algorithm for DNA sequences (http://www.ebi.ac.uk/Tools/msa/kalign). Nucleotide sequence diversity among the *C*. *brevitarsis* haplotypes (i.e., Cbrev-01, …, Cbrev-09; 835 base pair (bp)), and among the *C*. *marksi* haplotypes Cmarksi-01, Cmarksi-03, …, Cmarksi-16 (547bp) were calculated as: (i) *p* distances (i.e., the proportion of sites with different nucleotides within pairs of sequences); or (ii) evolutionary distances using Tamura and Nei’s [57] nucleotide substitution model. In practice, we used MEGA version 5.05 [58] to obtain these estimates. Rate-heterogeneity across sites was modelled using a discrete Γ distribution with four rate categories and a shape parameter (α = 0.1759; model inferred using MODELTEST version 3.7 [59]). Standard errors of genetic distance estimates were obtained using nonparametric bootstrapping with 500 replicates. Mean genetic distances between *Culicoides* species were also obtained from an alignment of 455bp under the conditions described above for the within-species comparison. Unique haplotypes have been deposited in GenBank [GenBank accessions: KP201844-KP201872].

MtDNA COI sequences from *Culicoides* species previously deposited into GenBank, covering the same 547bp region as the *C*. *marksi* haplotypes reported in this study, were also included (Table 1) in our study in order to estimate nucleotide sequence divergence between and within species. GenBank entries were found using BLASTN [54] search with *Culicoides* as the Entrez [60] query against the Cmarksi-01 haplotype query sequence and specifying ‘Other (nr etc.)’ as the database.

### Comparison of *C*. *brevitarsis* from Australia, Solomon Islands, Timor-Leste, China and Japan

Twelve *C*. *brevitarsis* mtDNA COI sequences, ranging in length from 646bp to 703bp, were available through GenBank [51, 61]. Of these samples, two were from Japan [GenBank: AB360994, AB360995], one from Australia (Grafton [GenBank: KJ162974]), three from China (Hainan Province [GenBank: KJ162966, KJ162972, KJ162973]), four from Solomon Islands (Malaita [GenBank: KJ162971], Western Province [GenBank: KJ162970, KJ162969, KJ162967]), and two from Timor-Leste (Oecusse [GenBank: KJ162975], Cova Lima [GenBank: KJ162968]). These published mtDNA COI haplotypes allowed us to formulate different hypotheses regarding possible incursions of *C*. *brevitarsis* into Australia.

The 10 *C*. *brevitarsis* mtDNA COI sequences of Bellis *et al*. [51] represented four haplotypes, of which the samples from Grafton, Cova Lima, and the Solomon Islands represented one haplotype, the Chinese samples represented two haplotypes, and the sample from Oecusse represented the final haplotype. For the purpose of aligning the mtDNA COI haplotypes by Bellis *et al*. [51] with those generated by the present study, all *C*. *brevitarsis* sequences were trimmed either at the 5’ and/or 3’ regions, resulting in overlapping of either 323bp (China, n = 3; Timor-Leste, n = 2; Solomon Islands, n = 1), 335bp (Solomon Islands, n = 3), or 339bp (Australian, n = 1; Japanese, n = 2) with our *C*. *brevitarsis* haplotypes. Reducing the length of the alignment of our *C*. *brevitarsis* mtDNA COI region to nucleotide positions 1 to 339 (i.e., the shared region with GenBank samples) also reduced the total number of *C*. *brevitarsis* mtDNA haplotypes identified from nine to seven (i.e., Cbrev-01/05, Cbrev-02/08, Cbrev-03, Cbrev-04, Cbrev-06, Cbrev-07, Cbrev-09) (S4 Table).

The mtDNA COI haplotype of the individual from Grafton [GenBank: KJ162974] is likely to be Cbrev-01 or Cbrev-05 (due to the presence of a C in position 646, which it shares with Cbrev-01 and Cbrev-05, but not the other Australian sequences; S4 Table). To differentiate between Cbrev-01 and Cbrev-05 would require a C or a T at position 1,144, but this position was unfortunately not sequenced. Both Cbrev-01 and Cbrev-05 have been found at Grafton, so KJ162974 is most likely Cbrev-01 or Cbrev-05. The sequence from Grafton [KJ162974] was also identical over a shared 646-655bp region to those from the Solomon Islands [GenBank: KJ162971, KJ162970, KJ162969, KJ162967] and Timor-Leste [GenBank: KJ162968].

### MtDNA COI haplotype network

Haplotype networks were drawn using TCS version 1.21 [62] for the 835bp mtDNA COI haplotypes of *C*. *brevitarsis*, and for the 547bp mtDNA COI haplotypes of *C*. *marksi*. The resulting networks were subsequently refined by hand.

### Analysis of Australian *C*. *brevitarsis* population structure based on partial mtDNA COI gene sequence and EPIC-microsatellite DNA markers

#### (I) Inference of population structure based on mtDNA COI partial gene

Using 835bp of the partial mtDNA COI gene, we estimated genetic differentiation among and between *C*. *brevitarsis* populations across northern and eastern Australia using Weir and Cockerham’s [63] single locus *F*-statistics, as implemented in GENEPOP version 4.2 [64, 65]. Estimates of diversity indices (haplotype diversity (*h*) and nucleotide sequence diversity (π)) in *C*. *brevitarsis* (based on 835bp) and *C*. *marksi* (based on 547bp), and the Analysis of Molecular Variance (AMOVA) [66] for *C*. *brevitarsis* was performed using Arlequin version 3.11 [67]. To identify whether the Australian *C*. *brevitarsis* population could be subdivided into smaller groups, we performed an AMOVA whilst considering the following entities: (i) a single population (i.e., where all the Australian specimens of *C*. *brevitarsis* belong to a single population); (ii) a northern population (comprising specimens from Kalumburu, Douglas-Daly, and Katherine) and an eastern population (comprising specimens from Lismore, Grafton, Bellingen, and Paterson), and (iii) a large number of site-specific populations (i.e., where each collection site has its own population). The reason for assuming the northern and eastern populations was based on the general close proximity of collection sites to each other and did not reflect the midges’ flight capability, which is usually dependent on environmental factors (e.g., weather conditions/wind patterns [29, 68]). We also calculated the inter-population *F*_ST_ between the seven *C*. *brevitarsis* populations using GENEPOP (Option 6) to determine whether geographic distance between locations may have contributed to an underlying population structure; to investigate this, we plotted geographic distances (km, estimated from GPS data) against the *F*_ST_/(1- *F*_ST_) values.

#### (II) Inference of *C*. *brevitarsis* population structure based on nuclear markers

Using Arlequin, we also obtained results from an AMOVA based on the EPIC (RpS4, RpS2A, RpS2B, RpL27A, RpS6, and RpS8) and microsatellite (Cimi-69, and Cimi-85) markers. We excluded RpS4 and RpS2A and the two microsatellite loci from our analyses because: RpS4 and RpS2A were monomorphic; Cimi-66 was found to potentially contain a 5-bp (TTGCA) target-site duplication sequences surrounding the (CA)_n_ SSR units; and Cimi-12 amplified inconsistently with >3 alleles detected in various *C*. *brevitarsis* individuals (data not shown). Estimates of nucleotide sequence diversity (π), haplotype (gene) diversity (*h*), *F*_IS_ and their associated *P* values, pair-wise *F*_ST_ values between populations, average number of alleles (*a*), allele richness (*r*), and observed and expected heterozygosity estimates (H_O_, H_E_) across all nuclear markers for each population were also obtained using Arlequin, and departures from Hardy-Weinberg equilibrium were calculated using GENEPOP. Pair-wise *F*_ST_ estimates between populations with significant *P*-values were adjusted using the Holm-Bonferroni correction [69] at the family-wise error rate of α = 0.05. We determined the presence of isolation by distance for our *C*. *brevitarsis* populations using the transformed (i.e., *F*_ST_/(1-*F*_ST_)) pair-wise population *F*_ST_ estimates (i.e., Slatkin’s [70] linearized *F*_ST_), plotted against natural logarithm of geographic distances between locations of individual populations, with the significance of observed associations determined using Spearman Rank Correlation coefficient with 5,000 permutations, as implemented in GENEPOP.

### Structure analysis

We used the Bayesian MCMC simulation program STRUCTURE [71] to determine the most likely number of ancestral gene clusters based on the current population nuclear marker (four EPIC and two microsatellite) genetic data for *C*. *brevitarsis*. The rationale and parameters used to calculate the *ad hoc* statistic ΔK values (this provides an indication of the second-order rate-of-change to the estimated log probability of data Pr(X|K) between increasingly large values of K (i.e., number of genetic clusters)) were as described in Behere *et al*. [19]. Once the best ∆K values were found, a final simulation (comprising 500,000 MCMC samples and a burn-in of 50,000) was conducted to obtain the most likely ancestral population structure.

### Phylogenetic analysis

A phylogenetic tree was inferred using a multiple sequence alignment (MSA) of 182 codons from the mtDNA-encoded COI genes of the 136 *Culicoides* sequences (Table 1). Orthologous sequences from *Anopheles quadriannulatus* [GenBank: DQ792581], *A*. *gambiae* [GenBank: Nc_002084], *A*. *dirus* D [GenBank: AJ877572], and *A*. *janconnae* [GenBank: HQ335348] were aligned to these 136 sequences, allowing us to root of the inferred tree of *Culicoides*. The MSA (see S1 and S2 Files) was inferred using SeaView version 4.4.2 [72].

Initially, the MSA of codons was translated into amino acids and then partitioned into MSAs of codon sites. Whenever sequences in these four MSAs were found to be identical to one another, all but one of these sequences were removed to reduce bias in the phylogenetic estimates.

Most molecular phylogenetic methods assume that the sequences have evolved under globally stationary, reversible, and homogeneous (SRH) conditions (defined in [73–77]). To determine whether these conditions were met by the data, we assessed each alignment using a matched-pairs test of symmetry [73, 78]. In practice, we used Homo <www.csiro.au/homo>, a program that returns a χ^2^-distributed test statistic for all pairs of sequences. The corresponding *P* values were then charted in a PP-plot against the expected *P* values. A J-shaped distribution of dots in this plot, with a larger-than-expected proportion of low *P* values, indicates that the assumption of evolution under globally SRH conditions is violated by the data.

Given the results of our assessment of model misspecification (described above) and other considerations (described below), we conducted our phylogenetic analysis on the MSA of first and second codon sites. In practice, we used IQTREE version 0.9.3 [79] to find the most likely (ML) tree. In so doing, we first identified the best model of evolution for the data using the–m TESTONLY option. Next, we inferred the most likely tree and completed the phylogenetic analysis with a nonparametric bootstrap analysis (using the–bb 1000 option). The result was displayed using FigTree version 1.40 <http://tree.bio.ed.ac.uk/software/figtree>.

### Ethics

The authors declare that the research does not involve human subjects, human material or human data. The study also does not involve vertebrates or any regulated invertebrates, and therefore does not require any ethics committees’ approval.

## Results

### Analyses of the mtDNA marker from Australian *C*. *brevitarsis* and *C*. *marksi*

**Fig 2:**
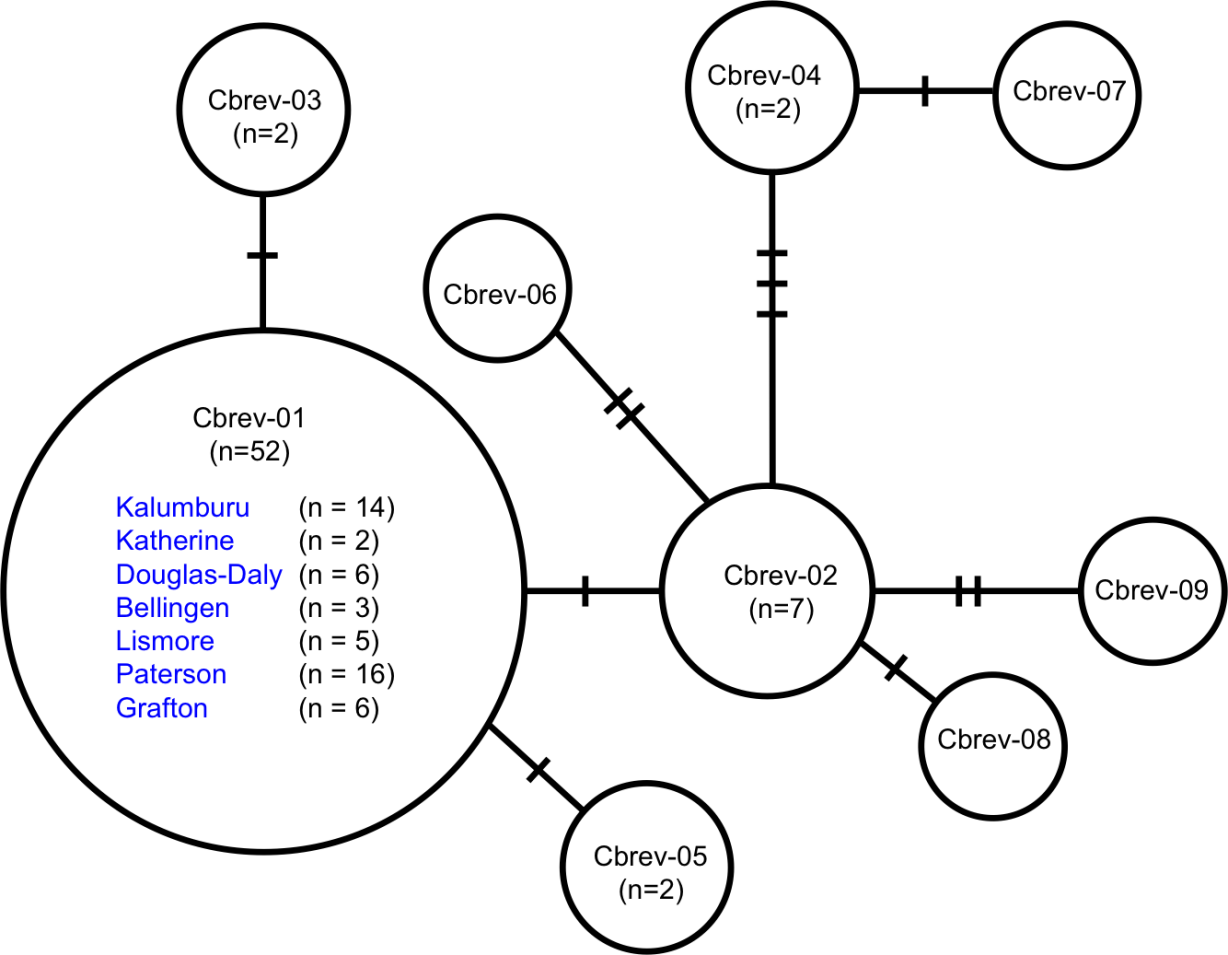
*Culicoides brevitarsis* mtDNA haplotype network based on 835 bp of mtDNA COI gene sequence. **2** *Culicoides brevitarsis* mtDNA COI haplotype network (835bp) from seven sites across Australia (Kalumburu, WA; Douglas-Daly and Katherine, NT; Lismore, Grafton, Bellingen, and Paterson, NSW). The total number of *C*. *brevitarsis* surveyed was n = 70. The most common haplotype is Cbrev-01 (it includes n = 52 individuals from all seven sites), followed by Cbrev-02 (n = 7) from Lismore, Grafton, and Paterson. Haplotypes Cbrev-03, Cbrev-04, and Cbrev-05 are represented by two individuals each; all remaining haplotypes are represented by one individual each. The haplotype network was generated using TCS version 1.21 [62], and then refined manually. The numbers of substitutions separating haplotypes are indicated by short bars.

A total of nine haplotypes was found in our alignment of 70 partial sequences (835bp) of the mtDNA COI gene from *C*. *brevitarsis*. The most common haplotype was Cbrev-01 (n = 52), followed by Cbrev-02 (n = 7), Cbrev-03 (n = 3), Cbrev-04 (n = 2), and Cbrev-05 (n = 2), while Cbrev-06, Cbrev-07, Cbrev-08, and Cbrev-09 were found once each (Table 1, Fig 1). Cbrev-01 was found at all sampling sites (Table 1, Figs 1 and 2) while Cbrev-03, Cbrev-04 and Cbrev-06 were found only at our sites in WA (Kalumburu) and NT (Douglas-Daly, and Katherine). The other haplotypes (Cbrev-02 (three sites), Cbrev-05 (two sites), Cbrev-07, Cbrev-08, and Cbrev-09 (one site each)) were found at our sites in NSW (Lismore, Grafton, Bellingen, and Paterson). Differences at six sites in the DNA separated the most diverged haplotypes (e.g., Cbrev-07 vs. Cbrev-03; Cbrev-07 vs. Cbrev-09; Cbrev-07 vs. Cbrev-06; Cbrev-07 vs. Cbrev-05: Fig 2). Estimates of the evolutionary distance between our Australian samples of *C*. *brevitarsis* were low, ranging from 0 to 0.7% (S1 Table). The corresponding estimates between the Australian and Japanese samples of *C*. *brevitarsis* [GenBank: AB360994.1, AB360995.1] [61] were, surprisingly, one order of magnitude higher (8.2% to 8.9%, S1 Table).

**Fig 3:**
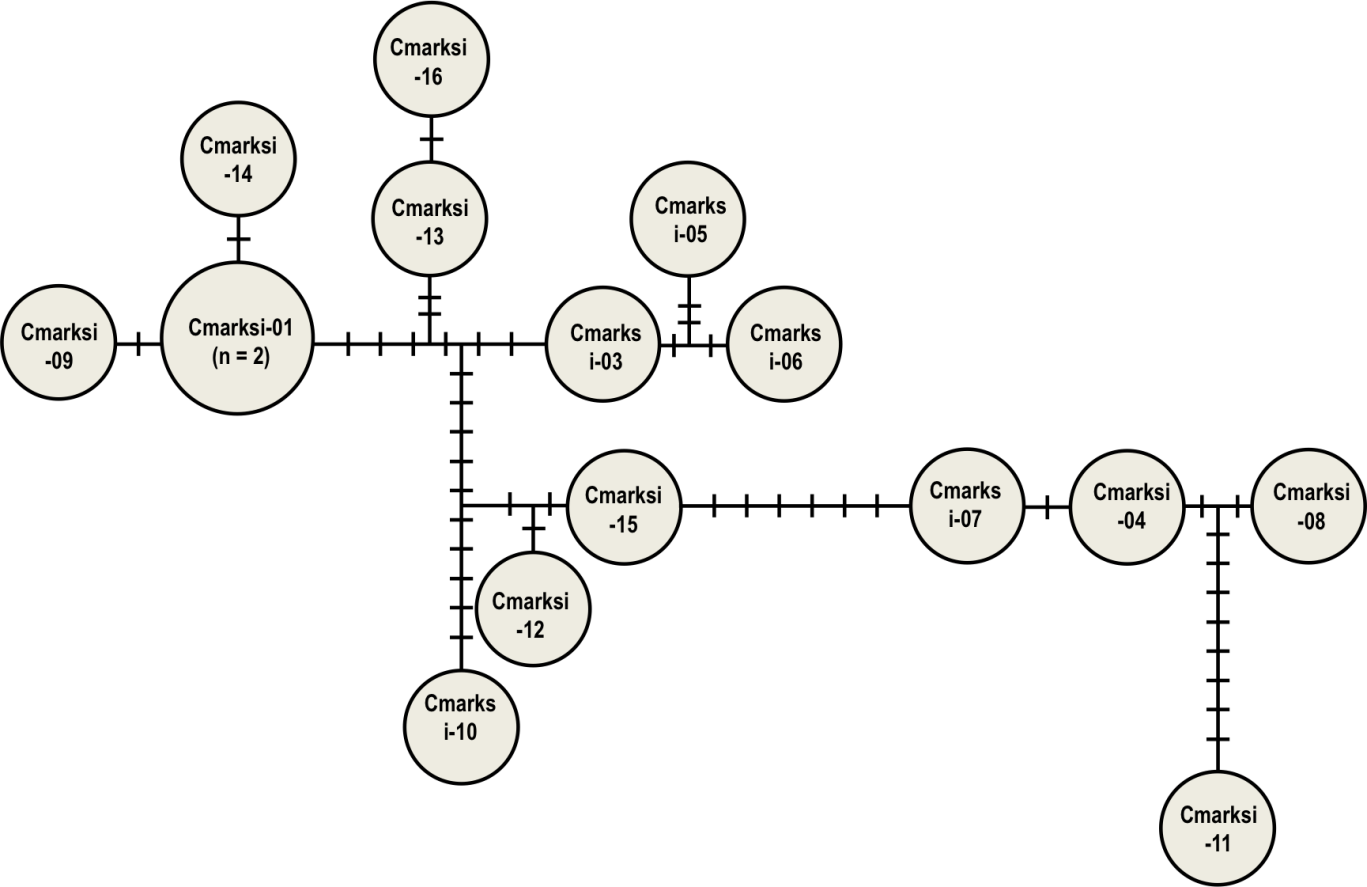
*Culicoides marksi* mtDNA haplotype network based on 547 bp of mtDNA COI partial gene sequence. *Culicoides marksi* mtDNA COI haplotype network (547bp) from Moree (NSW) and Murray Bridge (SA). The total number of *C*. *marksi* samples surveyed was 16. Haplotypes Cmarksi-01, and Cmarksi-03, …, Cmarksi-09 are from Moree, while Cmarksi-10, …, Cmarksi-16 are from Murray Bridge. The haplotype network was obtained using TCS version 1.21, and then refined manually. Apart from Cmarksi-01, which was represented by two individuals, the other haplotypes were represented by one individual each. The numbers of substitutions separating haplotypes are indicated by short bars.

A total of 15 haplotypes was found in our alignment of 16 partial sequences (547bp) of the mtDNA marker from *C*. *marksi* (all from Murray Bridge in SA and Moree in NSW. Two of the samples from Moree were identical; Figs 1 and 3), indicating a high level of genetic diversity among these samples. The evolutionary divergence between pairs of these sequences ranged from 0.0 to 2.8% (S2 Table), which is up to four times greater than those between the Australian *C*. *brevitarsis*.

The average genetic distances between all *Culicoides* species sampled in Australia and those available from GenBank (S5 Table) indicate high levels of genetic diversity between the species, with an average genetic distance of 0.153 ± 0.009 SE (range from 0.110 ± 0.014 SE (*C*. *pallidothorax* vs. *C*. *henryi*) to 0.264 ± 0.020 SE (*C*. *humeralis* vs. *C*. *actoni*)). When third codon sites were excluded (reason given below), the genetic distances between the species were much lower (average = 0.059 ± 0.007 SE; range from 0.007 ± 0.004 SE, (*C*. *kibunensis* vs. *C*. *verbosus*) to 0.172 ± 0.021 SE (*C*. *actoni* vs. *C*. *jacobsoni*)). The average genetic distances within species with at least two haplotypes ranged from 0.0 (e.g., in *C*. *wadai*) to 0.065 (in *C*. *matsuzawai*) across the trimmed 547bp region. Differentiation of *Culicoides* species based purely on mtDNA COI sequence divergence may be impossible (for a discussion, see Meier *et al*. [80]) due to over-lapping intra- and inter-specific genetic distances (i.e., the lower range between species is 0.096 (*C*. *pallidothorax* vs. *C*. *henryi*) while the maximum intra-specific genetic distance is 0.082 (e.g., *C*. *brevitarsis*) (S5 Table), a difference of only 1.4%).

Estimates of haplotype diversity (*h*) and nucleotide sequence diversity (π) were both higher for the Australian endemic *C*. *marksi* (*h* = 0.976 ± 0.012; π = 0.017 ± 0.001) than for the Australian *C*. *brevitarsis* (*h* = 0.586 ± 0.172; π = 0.001 ± 0.001). We did not detect any isolation-by-distance effect between the Australian populations of *C*. *brevitarsis* based on the mitochondrial DNA COI partial gene marker, with these populations lacking significant substructure as indicated by a low *F*_ST_ value (*F*_ST_ = 0.026), and non-significant correlation between geographic distances and *F*_ST_/(1 –*F*_ST_) values (*R*^2^ = 0.0016), possibly due to the wide geographical distribution of the Cbrev-01 haplotype (Fig 1). Hierarchical population structures were not found using AMOVA-estimated fixation indices: *F*_CT_ (among groups), *F*_SC_ (among populations within groups) and *F*_ST_ (within populations) (none of the associated *P*-values were significant, Table 3). The AMOVA showed that 96.6% of the variance occurs at the within-populations level, while 1.8% and 1.6% of the variance were at the among-groups level (i.e., within Australia) and among-populations within-groups level (i.e., between northern and eastern populations), respectively.

**Table 3:** AMOVA results of Australian *Culicoides brevitarsis* populations based on mtDNA and nuDNA markers.

Hierarchical levels	Variance	Percentage of variation	Fixation indices	*P*-value
**(a)**				
Among groups (*F*_CT_)	0.0001	0.03	0.0003	0.431±0.014
	0.004	(1.78)	(0.018)	(0.305±0.015)
Among populations within groups (*F*_SC_)	0.023	5.18	0.052	0.064 ± 0.009
	(0.004)	(1.64)	(0.017)	(0.297±0.014)
Within populations (*F*_IT_)	0.415	(94.79)	0.0518	0.107 ± 0.009
	(0.227)	(96.58)	(0.034)	(0.225±0.014)
**(b)**				
Among groups (*F*_CT_)	0.003	0.46	0.005	0.235±0.014
Among populations within groups (*F*_SC_)	0.014	2.00	0.020	**0.047±0.008**
Among individuals within populations (*F*_IS_)	0.030	4.21	0.043	0.172±0.010
Within individuals (*F*_IT_)	0.665	93.32	0.067	0.088±0.009

Results from Analysis of Molecular Variance (AMOVA) of *Culicoides brevitarsis* populations in Australia based on (a) mtDNA COI partial gene sequence 829bp (502bp in parentheses for comparisons with *C*. *marksi*), and (b) four EPIC and two microsatellite DNA loci.

**Note:** Fixation indices based on mtDNA COI partial sequence for: among groups (*F*_CT_) are northern Australian (Kalumburu, Douglas-Daly, and Katherine) and eastern Australian sites (Lismore, Grafton, Bellingen, and Paterson); among populations within groups (*F*_SC_), and within populations (*F*_ST_). All *P* values were non-significant. Fixation indices estimated from EPIC-PCR and microsatellite DNA markers were also non-significant for among groups (*F*_CT_), for among individuals within populations (*F*_IS_, i.e., non-random mating among individuals within subpopulations), for *F*_IT_ (i.e., average reduction in heterozygosity of an individual relative to the total population, and represents contributions due to non-random mating within demes (subpopulations) and effects of random genetic drift among demes (*F*_ST_)), but significant for among populations within groups (*F*_SC_; *P*-value = 0.047 ± 0.008), indicating substantial variance existed at this population structure hierarchical level.

### Analyses of the nuDNA markers from Australian *C*. *brevitarsis*

Two exon-primed intron-crossing (EPIC) DNA markers (i.e., RpS4 and RpS2A) were found to be monomorphic across all the populations of *C*. *brevitarsis* that we screened. We sequenced the amplicons of RpS4 and RpS2A from individuals of *C*. *brevitarsis*, confirming the primer annealing sites and the partial coding region sequences for RpS4 and RpS2A. Despite the expected presence of an intron within the sequences examined when compared against the *Bombyx mori* homologs, the expected introns in these two Rp gene regions were missing (sequences available on request), providing the reason for the lack of polymorphisms for these two EPIC DNA markers. This, therefore, leaves the availability of four EPIC markers (i.e., RpS2B, RpS6, RpS8, RpL27A) as well as two microsatellite DNA markers (i.e., Cimi69, Cimi85) to infer the population genetic structure of *C*. *brevitarsis*.

The results obtained from the AMOVA of the six nuDNA markers from *C*. *brevitarsis* were largely in agreement with those obtained from the mtDNA marker, with the exception that the fixation index for the among populations within groups comparisons (*F*_SC_ = 0.020) was significant at both the mean and lower estimates, but not at the higher estimate (*P* value = 0.047 ± 0.008). The availability of results from analyses of nuDNA markers enabled a fine-scale investigation of within population variation, which showed that 93.3% of the variation was at the within individual level relative to the total population (*F*_IT_) level (Table 3). Pair-wise *F*_ST_ estimates between all populations obtained using the mtDNA marker did not reveal any population substructure (Table 4) due to the presence of the Cbrev-01 haplotype in all populations. By contrast, the nuDNA markers disclosed significant signatures of limited gene flow between the northern region populations (e.g., Kalumburu B, Douglas-Daly) and four of the southern region populations (Table 5; six out of 28 comparisons). However, following a Holm-Bonferroni correction, only two pair-wise *F*_ST_ estimates were significant (i.e., Douglas-Daly vs. Lismore; Douglas-Daly vs. Paterson; Table 5). Based on the *F*_IS_ estimates (Table 6), no significant inbreeding at the within subpopulation level could be detected.

**Table 4:** Australian *Culicoides brevitarsis* population mtDNA COI pairwise *F*_ST_ estimates and associated *P* values ± SE.

Populations	1	2	3	4	5	6	7
**1. Kalumburu**	-	0.999±0.001	0.538±0.015	0.999±0.001	0.121±0.010	0.999±0.001	0.429±0.012
**2. Douglas-Daly**	-0.059	-	0.484±0.014	0.999±0.001	0.101±0.010	0.999±0.001	0.758±0.013
**3. Katherine**	-0.019	0.018	-	0.570±0017	0.534±0.015	0.999±0.001	0.090±0.009
**4. Lismore**	-0.055	-0.044	-0.028	-	0.428±0.017	0.999±0.001	0.760±0.012
**5. Grafton**	0.092	0.122	0.008	-0.021	-	0.450±0.014	0.077±0.009
**6. Bellingen**	-0.088	-0.067	-0.067	-0.105	0.027	-	0.363±0.016
**7. Paterson**	-0.018	-0.040	0.163	-0.041	0.117	-0.020	-

Note: Australian *Culicoides brevitarsis* population mtDNA COI pairwise *F*_ST_ estimates (lower triangle) and associated *P* values ± SE (upper triangle) based on 829bp of mtDNA COI partial gene sequence. Intermediate level of population substructure is detected between Grafton and Douglas-Daly (*F*_ST_ = 0.122) and between Grafton and Paterson (*F*_ST_ = 0.117) but with non-significant *P* values and SD from 500 bootstrap replications.

**Table 5:** Australian *Culicoides brevitarsis* population pairwise *F*_ST_ estimates and associated *P*-values inferred from nuDNA markers.

		1	2	3	4	5	6	7	8
1	Kalumburu A	-	0.261 ± 0.044	0.054 ± 0.020	0.072 ± 0.018	0.541 ± 0.054	0.676 ± 0.046	0.496 ± 0.039	0.937 ± 0.028
2	Kalumburu B	0.027	-	0.450 ± 0.073	0.532 ± 0.032	0.721 ± 0.045	0.036 ± 0.015	0.198 ± 0.033	0.252 ± 0.049
3	Katherine	0.057	0.015	-	0.009 ± 0.009	0.369 ± 0.042	0.306 ± 0.015	0.505 ± 0.039	0.009 ±0.009
4	Douglas-Daly	0.092	0.007	0.157	-	0.045 ± 0.020	**0.000 ± 0.000**	**0.000 ± 0.000**	0.054 ± 0.020
5	Bellingen	-0.008	-0.011	0.002	0.075	-	0.369 ± 0.055	0.496 ± 0.051	0.387 ± 0.078
6	Lismore	-0.015	0.087	0.018	**0.216**	0.005	-	0.820 ± 0.037	0.180 ± 0.043
7	Paterson	-0.003	0.027	0.001	**0.119**	-0.005	-0.015	-	0.216 ± 0.053
8	Grafton	-0.029	0.016	0.076	0.056	0.000	0.025	0.010	-

Pairwise *F*_ST_ estimates (lower triangle) and corresponding *P* values (upper triangle) between *Culicoides brevitarsis* populations as inferred based on genotype data from four EPIC and two microsatellite DNA markers. Significant *F*_ST_ *P* values (< 0.05) are in red based on 110 permutations as calculated using Arlequine V3.11. Kalumburu A and Kalumburu B are two Western Australian populations collected from the same sites but from different years (‘A’ 29/12/08; ‘B’ 25/6/09). Katherine and Douglas-Daly are from the Northern Territory, and Bellingen, Lismore, Paterson and Grafton are New South Wales populations.

**Note:** Only *F*_ST_ estimates for Douglas-Daly/Lismore and Douglas-Daly/Paterson remained significant following a Holm-Bonferroni correction at the α = 0.05 level (bolded). Treating Kalumburu A & B as a single population (due to the insignificant *F*_ST_ value between these two samples) indicated the *F*_ST_ estimate between Kalumburu and Lismore was not significant (*F*_ST_ = 0.029, *P* = 0.135±0.023).

**Table 6:** Nuclear DNA marker statistics for Australian *C*. *brevitarsis* populations.

Locations	n	a	r	*H*o	*H*e	*F*_IS_	*P*
Kalumburu A	8	3.167 ± 1.344	0.443 ± 0.289	0.521 ± 0.357	0.488 ± 0.305	0.400	0.098
Kalumburu B	8	3.000 ± 1.291	0.638 ± 0.473	0.578 ± 0.320	0.526 ± 0.277	0.228	0.094
Douglas-Daly	6	2.833 ± 1.344	0.568 ± 0.377	0.461 ± 0.360	0.494 ± 0.351	-0.059	0.779
Katherine	4	3.333 ± 2.055	0.801 ± 0.557	0.602 ± 0.308	0.572 ± 0.278	0.103	0.257
Bellingen	3	3.667 ± 2.494	0.732 ± 0.514	0.535 ± 0.342	0.611 ± 0.173	-0.001	0.556
Lismore	6	2.500 ± 1.893	0.000 ± 0.000	0.563 ± 0.419	0.599 ± 0.431	0.034	0.433
Paterson	20	4.500 ± 2.063	0.659 ± 0.422	0.582 ± 0.335	0.591 ± 0.136	-0.063	0.525
Grafton	12	2.000 ± 2.828	0.669 ± 0.473	0.217 ± 0.313	0.223 ± 0.319	0. 022	0.562

Australian *Culicoides brevitarsis* population statistics based on four EPIC-PCR and two microsatellite DNA loci.

**Note:** n = number of individuals analysed; a = average number of alleles (± s.d.), r = allele richness (average gene diversity over loci), *H*_o_ = observed heterozygosity, *H*_e_ = expected heterozygosity, *P* (Random *F*_IS_ ≥ observed *F*_IS_). Markers were overall in Hardy Weinberg equilibrium for all populations with non-significant *P* values. All *F*_IS_ estimates for all populations were also not significant, although Kalumburu (A & B) *F*_IS_ estimates were high, but low (*F*_IS_: 0.087, *P* = 0.252) when both populations combined as one.

Significant isolation-by-distance was detected using the nuDNA markers (R^2^ = 0.192, *P* value = 0.0152), indicating that neighbouring populations tended to be genetically more similar to one another than they are to distant populations. Overall, the six nuDNA markers appeared to assort independently, and using Fisher’s exact test did not disclose any departure from Hardy-Weinberg equilibrium, the only exceptions being Cimi-85 for Kalumburu and Douglas-Daly, and RpS6 and RpL27A for Paterson (due to excess of homozygotes; results not shown).

### STRUCTURE analysis

**Fig 4:**
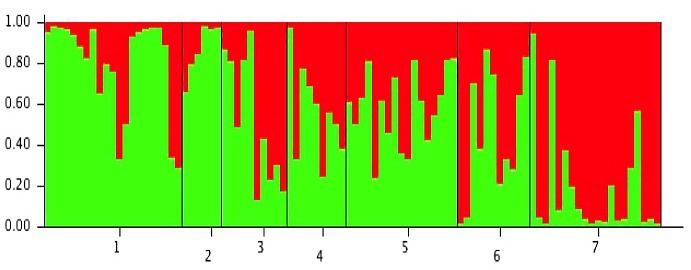
*Culicoides brevitarsis* population structure as inferred from nuDNA (EPIC-PCR and microsatellite DNA) markers using STRUCTURE 2.3. *Culicoides brevitarsis* population structure as inferred using the Bayesian clustering algorithm implemented in the program STRUCTURE 2.3 [71], based on nuclear DNA (four EPIC and two microsatellite) markers. Bar graphs are from *K* = 2 from 50,000 burn-in cycles and 500,000 runs. Populations are numbered from northern to southern regions, and combining (Kalumburu A and B (WA)) as one population (1), Douglas-Daly (2) and Katherine (3) from NT, and Lismore (4), Grafton (5), Bellingen (6), and Paterson (7) from NSW.

Using the method of Evanno *et al*. [81] to calculate the number of genetically homogenous groups of individuals in a population, we found that the nuDNA markers data (Fig 4) was best explained as two genetically homogeneous sets of individuals (∆K = 8.81). We did not apply the mtDNA data to the STRUCTURE analysis as this method is best applied to sequence data with sufficient recombination rates (i.e., its application based on non-recombination genomes such as the sex chromosomes (i.e., Y-chromosome) and genomes with predominantly uni-parental inheritance mode (i.e., chloroplast or mitochondrial DNA) should be interpreted with caution [71]). The STRUCTURE result suggests the Australian *C*. *brevitarsis* population structure was best explained by unequal contributions from two genetically distinct ancestral clusters, with the ‘Red’ and ‘Green’ genetic clusters following latitudinal clines from the north-western/northern populations to the south-eastern/southern populations, although increasing sampling sites and marker numbers will be needed to better explore this genetic signature in future studies. This analysis therefore shows that although the northern and southern *C*. *brevitarsis* populations are the same species and that gene flow has occurred between them, there is also evidence that they represent two overall genetic groups of individuals (exemplified by differences between populations in the north (e.g., Kalumburu) and populations in the south (e.g., Paterson)). This may have implications for the capacity of the BTV to spread (see Discussion).

### Phylogenetic analysis

**Fig 5:**
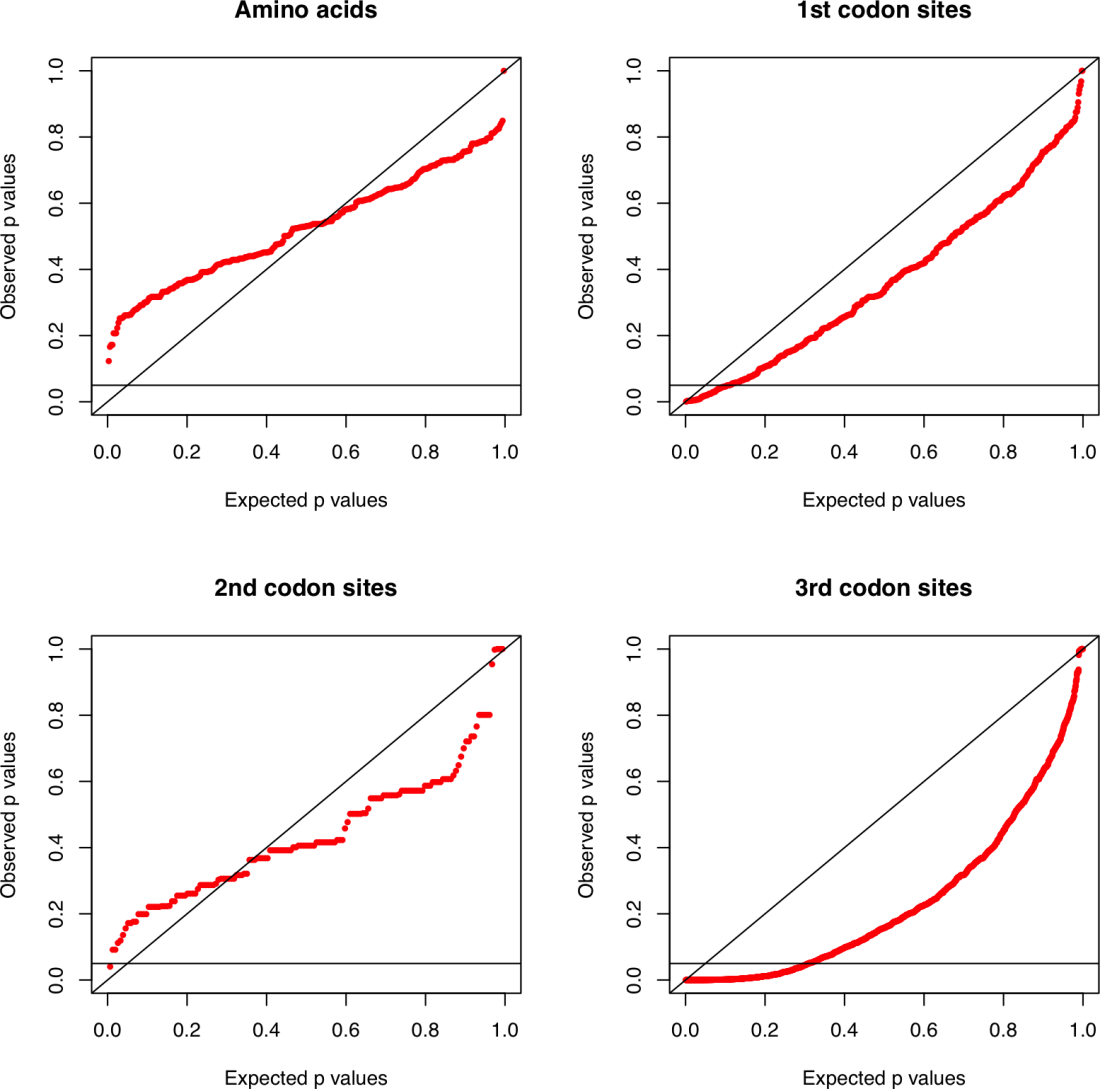
PP-plots for *P* values distributions from matched-pairs tests of symmetry for mtDNA COI sequence data sets. PP-plots with the distributions of *P* values from the matched-pairs tests of symmetry for the following data sets: amino acids, first codon sites, second codon sites, and third codon sites. A J-shaped distribution of dots, with more than 5% of the dots below 0.05 (i.e., the horizontal line) indicates violation of the assumption of evolution under globally SRH conditions. The PP-plot for third codon sites represents a clear example of violation of this phylogenetic assumption.

Fig 5 shows four PP-plots with results from the matched-pairs test of symmetry for the four MSAs of amino acids, first codon sites, second codon sites, and third codon sites. In terms of being suitable for phylogenetic analysis using time-reversible Markov models, the data sets are ranked (from best to worst): amino acids, second codon sites, first codon sites, and third codon sites. Thirty-one per cent of the tests led to *P* values below 0.05 for third codon sites (5% was expected), and the lowest observed *P* value was 1.33 × 10^−9^, which still is significant after a Holm-Bonferroni correction [69] to counteract the effect of multiple comparisons (*P*_cor_ = 2.76 × 10^−6^). For first codon sites, the corresponding values were 10.7% and *P*_cor_ = 0.5395. These results show that it would be unwise to use third codon sites in a phylogenetic study that assumes time-reversibility.

The numbers of phylogenetically informative sites were 44, 49, and 13 for the MSAs of amino acids, first codon sites, and second codon sites, respectively, implying that it would be preferable to use MSAs of first and second codon sites to infer the phylogenetic tree. In order to increase the number of phylogenetically informative sites in the MSA used to infer the phylogenetic tree, we concatenated the MSAs for these two codon sites.

Using IQTREE, we found the best model of evolution to be TN+I+G4, implying that the accumulating nucleotide substitutions was best approximated by Tamura and Nei’s [57] model of nucleotide substitution, with rate-heterogeneity across sites modelled assuming a proportion of invariable sites (I) and a discrete Γ distribution with 4 rate categories (G4). However, in practise the phylogenetic analyses were conducted under the TN+I+G4 and TN+G4 models to accommodate a known difficulty in estimating both I and Γ accurately (see page 114 in [82]).

**Fig 6:**
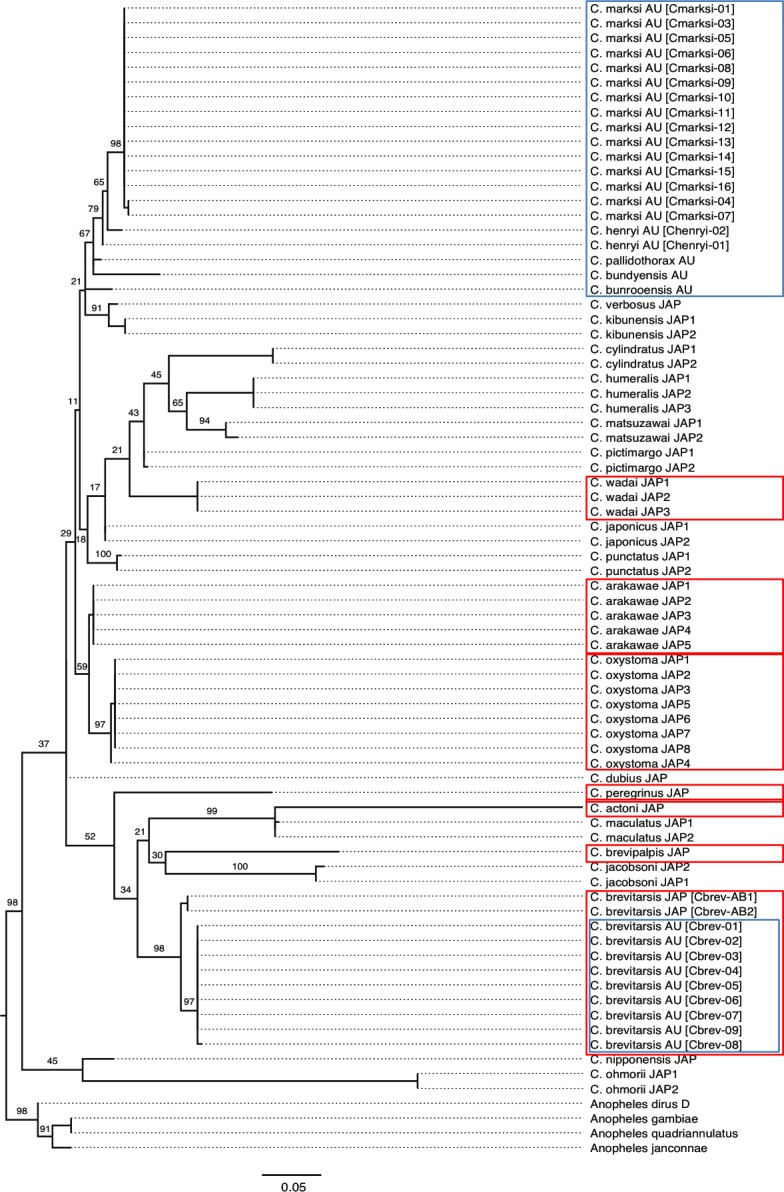
Maximum Likelihood phylogenetic tree including bootstrap values of various *Culicoides* species from this study. Maximum Likelihood tree, with bootstrap values for some of the internal edges inferred using the UFBoot method. The tree was inferred under the TN+I+G4 model of evolution. Samples are marked using their species name, geographical origin (AU: Australia; JAP: Japan), haplotype name (when available, listed in brackets), and a number (whenever needed, to distinguish some of the sequences). Australian *Culicoides* are within blue boxes, and known BTV competent Asian/South East Asian *Culicoides* hosts are in red boxes. The scale bar measures evolutionary distance in terms of average nucleotide substitutions per site. The tree was rooted using four species of *Anopheles*. The ML tree inferred under the TN+G4 model of evolution differed from this one at seven internal edges, but the overall conclusion remained the same (see text).

Fig 6 presents the ML tree inferred from the concatenated alignment of first and second codon sites. The tree divides the Australian samples into two distantly related groups. The first group includes the Australian *C*. *brevitarsis*, which consistently (97%) formed a sister group to the Japanese *C*. *brevitarsis*. The second group includes *C*. *marksi*, *C*. *henryi*, *C*. *pallidothorax*, *C*. *bundyensis*, and *C*. *bunrooensis*, which are endemic Australian species. However, this group may also include two Japanese species (*C*. *verbosus* and *C*. *kibunensis*). Whether this is the case was impossible to determine from the data (the low bootstrap values for some of the edges and the presence of trifurcations in the tree indicate that the number of informative sites is too low to cast light on this issue). Notwithstanding this shortness of data, it is worth mentioning the evolutionary distance between Australian and Japanese samples of *C*. *brevitarsis* is as large as those between some of the other Australian endemic species (i.e., *C*. *marksi*, *C*. *henryi*, and *C*. *pallidothorax*). Looking at the phylogenetic tree more broadly, it is characterised by many low bootstrap values and large variation in the root-to-tip distances. Taken together, these features are consistent with the number of phylogenetically informative sites being too small, implying that much longer sequences are needed to accurately resolve the phylogenetic relationships among these species.

With respect to the phylogenetic relationship among competent and potential vectors of BTV in Australia, our tree suggests that the five endemic species considered here (i.e., *C*. *marksi*, *C*. *henryi*, *C*. *pallidothorax*, *C*. *bundyensis*, and *C*. *bunrooensis*) do not share a close common ancestor with BTV-competent vectors (i.e., *C*. *brevitarsis*, *C*. *wadai*, *C*. *fulvus*, and *C*. *actoni*) (note that we did not have access to Australian samples of *C*. *fulvus*, *C*. *wadai*, and *C*. *actoni* and, therefore, were unable to determine whether Japanese samples of *C wadai* and *C*. *actoni* are the closest relatives of, respectively, the Australian samples of *C wadai* and *C*. *actoni*). Although this does not guarantee that these endemic species cannot become vectors of BTV, it does imply that they are so evolutionarily different from known vectors of BTV in Australia, that they might not be potential vectors of BTV. To better address this issue, it will be necessary to consider much longer sequences (e.g., preferentially the whole mitochondrial genome; multi-loci sequence analysis) and more samples and species from, in particular, Australia.

### Origin(s) and hypothesised incursion routes of Australian *C*. *brevitarsis*

Our study also considered previously reported samples that were collected in limited numbers from neighbouring countries such as Solomon Islands (n = 4; GenBank: KJ162971, KJ162970, KJ162969, KJ162967), Timor-Leste (n = 2; GenBank: KJ162975, KJ162968), China (n = 3; GenBank: KJ162966, KJ162972, KJ162973), and Japan (n = 2; GenBank: AB360994, AB360995). In addition, we considered an Australian individual sampled from Grafton (GenBank: 162974), a site also used by us.

A comparison of Australian, Japanese, Chinese, Solomon Islands, and Timor-Leste *C*. *brevitarsis* mtDNA COI sequences, available through the studies of [61] and [51], showed that the Japanese samples, and to a lesser extent, the Chinese samples, are distantly related to the Australian, Solomon Islands, and Timor-Leste samples (S1–S3, S5 Tables). On the other hand, one of the two haplotypes from Timor-Leste [KJ162968] was identical across at least 646bp to an Australian [KJ162974] and all four Solomon Islands individuals [51]. This haplotype [KJ162968] was also identical to our Cbrev-01 and Cbrev-05 haplotypes between positions 324 and 646, and is thus consistent with the expected introduction scenario for this haplotype (i.e., Cbrev-01), with an incursion route from Timor-Leste to the northwest of Australia, based on the prevailing weather/wind patterns. In order to differentiate the most common haplotype previously found [51], the authors would need to have sequenced further along the COI gene, as was done by us for Cbrev-01 and Cbrev-05, which identified a single base pair difference at position 1144 (S4 Table).

## Discussion

### *Culicoides brevitarsis* mtDNA COI haplotypes

Our analysis of the mtDNA marker show that the *C*. *brevitarsis* samples from northern and southern Australian sites (i.e., the full geographical range of *C*. *brevitarsis* in Australia) is a single species with low nucleotide sequence and haplotype diversities across >800bp of this genome. There is no support for the notion that *C*. *brevitarsis* in Australia is a set of cryptic species. *Culicoides brevitarsis* has been recorded in Australia for over 100 years and is believed to be an introduced species [83] of Asian or South East Asian origin [39]. The spread of *C*. *brevitarsis* to its current southern limit is likely to be linked to European settlements, and its presence in northern Australia may also be associated with the introduction of ruminants [84] such as water buffalo (*Bubalus bubalis*) since around 1825<https://www.environment.gov.au/biodiversity/invasive-species/publications/factsheet-feral-water-buffalo-bubalus-bubalis>. The inclusion of additional samples from South East Asia (e.g., Papua New Guinea, Timor-Leste, Indonesia, Malaysia, the Malay Archipelago), Micronesia, Melanesia, and Asia (e.g., India) would help to clarify what are the most likely origins of the Australian *C*. *brevitarsis* populations. However, the difficulty in obtaining good samples from these regions probably represents the single most challenging factor to future similar studies.

A total of nine mtDNA haplotypes were found among 70 *C*. *brevitarsis* individuals from three north Australian sampling sites (Kalumburu, Katherine, and Douglas-Daly) and four east Australian sampling sites (Lismore, Grafton, Bellingen, and Paterson) (Fig 1). With the exception of Cbrev-01, a haplotype found at all seven sampling sites (spanning distances of >3,000km), the remaining haplotypes could be split into northern haplotypes (Cbrev-03, Cbrev-04, and Cbrev-06) and eastern haplotypes (Cbrev-02, Cbrev-05, Cbrev-08, and Cbrev-09) (Fig 1). The detection of such distinct distributional differences may have biosecurity implications to Australia such as detecting historical pathways of incursion (discussed later).

The mtDNA COI gene has been widely used to differentiate insect species (e.g., [1, 13, 85]). Differentiating dipteran species based on the mtDNA COI gene generally has had a low success rate due to overlapping ranges of genetic distances during intraspecific and interspecific comparisons [80]. Differentiating *Culicoides* species using genetic distances of the mtDNA COI gene may therefore also be of limited value due to overlapping intra- and inter-specific genetic distances (e.g., the lower range between *C*. *henryi* and *C*. *pallidothorax* is 0.096 while the maximum intra-specific genetic distance between individuals of *C*. *brevitarsis* is 0.082 (S5 Table); a difference of only 1.4%). To better understand the genetic relationship between *C*. *pallidothorax* and *C*. *henryi*, ecological, behavioural, and geographical information will be needed in addition to morphological and sequence data (e.g. integrative taxonomy, see [86, 87]). A high intraspecific distance (> 0.110) was also reported for *C*. *obsoletus* and *C*. *newsteadi*, while the mtDNA barcode method failed to identify two closely related pairs of species as four separate species (i.e., *C*. *festivipennis* and *C*. *clastrieri* (0.001); *C salinarius* and *C*. *manchuriensis* (0.017)) due to low interspecific distances [35].

### *Culicoides marksi* mtDNA COI haplotypes

Analysis of 16 endemic *C*. *marksi* individuals from two populations detected greater haplotype diversity compared with all *C*. *brevitarsis* samples combined, and therefore adds support to the introduced species status of *C*. *brevitarsis*. *Culicoides marksi* is an important vector of many orbiviruses (e.g., Tables 1 and 2 in [88]). The species feeds readily on marsupials, and is distributed across the whole of Australia and into Papua New Guinea. A rare wing pattern variant of *C*. *marksi* has been found in the Northern Territory [27]. This, and the high level of genetic variation present in *C*. *marksi* populations, suggests that a future study should consider examining the likely presence of cryptic species within the currently recognised *C*. *marksi* species, as this might have been an important factor that contributed to previous unsuccessful artificial BTV infection experiments [88]. The high level of mtDNA haplotype diversity in *C*. *marksi* contrasted with that found in *C*. *brevitarsis*. Factors contributing to these differences between the two species are likely to include species-specific differences in flight patterns, dispersal ability, host-assisted or host-mediated dispersal, and preferred environmental/weather conditions and breeding sites (e.g., [41, 89–91]).

### *Culicoides brevitarsis* gene flow patterns inferred from nuDNA markers

Although the mtDNA genome is well suited for studying maternal gene flow patterns, inference of paternal gene flow patterns requires the inclusion of nuDNA markers (e.g., microsatellite and EPIC DNA markers). In this regard, EPIC DNA markers have not been used as widely as microsatellite DNA markers (e.g., [52]), but they are still highly suitable for inferring population genetic structures (e.g., [19]), especially in taxa with transposable elements (TEs) in their genomes, such as the Diptera (e.g., [53] and references therein). Here we demonstrated the usefulness of EPIC DNA markers, in addition to microsatellite DNA markers, in a study of gene flow patterns in Australian *C*. *brevitarsis*. EPIC and microsatellite DNA markers identified a population structure and gene flow patterns that are consistent with those detected using the mtDNA marker, with additional power to detect isolation by distance and genetic clusters in a species with limited polymorphism in its mtDNA. The discovery of isolation by distance using nuDNA markers but not mtDNA markers may imply sex-specific differences in dispersal patterns, feeding and/or mating behaviours in *C*. *brevitarsis*, although confirmation of this will require further work. Our STRUCTURE analysis of the nuDNA markers revealed the presence of a northern cluster and a south cluster and also supported the discovery of isolation by distance, with north-western populations (e.g., Kalumburu) and south-eastern populations (e.g., Paterson) being genetically less similar to each other that they are to their neighbouring populations. Two potential genetic clusters of *C*. *brevitarsis* from Australia and its neighbouring regions were also identified based on multiple microsatellite DNA markers by Onyango et al. [92], although this study would have benefited also from the inclusion of the maternally inherited mtDNA marker, for inference of genetic structure and incursion pathways from the maternal lineage perspective, given that it is the female species that is the main arbovirus vector.

### Phylogeny of *Culicoides*

The alignment of 182 codons encoding some of the mtDNA CO I gene from *Culicoides* was characterised by compositional heterogeneity across the sequences at third codon sites, but not at first and second codon sites, implying that the mitochondrial genomes of many of the 25 species examined here must have evolved under non-stationary, reversible, and homogeneous (SRH) conditions, and that natural selection must have been sufficiently strong to hide evidence of this at the non-synonymous codon sites. This is consistent with reports on the effects of directional mutation pressure on non-synonymous codon sites of protein-coding genes in animal mitochondria [93]. Since compositional heterogeneity across sequences may bias phylogenetic estimates [94] and most model-based molecular phylogenetic methods assume that the data have evolved under globally SRH conditions, we decided to use first and second codon sites in our phylogenetic analysis of *Culicoides*, even though the number of phylogenetically informative sites was relatively small for these two codon sites. This type of consideration is not as uncommon as it used to be (for examples, see [95, 96] and papers cited therein).

The inferred phylogeny of the mtDNA COI marker from our *Culicoides* samples was characterised by large variation in the root-to-tip distances and bootstrap values ranging from 11% to 100%, which is consistent with the data containing too few phylogenetically informative sites. Therefore, it was difficult to have much confidence in the inferred phylogenetic tree (Fig 6). However, assuming the tree is a good representation of the underlying evolutionary history of *Culicoides*, it would appear that the Australian species are divided into two groups, with *C*. *brevitarsis* being more closely related to species found in Japan (e.g., *C*. *jacobsoni*, *C*. *brevipalpis*, *C*. *maculatus*, *C*. *peregrinus*, and *C*. *actoni*) than species found in Australia, and with the remaining Australian species (*C*. *marksi*, *C*. *henryi*, *C*. *pallidothorax*, *C*. *bundyensis*, and *C*. *bunrooensis*) forming a closely related group of species.

### Biosecurity implications of *C*. *brevitarsis* northern and eastern mtDNA COI haplotypes

Dyce [39] suggested that Asian or South East Asian species such as *C*. *brevitarsis*, *C*. *oxystoma* and *C*. *peregrinus* may have arrived in Australia after early European settlement and the establishment of domesticated herbivores in the tropical north, with the proposed path to colonisation of *C*. *brevitarsis* being from Timor-Leste into the northern regions of WA, NT, and Queensland (QLD) prior to splitting into (i) a northern route (to colonise northern QLD and Papua New Guinea, and across to the Melanesian islands (e.g., Solomon Islands, Fiji, and maybe New Caledonia)), and (ii) a southern route (to colonise central QLD, northern and central NSW, and Lord Howe Island) (see Fig 2 of [97]). Using simulation studies, Eagles *et al*. [90] found that if the potential source of the Australian population were Timor-Leste, then, given the current climatic conditions and weather patterns, the Australian regions with the greatest risk of windborne incursion would be the top end of NT and the Kimberley region of WA. Our analysis of *Culicoides* mtDNA haplotypes from Australia, Timor-Leste, and the Solomon Islands is consistent with the conclusions of Eagles *et al*. [90].

The detected distributional patterns of *C*. *brevitarsis* mtDNA haplotypes and nuclear genetic clusters imply that multiple independent incursions may have occurred into Australia after the arrival of suitable domesticated cattle hosts (i.e., since European settlement began in Australia). Similarly, Bellis et al. [98] also identified evidence to support multiple incursion pathways into Australia from surrounding regions from mtDNA marker analysis. Based on our comparisons of mtDNA haplotypes from neighbouring regions and throughout Australia, four hypotheses can be put forward to assist future in-depth studies of *C*. *brevitarsis* incursion pathways and population spread: (i) the presence of a single mtDNA haplotype (Cbrev-01) in Timor-Leste and across the distributional range of *C*. *brevitarsis* in Australia potentially indicates that populations in Timor-Leste may have been the source of Australian populations, and that members of these populations may have been carried by trade winds to northern Australia/north-western Australia before founding the first Australian populations of *C*. *brevitarsis*; (ii) a relatively long history of Cbrev-01 in northern Australia have enabled the spread of this haplotype to the southern-most Australian limit of this species’ habitable range (Fig 1); (iii) the presence of other mtDNA haplotypes (e.g., Cbrev-03 in Kalumburu (in 2008 and 2009) and Katherine (in 2009)) suggests other possible incursion events, with members of likely source populations again being carried by trade winds from, for example, Timor-Leste or Indonesia [90]; and (iv) based on mtDNA haplotypes not present in north-western/northern Australia, at least one additional independent incursion event must have occurred, possibly from New Guinea (i.e., similar to that proposed for *C*. *dumdum*) [99] into northern QLD, which subsequently spread to our eastern sampling sites (Paterson, Grafton and Lismore).

The small number of specimens analysed from Timor-Leste and the Solomon Islands may explain the limited number of haplotypes detected in these regions. Increasing sample sizes from, for example, Timor-Leste, Papua New Guinea, Indonesia, and Solomon Islands will enable better testing of these hypotheses especially hypothesis (iv) listed above. Adding support to the proposed hypothesis of multi-incursion points into Australia of *C*. *brevitarsis* is the identification of other region-specific mtDNA haplotypes (i.e., Cbrev-04 and Cbrev-06 in the north and Cbrev-05, Cbrev-07, Cbrev-08, and Cbrev-09 in the east), although further confirmation is needed and will require extensive sampling and population genetic analysis of samples from especially northern QLD, which were not available for this study. Molecular analysis of the BTV serotypes (see [84] for a review) supports Dyce’s [97] hypothesis of a northwest-southeast spread, with an origin in the Indian subcontinent, although multiple potential *Culicoides* incursion origins (e.g., from west (e.g., Lombok) to east (Fly (Western Province, PNG) into northern Australia (WA, NT, QLD) have been reported [90, 100]. Furthermore, differences in BTV virulence patterns (Table 3 in [84]) also lend support to the proposed multiple entry points of *C*. *brevitarsis* based on the presence of disjoint northern and eastern populations, as is evident from the region-specific mtDNA haplotypes, and may be reflected by geographic clines of genetic clusters based on nuclear DNA markers.

## Conclusions

Our results showed that *C*. *brevitarsis*, with a relatively well-established range across Australia and its neighbouring countries such as Indonesia, Timor-Leste, Papua New Guinea, and Solomon Islands, may have been introduced several times into Australia, as indicated by the heterogeneous patterns of geographical distribution of the mtDNA haplotypes in northern and eastern populations. The results highlight a need for further in-depth studies of incursion pathways (e.g., significantly increase sampling sites, marker numbers, and sample over multiple seasons) of *C*. *brevitarsis* and other vectors of viruses in Australia and surrounding regions.

## Supporting Information

S1 File A multiple sequence (nucleotide) alignment (individual sequences in Fasta format) of the mtDNA COI partial gene used in this study. (PDF)

S2 File A multiple sequence (amino acids) alignment (individual sequences in Fasta format) of the mtDNA COI partial gene used in this study. (PDF)

S1 Table Uncorrected (‘p’)-pairwise mtDNA COI distances between Australian and Japanese *C*. *brevitarsis*. (DOCX)

S2 Table Uncorrected (‘p’)-pairwise mtDNA COI distances between Australian endemic *C*. *marksi*. (DOCX)

S3 Table Uncorrected (‘p’)-pairwise distances between *C*. *brevitarsis* from Australia, China, Japan, Solomon Islands and Timor-Leste based on 323bp of over-lapping gene region. Upper-triangle values are standard errors.
(DOCX)

S4 Table Single nucleotide polymorphisms (SNPs) identified through sequence alignment of the mitochondrial DNA cytochrome oxidase I (mtDNA COI) partial genes in *Culicoides brevitarsis*. SNPs in the consensus sequence were from KJ162970 (Solomon Island) and KP201844 (Cbrev-01). Unique SNPs as compared to the consensus are indicated, identical SNPs are represented by '.'; ambiguous bases A/T are indicated by 'W, and C/T as 'Y'. Start and end nucleotide positions are indicated by square boxes. The KJ162974, Cbrev-01 [KP201844] and Cbrev-05 [KP201848] are *C*. *brevitarsis* from the same location (Grafton, New South Wales) and were collected on the same date (14-Dec-07) and by the same person (H. McKenzie, New South Wales Department of Primary Industry). Grey-out regions represent non-over-lapping mtDNA COI regions between current study, [51], and [61].
(XLSX)

S5 Table Uncorrected (“p”)-distances between 25 *Culicoides* species across 554bp of partial mtDNA COI gene. (DOCX)
