# Supplementary material for: Population Genetic Structure and Potential Incursion Pathways of the Bluetongue Virus Vector Culicoides brevitarsis (Diptera: Ceratopogonidae) in Australia
Source: PLoS One. 2016 Jan 15;11(1):e0146699. doi: 10.1371/journal.pone.0146699 (PMC4714883; doi:10.1371/journal.pone.0146699)
Supplement: S1 File — (PDF) [file pone.0146699.s001.pdf]

S1 File: MtDNA COI partial sequence alignment in fasta+gap format of *Culicoides* species used in phylogenetic analysis.

```
>Cbrev-01 [GenBank:KP201844]
GCTATTTTATTATTATTATCTTTACCAGTATTAGCGGGAGCAATTACTATATTGTTAACG
GATCGAAATATTAATACTTCTTTTTTTGACCCGGCAGGAGGAGGTGATCCTATTTTATAC
CAACATTTATTTTGATTTTTTGGTCACCCAGAAGTTTATATTTTAATTTTACCAGGATTT
GGGATTGTTTCTCATATTATTTGTCAAGAAAGAGGAAAAAAAGAAGCTTTTGGAGTTTTA
GGTATAATATATGCTATTGCTGCAATTGGTTTATTAGGATTTATTGTTTGAGCTCATCAT
ATATTTACTGTTGGGTTAGATGTAGACACTCGAGCTTATTTTACTTCAGCTACTATAATT
ATTGCTGTTCCTACAGGGATTAAAATTTTTAGTTGAATAGCAACAATATATGGAACTCAA
TTAAACCTT---ACTCCTGCTTTGTTATGATCTTTAGGGTTTGTATTTTTATTTACTGTA
GGAGGATTAACAGGTATTGTTTTAGCTAATTCTTCTTTAGATATTGTTTTACATGATACA
TATTATGTA
>Cbrev-02 [GenBank:KP201845]
GCTATTTTATTATTATTATCTTTACCAGTATTAGCGGGAGCAATTACTATATTGTTAACG
GATCGAAATATTAATACTTCTTTTTTTGACCCGGCAGGAGGAGGTGATCCTATTTTATAT
CAACATTTATTTTGATTTTTTGGTCACCCAGAAGTTTATATTTTAATTTTACCAGGATTT
GGGATTGTTTCTCATATTATTTGTCAAGAAAGAGGAAAAAAAGAAGCTTTTGGAGTTTTA
GGTATAATATATGCTATTGCTGCAATTGGTTTATTAGGATTTATTGTTTGAGCTCATCAT
ATATTTACTGTTGGGTTAGATGTAGACACTCGAGCTTATTTTACTTCAGCTACTATAATT
ATTGCTGTTCCTACAGGGATTAAAATTTTTAGTTGAATAGCAACAATATATGGAACTCAA
TTAAACCTT---ACTCCTGCTTTGTTATGATCTTTAGGGTTTGTATTTTTATTTACTGTA
GGAGGATTAACAGGTATTGTTTTAGCTAATTCTTCTTTAGATATTGTTTTACATGATACA
TATTATGTA
>Cbrev-03 [GenBank:KP201846]
GCTATTTTATTATTATTATCCTTACCAGTATTAGCGGGAGCAATTACTATATTGTTAACG
GATCGAAATATTAATACTTCTTTTTTTGACCCGGCAGGAGGAGGTGATCCTATTTTATAC
CAACATTTATTTTGATTTTTTGGTCACCCAGAAGTTTATATTTTAATTTTACCAGGATTT
GGGATTGTTTCTCATATTATTTGTCAAGAAAGAGGAAAAAAAGAAGCTTTTGGAGTTTTA
GGTATAATATATGCTATTGCTGCAATTGGTTTATTAGGATTTATTGTTTGAGCTCATCAT
ATATTTACTGTTGGGTTAGATGTAGACACTCGAGCTTATTTTACTTCAGCTACTATAATT
ATTGCTGTTCCTACAGGGATTAAAATTTTTAGTTGAATAGCAACAATATATGGAACTCAA
TTAAACCTT---ACTCCTGCTTTGTTATGATCTTTAGGGTTTGTATTTTTATTTACTGTA
GGAGGATTAACAGGTATTGTTTTAGCTAATTCTTCTTTAGATATTGTTTTACATGATACA
TATTATGTA
>Cbrev-04 [GenBank:KP201847]
GCTATTTTATTATTATTATCTTTACCAGTATTAGCGGGAGCAATTACTATATTGTTAACG
GATCGAAATATTAATACTTCTTTTTTTGATCCGGCAGGAGGAGGTGATCCTATTTTATAT
CAACATTTATTTTGATTTTTTGGTCACCCAGAAGTTTATATTTTAATTTTACCAGGATTT
GGGATTGTTTCTCATATTATTTGTCAAGAAAGAGGAAAAAAAGAAGCTTTTGGAGTTTTA
GGTATAATATATGCTATTGCTGCAATTGGTTTATTAGGATTTATTGTTTGAGCTCATCAT
ATATTTACTGTTGGGTTAGATGTAGACACTCGAGCTTATTTTACTTCAGCTACTATAATT
ATTGCTGTTCCTACAGGGATTAAAATTTTTAGTTGAATAGCAACAATATATGGAACTCAA
TTAAACCTT---ACTCCTGCTTTGTTATGATCTTTAGGGTTTGTATTTTTATTTACTGTA
GGAGGATTAACAGGTATTGTTTTAGCTAATTCTTCTTTAGATATTGTTTTACATGATACA
TATTATGTA
>Cbrev-05 [GenBank:KP201849]
GCTATTTTATTATTATTATCTTTACCAGTATTAGCGGGAGCAATTACTATATTGTTAACG
GATCGAAATATTAATACTTCTTTTTTTGACCCGGCAGGAGGAGGTGATCCTATTTTATAC
CAACATTTATTTTGATTTTTTGGTCACCCAGAAGTTTATATTTTAATTTTACCAGGATTT
GGGATTGTTTCTCATATTATTTGTCAAGAAAGAGGAAAAAAAGAAGCTTTTGGAGTTTTA
GGTATAATATATGCTATTGCTGCAATTGGTTTATTAGGATTTATTGTTTGAGCTCATCAT
ATATTTACTGTTGGGTTAGATGTAGACACTCGAGCTTATTTTACTTCAGCTACTATAATT
ATTGCTGTTCCTACAGGGATTAAAATTTTTAGTTGAATAGCAACAATATATGGAACTCAA
TTAAACCTT---ACTCCTGCTTTGTTATGATCTTTAGGGTTTGTATTTTTATTTACTGTA
GGAGGATTAACAGGTATTGTTTTAGCTAATTCTTCTTTAGATATTGTTTTACATGATACA
TATTATGTA
>Cbrev-06 [GenBank:KP201849]
GCTATTTTATTATTATTATCTTTACCAGTATTAGCGGGAGCAATTACTATATTGTTAACG
GATCGAAATATTAATACTTCTTTTTTTGACCCGGCAGGAGGAGGTGATCCTATTTTATAT
CAACATTTATTTTGWTTTTTTGGTCACCCAGAAGTTTATATTTTAATTTTACCAGGATTT
```

GGGATTGTTTCTCATATTATTTGTCAAGAAAGAGGAAAAAAAGAAGCTTTTGGAGTTTTA
GGTATAATATATGCTATTGCTGCAATTGGTTTATTAGGATTTATTGTTTGAGCTCATCAT
ATATTTACTGTTGGGTTAGATGTAGACACTCGAGCTTATTTTACTTCAGCTACTATAATT
ATTGCTGTTCCTACAGGGATTAAAATTTTTAGTTGAATAGCAACAATATATGGAACTCAA
TTAAACCTT---ACTCCTGCTTTGTTATGATCTTTAGGGTTTGTATTTTTATTTACTGTA
GGAGGATTAACAGGTATTGTTTTAGCTAATTCTTCTTTAGATATTGTTTTACATGACACA
TATTATGTA
>Cbrev-07 [GenBank:KP201850]
GCTATTTTATTATTATTATCTTTACCAGTATTAGCGGGAGCAATTACTATATTGTTAACG
GATCGAAATATTAATACTTCTTTTTTTGATCCGGCAGGAGGAGGTGATCCTATTTTATAT
CAACATTTATTTTGATTTTTTGGTCACCCAGAAGTTTATATTTTAATTTTACCAGGATTT
GGAATTGTTTCTCATATTATTTGTCAAGAAAGAGGAAAAAAAGAAGCTTTTGGAGTTTTA
GGTATAATATATGCTATTGCTGCAATTGGTTTATTAGGATTTATTGTTTGAGCTCATCAT
ATATTTACTGTTGGGTTAGATGTAGACACTCGAGCTTATTTTACTTCAGCTACTATAATT
ATTGCTGTTCCTACAGGGATTAAAATTTTTAGTTGAATAGCAACAATATATGGAACTCAA
TTAAACCTT---ACTCCTGCTTTGTTATGATCTTTAGGGTTTGTATTTTTATTTACTGTA
GGAGGATTAACAGGTATTGTTTTAGCTAATTCTTCTTTAGATATTGTTTTACATGATACA
TATTATGTA
>Cbrev-08 [GenBank:KP201851]
GCTATTTTATTATTATTATCTTTACCAGTATTAGCGGGAGCAATTACTATATTGTTAACG
GATCGAAATATTAATACTTCTTTTTTTGACCCGGCAGGAGGAGGTGATCCTATTTTATAT
CAACATTTATTTTGATTTTTTGGTCACCCAGAAGTTTATATTTTAATTTTACCAGGATTT
GGGATTGTTTCTCATATTATTTGTCAAGAAAGAGGAAAAAAAGAAGCTTTTGGAGTTTTA
GGTATAATATATGCTATTGCTGCAATTGGTTTATTAGGATTTATTGTTTGAGCTCATCAT
ATATTTACTGTTGGGTTAGATGTAGACACTCGAGCTTATTTTACTTCAGCTACTATAATT
ATTGCTGTTCCTACAGGGATTAAAATTTTTAGTTGAATAGCAACAATATATGGAACTCAA
TTAAACCTT---ACTCCTGCTTTGTTATGATCTTTAGGGTTTGTATTTTTATTTACTGTA
GGAGGATTAACAGGTATTGTTTTAGCTAATTCTTCTTTAGATATTGTTTTACATGATACA
TATTATGGA
>Cbrev-09 [GenBank:KP201852]
GCTATTTTATTATTATTATCTTTACCAGTATTAGCGGGAGCAATTACTATATTGTTAACG
GATCGAAATATTAATACTTCTTTTTTTGACCCGGCAGGAGGAGGTGATCCTATTTTATAT
CAACATTTATTTTGATTTTTTGGTCACCCAGAAGTTTATATTTTAATTTTACCAGGATTT
GGGATTGTTTCTCATATTATTTGTCAAGAAAGAGGAAAAAAAGAAGCTTTTGGAGTTTTA
GGTATAATATATGCTATTGCTGCAATTGGTTTATTAGGATTTATTGTTTGAGCTCATCAT
ATATTTACTGTTGGGTTAGATGTAGACACTCGAGCTTATTTTACTTCAGCTACTATAATT
ATTGCTGTTCCTACAGGGATTAAAATTTTTAGTTGAATAGCAACAATATATGGAACTCAA
TTAAATCTT---ACTCCTGCTTTGTTATGATCTTTAGGGTTTGTATTTTTATTTACTGTA
GGAGGATTAACAGGTATTGTTTTAGCTAATTCTTCTTTAGATATTGTTTTACATGATACA
TATTATGTA
>Cmarksi-01 [GenBank:KP201853]
GCCATTTTATTATTTTTATCTTTACCTGTTTTAGCTGGAGCTATTACTATACTTTTAACT
GACCGTAATATTAATACTTCATTTTTTGACCCAGCTGGAGGAGGAGACCCTATTTTATAC
CAGCATTTATTTTGATTTTTTGGACACCCAGAAGTTTATATTTTAATTTTACCAGGATTT
GGAATAATTTCTCATATTATTAGTCAAGAAAGTGGGAAAAAGGAAACCTTTGGGTCACTT
GGAATAATTTATGCCATACTAGCAATTGGACTTTTAGGATTTATTGTATGAGCCCACCAT
ATATTTACTGTAGGAATAGACGTAGACACCCGAGCTTACTTTACTTCTGCTACAATAATT
ATTGCTGTTCCTACGGGGATTAAAATTTTTAGTTGATTAGCCACTCTTCATGGAACACAA
ATAACGCTT---AATCCATCTTTATTATGAGCTTTAGGGTTTGTATTCTTATTCACAGTA
GGAGGATTAACTGGAGTTATTTTAGCAAATTCTTCTATTGATATTGTATTACATGACACT
TATTATGTA
>Cmarksi-03 [GenBank:KP201854]
GCCATTTTATTATTTTTATCTTTACCTGTTTTAGCTGGAGCTATTACTATACTTTTAACT
GACCGTAATATTAATACTTCATTTTTTGACCCAGCTGGAGGAGGAGACCCTATTTTATAC
CAGCATTTATTTTGATTTTTTGGACATCCAGAAGTTTATATTTTAATTTTACCAGGATTT
GGGATAATTTCTCATATTATTAGCCAAGAAAGTGGAAAAAAGGAAACCTTTGGTTCACTT
GGAATAATTTATGCCATACTAGCAATTGGACTTTTAGGATTTATTGTATGAGCCCACCAT
ATATTTACTGTAGGAATAGACGTAGACACCCGAGCTTACTTTACTTCTGCTACAATAATT
ATTGCTGTTCCTACGGGGATTAAAATTTTTAGTTGATTAGCCACTCTTCATGGAACACAA
ATAACGCTT---AATCCATCTTTATTATGAGCTTTAGGGTTTGTATTCTTATTCACAGTA
GGAGGATTAACTGGGGTTATTTTAGCAAATTCTTCTATTGATATTGTATTACATGACACT
TATTATGTA
>Cmarksi-4 [GenBank:KP201855]

GCCATTTTATTATTTTTATCTTTACCTGTTTTAGCTGGAGCTATTACTATACTTTTAACT
GACCGTAACATTAATACTTCATTTTTTGACCCAGCTGGAGGGGGGGACCCTATTTTATAC
CAGCATTTATTTTGATTTTTTGGACATCCAGAAGTTTATATTTTAATTTTACCAGGATTT
GGAATAATTTCTCATATTATTAGCCAAGAAAGTGGAAAAAAGGAAACCTTTGGGTCACTT
GGAATAATTTATGCTATACTAGCAATTGGACTTTTAGGATTTATTGTATGAGCCCMCCAT
ATATTTACTGTAGGAATAGACGTAGACACCCGAGCTTACTTTACTTCTGCTACAATAATT
ATTGCTGTTCCTACGGGGATTAAAATTTTTAGTTGACTAGCCACTCTTCATGGAACACAA
ATAACGCTT---AATCCATCTTTATTATGAGCTTTAGGATTTGTATTCTTATTCACAGTA
GGAGGATTAACTGGAGTTATCTTAGCAAATTCTTCTATTGATATCGTATTACATGACACT
TATTATGTA
>Cmarksi-05 [GenBank:KP201856]
GCCATTTTATTATTTTTATCTTTACCTGTTTTAGCGGGAGCTATTACTATACTTTTAACT
GACCGTAATATTAATACTTCATTTTTTGACCCAGCTGGAGGAGGAGACCCTATTTTATAC
CAGCATTTATTTTGATTTTTTGGGCATCCAGAAGTTTATATTTTAATTTTACCAGGATTT
GGGATAATTTCTCATATTATTAGCCAAGAAAGTGGAAAAAAGGAAACCTTTGGTTCACTT
GGAATAATTTATGCCATACTAGCAATTGGACTTTTAGGATTTATTGTATGAGCCCMCCAT
ATATTTACTGTAGGAATAGACGTAGACACCCGAGCTTACTTTACTTCAGCTACAATAATT
ATTGCTGTTCCTACGGGGATTAAAATTTTTAGTTGATTAGCCACTCTTCATGGAACACAA
ATAACGCTT---AATCCATCTTTATTATGAGCTTTAGGGTTTGTATTCTTATTCACAGTA
GGAGGATTAACTGGGGTTATTTTAGCAAATTCTTCTATTGATATTGTATTACATGACACT
TATTATGTA
>Cmarksi-06 [GenBank:KP201857]
GCCATTTTATTATTTTTATCTTTACCTGTTTTAGCTGGAGCTATTACTATACTTTTAACT
GACCGTAATATTAATACTTCATTTTTTGACCCAGCTGGAGGAGGAGACCCTATTTTATAC
CAGCATTTATTTTGATTTTTTGGGCATCCAGAAGTTTATATTTTAATTTTACCAGGATTT
GGGATAATTTCTCATATTATTAGCCAAGAAAGTGGAAAAAAGGAAACCTTTGGTTCACTT
GGAATAATTTATGCCATACTAGCAATTGGACTTTTAGGATTTATTGTATGAGCCCACCAT
ATATTTACTGTAGGAATAGACGTAGACACCCGAGCTTACTTTACTTCTGCTACAATAATT
ATTGCTGTCCCTACGGGGATTAAAATTTTTAGTTGATTAGCCACTCTTCATGGAACACAA
ATAACGCTT---AATCCATCTTTATTATGAGCTTTAGGGTTTGTATTCTTATTCACAGTA
GGAGGATTAACTGGGGTTATTTTAGCAAATTCTTCTATTGATATTGTATTACATGACACT
TATTATGTA
>Cmarksi-07 [GenBank:KP201858]
GCTATTTTATTATTTTTATCTTTACCTGTTTTAGCTGGAGCTATTACTATACTTTTAACT
GACCGTAACATTAATACTTCATTTTTTGACCCAGCTGGAGGGGGGGACCCTATTTTATAC
CAGCATTTATTTTGATTTTTTGGACATCCAGAAGTTTATATTTTAATTTTACCAGGATTT
GGAATAATTTCTCATATTATTAGCCAAGAAAGTGGAAAAAAGGAAACCTTTGGGTCACTT
GGAATAATTTATGCTATACTAGCAATTGGACTTTTAGGATTTATTGTATGAGCCCMCCAT
ATATTTACTGTAGGAATAGACGTAGACACCCGAGCTTACTTTACTTCTGCTACAATAATT
ATTGCTGTTCCTACGGGGATTAAAATTTTTAGTTGACTAGCCACTCTTCATGGAACACAA
ATAACGCTT---AATCCATCTTTATTATGAGCTTTAGGATTTGTATTCTTATTCACAGTA
GGAGGATTAACTGGAGTTATCTTAGCAAATTCTTCTATTGATATCGTATTACATGACACT
TATTATGTA
>Cmarksi-08 [GenBank:KP201859]
GCCATTTTATTATTTTTATCTTTACCTGTTTTAGCTGGAGCTATTACTATACTTTTAACT
GACCGTAACATTAATACTTCATTTTTTGACCCAGCTGGAGGGGGGGACCCTATTTTATAC
CAGCATTTATTTTGATTTTTTGGACATCCAGAAGTTTATATTTTAATTTTACCAGGATTT
GGAATAATTTCTCATATTATTAGCCAAGAAAGTGGAAAAAAGGAAACCTTTGGGTCACTT
GGAATAATTTATGCTATACTAGCAATTGGACTTTTAGGATTTATTGTATGAGCCCMCCAT
ATATTTACTGTAGGAATAGACGTAGACACCCGAGCTTACTTTACTTCTGCTACAATAATT
ATTGCTGTTCCTACGGGGATTAAAATTTTTAGTTGATTAGCCACTCTTCATGGACACAA
ATAACGCTT---AATCCATCTTTATTATGAGCTTTAGGATTTGTATTCTTATTCACAGTA
GGAGGATTAACTGGAGTTATCTTAGCAAATTCTTCTATTGATATCGTATTACATGACACT
TATTATGTA
>Cmarksi-09 [GenBank:KP201860]
GCCATTTTATTATTTTTATCTTTACCTGTTTTAGCTGGAGCTATTACTATACTTTTAACT
GACCGTAATATTAATACTTCATTTTTTGACCCAGCTGGAGGAGGAGACCCTATTTTATAC
CAGCATTTATTTTGATTTTTTGGACACCCAGAAGTTTATATTTTAATTTTACCAGGATTT
GGAATAATTTCTCATATTATTAGTCAAGAAAGTGGGAAAAAAGAAACCTTTGGGTCACTT
GGAATAATTTATGCCATACTAGCAATTGGACTTTTAGGATTTATTGTATGAGCCCMCCAT
ATATTTACTGTAGGAATAGACGTAGACACCCGAGCTTACTTTACTTCTGCTACAATAATT
ATTGCTGTTCCTACGGGGATTAAAATTTTTAGTTGATTAGCCACTCTTCATGGAACACAA
ATAACGCTT---AATCCATCTTTATTATGAGCTTTAGGGTTTGTATTCTTATTCACAGTA

GGAGGATTAACTGGAGTTATTTTAGCAAATTCTTCTATTGATATTGTATTACATGACACT
TATTATGTA
>Cmarksi-10 [GenBank:KP201861]
GCCATTTTATTATTTTTATCTTTACCTGTTTTAGCTGGAGCTATTACTATACTTTTAACT
GACCGTAACATTAATACTTCATTTTTTGACCCAGCTGGAGGGGGAGACCCTATTTTATAC
CAACATTTATTTTGATTTTTTGGACATCCAGAAGTTTATATTTTAATTTTACCAGGATTT
GGGATAATTTCTCATATTATTAGCCAAGAAAGTGGAAAAAAGGAAACCTTTGGGTCACTT
GGAATAATTTATGCTATACTAGCAATTGGACTTTTAGGATTTATTGTATGGGCCCACCAT
ATATTTACTGTAGGAATAGACGTGGACACCCGAGCTTACTTTACTTCTGCTACAATAATT
ATTGCTGTTCCTACGGGAATTAAAATTTTTAGTTGATTAGCCACTCTTCATGGAACACAA
ATAACGCTT---AATCCATCTTTATTATGAGCTTTAGGATTTGTATTCTTATTCACAGTA
GGAGGGTTAACTGGAGTTATTTTAGCAAATTCTTCTATTGATATCGTATTACATGACACT
TATTATGTA
>Cmarksi-11 [GenBank:KP201862]
GCCATTTTATTATTTTTATCTTTACCTGTTTTAGCTGGGGCTATTACTATACTTTTAACT
GATCGTAACATTAATACTTCATTTTTTGACCCAGCTGGAGGGGGAGACCCTATTTTATAC
CAGCATTTATTTTGATTTTTTGGGCATCCAGAAGTTTATATTTTAATTTTACCAGGATTT
GGAATAATTTCTCATATTATTAGCCAAGAAAGTGGAAAAAAGGAAACCTTTGGGTCACTT
GGAATAATTTATGCTATGCTAGCAATTGGACTTTTAGGATTTATTGTATGAGCCCACCAT
ATATTTACTGTAGGAATAGACGTAGACACCCGAGCTTACTTTACTTCTGCTACAATAATT
ATTGCTGTTCCTACGGGGATTAAAATTTTTAGTTGATTGGCCACTCTTCATGGAACACAA
ATAACACTT---AATCCATCTTTATTATGAGCTTTAGGATTTGTATTCTTATTCACAGTA
GGAGGATTAACTGGAGTTATTTTAGCAAATTCTTCTATTGATATCGTATTACATGACACT
TATTATGTA
>Cmarksi-12 [GenBank:KP201863]
GCCATTTTATTATTTTTATCTTTACCTGTTTTAGCCGGAGCTATTACTATACTTTTAACT
GACCGTAACATTAATACTTCATTTTTTGACCCAGCTGGAGGGGGAGACCCTATTTTATAC
CAGCATTTATTTTGATTTTTTGGACATCCAGAAGTTTATATTTTAATTTTACCAGGATTT
GGGATAATTTCTCATATTATTAGCCAAGAAAGTGGAAAAAAGGAAACCTTTGGGTCACTT
GGAATAATTTATGCTATACTAGCAATTGGACTTTTAGGATTTATTGTATGAGCCCACCAT
ATATTTACTGTAGGAATAGACGTAGACACCCGAGCTTACTTTACTTCTGCTACAATAATT
ATTGCTGTTCCTACGGGGATTAAAATTTTTAGTTGATTAGCCACTCTTCATGGAACACAA
ATAACGCTT---AATCCATCTTTATTATGAGCTTTAGGATTTGTATTCTTATTTACAGTA
GGAGGATTAACTGGAGTTATTTTAGCAAATTCTTCTATTGATATCGTATTACATGACACT
TATTATGTA
>Cmarksi-13 [GenBank:KP201864]
GCCATTTTATTATTTTTATCTTTACCTGTTTTAGCTGGAGCTATTACTATACTTTTAACT
GACCGTAATATTAATACTTCATTTTTTGACCCAGCTGGAGGAGGAGACCCTATTTTATAC
CAGCATTTATTTTGATTTTTTGGACATCCAGAAGTTTATATTTTAATTTTACCAGGATTT
GGAATAATTTCTCATATTATTAGCCAAGAAAGTGGAAAAAAGGAAACTTTTGGGTCACTT
GGAATAATTTATGCCATACTAGCAATTGGACTTTTAGGATTTATTGTATGAGCCCMCCAT
ATATTTACTGTAGGAATAGACGTAGACACCCGAGCTTACTTTACTTCTGCTACAATAATT
ATTGCTGTTCCTACGGGGATTAAAATTTTTAGTTGATTAGCCACTCTTCATGGAACACAA
ATAACGCTT---AATCCATCTTTATTATGAGCTTTAGGGTTTGTATTTTTATTCACAGTA
GGAGGATTAACTGGAGTTATTTTAGCAAATTCTTCTATTGATATTGTATTACATGACACT
TATTATGTA
>Cmarksi-14 [GenBank:KP201865]
GCTATTTTATTATTTTTATCTTTACCTGTTTTAGCTGGAGCTATTACTATACTTTTAACT
GACCGTAATATTAATACTTCATTTTTTGACCCAGCTGGAGGAGGAGACCCTATTTTATAC
CAGCATTTATTTTGATTTTTTGGACACCCAGAAGTTTATATTTTAATTTTACCAGGATTT
GGAATAATTTCTCATATTATTAGTCAAGAAAGTGGGAAAAAGGAAACCTTTGGGTCACTT
GGAATAATTTATGCCATACTAGCAATTGGACTTTTAGGATTTATTGTATGAGCCCACCAT
ATATTTACTGTAGGAATAGACGTAGACACCCGAGCTTACTTTACTTCTGCTACAATAATT
ATTGCTGTTCCTACGGGGATTAAAATTTTTAGTTGATTAGCCACTCTTCATGGAACACAA
ATAACGCTT---AATCCATCTTTATTATGAGCTTTAGGGTTTGTATTCTTATTCACAGTA
GGAGGATTAACTGGAGTTATTTTAGCAAATTCTTCTATTGATATTGTATTACATGACACT
TATTATGTA
>Cmarksi-15 [GenBank:KP201866]
GCTATTTTATTATTTTTATCTTTACCTGTTTTAGCTGGAGCTATTACTATACTTTTAACT
GACCGTAACATTAATACTTCATTTTTTGACCCAGCTGGAGGGGGAGACCCTATTTTATAC
CAGCATTTATTTTGATTTTTTGGACATCCAGAAGTTTATATTTTAATTTTACCAGGATTT
GGGATAATTTCTCATATTATTAGCCAAGAAAGTGGAAAAAAGGAAACCTTTGGGTCACTT
GGAATAATTTATGCTATACTAGCAATTGGACTTTTAGGATTTATTGTATGAGCCCACCAT

```
ATATTTACTGTAGGAATAGACGTAGACACCCGAGCTTACTTTACTTCTGCTACAATAATT
ATTGCTGTTCCTACGGGGATTAAAATTTTTAGTTGATTAGCCACTCTTCATGGAACACAA
ATAACGCTT---AATCCATCTTTATTATGAGCTTTAGGATTTGTATTCTTATTTACAGTA
GGAGGATTAACTGGAGTTATTTTAGCAAATTCTTCTATTGATATCGTATTACATGACACT
TATTATGTA
>Cmarksi-16 [GenBank:KP201867]
GCCATTTTATTATTTTTATCTTTACCTGTTTTAGCTGGAGCTATTACTATACTTTTAACT
GACCGTAATATTAATACTTCATTTTTTGACCCAGCTGGAGGAGGAGACCCTATTTTATAC
CAGCATTTATTTTGATTTTTTGGGCATCCAGAAGTTTATATTTTAATTTTACCAGGATTT
GGAATAATTTCTCATATTATTAGCCAAGAAAGTGGAAAAAAGGAAACTTTTGGGTCACTT
GGAATAATTTATGCCATACTAGCAATTGGACTTTTAGGATTTATTGTATGAGCCCACCAT
ATATTTACTGTAGGAATAGACGTAGACACCCGAGCTTACTTTACTTCTGCTACAATAATT
ATTGCTGTTCCTACGGGGATTAAAATTTTTAGTTGATTAGCCACTCTTCATGGAACACAA
ATAACGCTT---AATCCATCTTTATTATGAGCTTTAGGGTTTGTATTTTTMTTCACAGTA
GGAGGATTAACTGGAGTTATTTTAGCAAATTCTTCTATTGATATTGTATTACATGACACT
TATTATGTA
>Cpallid-221 [GenBank:KP201868]
GCCATTTTATTATTATTATCTTTACCTGTATTAGCCGGGGCAATTACGATACTTTTAACC
GACCGTAATATTAATACATCCTTTTTTGACCCAGCTGGCGGAGGAGACCCAATTTTATAC
CAACATTTATTTTGATTTTTTGGACATCCAGAAGTATATATTTTAATTCTTCCCGGATTT
GGAATAATTTCTCATATTATTAGCCAAGAAAGAGGTAAAAAAGAAACTTTTGGATCATTA
GGAATAATTTATGCAATACTTGCTATTGGATTATTAGGATTTATCGTTTGAGCCCACCAT
ATATTTACAGTTGGAATAGATGTTGACACTCGAGCTTATTTTACTTCTGCTACTATAATT
ATTGCCGTACCTACAGGAATTAAAATTTTTAGATGATTAGCAACTCTTCACGGCACCCAA
ATAACTTTA---AATCCTTCTCTTTTATGAGCTTTAGGCTTTGTATTTTTATTTACTGTT
GGAGGACTAACTGGTGTAATTTTAGCCAATTCTTCTATTGATATTGTTCTTCATGATACT
TATTACGTA
>Chenryi-02 [GenBank:KP201869]
GCCATTTTATTATTATTATCTTTACCAGTATTAGCTGGGGCTATTACCATATTATTAACA
GACCGAAATATTAATACTTCTTTTTTTGACCCAGCTGGGGGGGGGGACCCTATTTTATAC
CAACATTTATTTTGATTTTTTGGCCACCCAGAAGTTTATATTTTAATTTTACCCGGATTT
GGTATAATTTCTCACATTATTAGCCAAGAAAGAGGAAAAAAAGAAACCTTTGGGTCTCTG
GAAATAATTTATGCTATGCTAGCTATTGGGCTGTTAGGATTTATTGTTTGAGCCCACCAT
ATATTTACAGTCGGAATGGATGTCGACACACGAGCTTATTTTACTTCTGCTACAATAATT
ATTGCTGTACCAACAGGAATTAAAATTTTTAGCTGATTAGCAACTCTTCATGGCACACAA
ATAACTTTA---AACCCCTCTCTTTTATGAGCCTTAGAATGGGTATTTTTATTTACAGTT
GGGGGATTAACCGGGGTTATTTTAGCTAATTCATCTNNNNNNNNNNNNNNNNNNNNNNNN
NNNNNNNNN
>Chenryi-01 [GenBank:KP201870]
GCTATTTTATTATTATTATCTTTACCAGTATTAGCTGGGGCTATTACCATATTATTAACA
GACCGAAATATTAATACTTCTTTTTTTGACCCAGCTGGGGGAGGAGACCCTATTTTATAC
CAACATTTATTTTGATTTTTTGGCCACCCAGAAGTTTATATTTTAATTTTACCTGGATTT
GGTATAATTTCTCACATTATTAGCCAAGAAAGAGGAAAAAAAGAAACCTTTGGGTCTCTG
GGAATAATTTATGCTATACTAGCTATTGGGTTGTTAGGATTTATTGTTTGAGCCCACCAT
ATATTTACAGTCGGAATGGATGTCGACACACGAGCTTATTTTACTTCTGCTACAATAATT
ATTGCTGTACCAACAGGAATTAAAATTTTTAGCTGATTAGCGACTCTTCATGGCACACAA
ATAACTTTA---AACCCCTCTCTTTTATGAGCCTTAGGATTTGTATTTTTATTTACAGTT
GGGGGATTAACCGGGGTTATTTTAGCTAATTCATCTATTGATATTGTCCTCCATGACACA
TATTATGTA
>Cbundye-01 [GenBank:KP201871]
NNNNNNNNNNNNNNNNNNNNNNNNNNNNNNNNNNNNNNNNNNNNNNNNNNNNNNNTTRACK
GATCGAAATATTAATACTTCTTTTTTTGACCCTGCCGGAGGAGGAGATTCAATCCTATAC
CAACATCTATTCTGATTTTTTGGACACCCTGAAGTTTATATTTTAATTTTGCCCGGATTT
GGTATAGTTTCACATATTATTAGCCAAGAAAGAGGAAAAAAAGAAACTTTTGGATCTTTA
GGGATAATTTATGCTATACTTGCTATTGGTTTATTAGGATTCATTGTTTGAGCCCACCAT
ATATTTACAGTAGGGATAGATGTTGACACTCGAGCTTATTTTACTTCAGCTACAATAATT
ATTGCTGTCCCAACAGGAATTAAAATTTTTAGTTGATTGGCCACTCTTCATGGAACCCAA
ATAACACCT---AATGCCTCTTTATTATGAGCCCTAGGATTTGTATTTTTATTTACTGTA
GGGAGACTGACAGGAGTAATTTTGGCTAATTCATCTATTRATATTGGTTTACATGATACA
TATTATGTA
>Cbunroo-01 [GenBank:KP201872]
GCCATTTTATTAYTATTATCTTTACCAGTCTTAGCCGGAGCTATCACTATATTATTAACA
GACCGTAATATTAATACTTCATTTTTTGACCCCGCCGGAGGAGGCGACCCAATTCTTTAT
```

CAACATTTATTTTGATTTTTTGGGCACCCAGAAGTTTATATTTTAATTTTACCTGGATTT
GGAATAATTTCTCATATTATTAGCCAAGAAAGAGGTAAAAAAGAAACTTTTGGATCTTTA
GGAATAATCTATGCAATACTAGCAATTGGACTTTTAGGATTTATTGTATGAGCTCATCAT
ATATTTACTGTAGGAATAGATGTAGATACTCGTGCTTACTTTACTTCAGCTACTATAATT
ATTGCTGTGCCAACAGGAATTAAAATTTTTAGCTGATTAGCAACATTACATGGGACACAA
ATAACATTA---AATCCCTCTCTTCTTTGATCTCTAGGATTTGTATTCCTATTTACAGTA
GGGGGACTCACAGGAGTAATCTTAGCTAATTCTTCCATTGATATTGTTCTTCATGATACC
TACTATGTA
>C.actoni JAP [GenBank:AB360971]
GCATTATTATTATTACTCTCTTTACCTGTATTAGCAGGAGCTATTACTATGCTTCTTACC
GATCGTAATTTTAATACTTCATTTTTTGACCCTGCAGGAGGAGGAGACCCTGTTCTTTAT
CAACATTTATTTTGATTTTTTGGTCATCCAGAGGTTTATATTTTAATTTTACCTGCTTTT
GGGATTATTTCACACATTATGGCTGGAGAAAGTGGGAAAAAGGAACCATTTGGAGTTTTA
GCCATACAATACGCAATTAGAGCTATTGGGTTATTAGGATTTTTTGTGTGAGCACATCAT
ATGTTTACAGTTGGGTTAGATGTAGATACTCGAGCCTACTTCTCTGCAGCTACAATAGTA
ATTGGAATTCCCACAGGAATTAAAATTTTTAGTTGATTAGCTACTATTTATGGAAGTCCA
TGACAATTT---ACTCCAGCTATATTATGAGCTTTAGGATTTATTTTTTTATTTACTTTA
GGGGGATTAACTGGAATTGTTTTATCAAATACTGCTATTGATATTGTATTACATGATACT
TATTATGTT
>C.arakawae JAP 1 [GenBank:AB360972]
GCTATTCTTTTACTTCTTTCTTTACCTGTATTAGCTGGAGCTATTACTATATTATTAACC
GATCGAAATATTAATACTTCTTTTTTTGACCCCGCCGGTGGGGGAGACCCAATTTTATAC
CAACATTTATTTTGATTTTTTGGCCACCCCGAAGTTTATATTTTAATTTTACCAGGATTC
GGAATAATCTCTCATATTATCAGCCAAGAAAGAGGGAAAAAAGAAACTTTTGGAGCTTTA
GGAATAATTTACGCTATACTTGCTATTGGATTATTAGGATTCATTGTTTGAGCTCACCAC
ATATTTACAGTAGGAATAGACGTTGATACTCGAGCATATTTTACTTCCGCTACTATAATT
ATTGCGGTTCCTACTGGAATTAAAATTTTTAGATGATTGGCCACCCTTCATGGAACACAA
ATAACTTTA---ACCCCTTCTCTATTATGATCCTTAGGATTTGTATTTTTATTTACTGTA
GGAGGATTAACAGGAGTTATCTTAGCAAACTCCTCTATTGATATTATCCTTCATGACACT
TATTATGTC
>C.arakawae JAP 2 [GenBank:AB360973]
GCTATTCTTTTACTTCTTTCTTTACCTGTATTAGCTGGAGCTATTACTATATTATTAACC
GATCGAAATATTAATACTTCTTTTTTTGACCCCGCCGGTGGGGGAGACCCAATTTTATAC
CAACATTTATTTTGATTTTTTGGCCACCCCGAAGTTTATATTTTAATTTTACCAGGATTC
GGAATAATCTCTCATATTATCAGTCAAGAAAGAGGGAAAAAAGAAACTTTTGGAGCTTTA
GGAATAATTTACGCTATACTTGCTATTGGATTATTAGGATTCATTGTTTGAGCTCACCAC
ATATTTACAGTAGGAATAGACGTTGATACTCGAGCATATTTTACTTCCGCTACTATAATT
ATTGCGGTTCCTACTGGAATTAAAATTTTTAGATGATTGGCCACCCTTCATGGAACACAA
ATAACTTTA---ACCCCTTCTCTATTATGATCCTTAGGATTTGTATTTTTATTTACTGTA
GGAGGATTAACAGGAGTTATCTTAGCAAACTCCTCTATTGATATTATCCTTCATGACACT
TATTATGTC
>C.arakawae JAP3 [GenBank:AB360974]
GCTATTCTTTTACTTCTTTCTTTACCTGTATTAGCTGGAGCTATTACTATATTATTAACC
GATCGAAATATTAATACTTCTTTTTTTGACCCCGCTGGGGGGGGAGACCCAATTTTATAC
CAACATTTATTTTGATTTTTTGGCCACCCCGAAGTTTATATTTTAATTTTACCAGGATTC
GGAATAATCTCTCATATTATCAGTCAAGAAAGAGGGAAAAAAGAAACTTTTGGAGCTTTA
GGAATAATTTACGCTATACTTGCTATTGGATTATTAGGATTCATTGTTTGAGCTCACCAC
ATATTTACAGTAGGAATAGACGTTGATACTCGAGCATATTTTACTTCCGCTACTATAATT
ATTGCGGTTCCTACTGGAATTAAAATTTTTAGATGATTGGCCACCCTTCATGGAACACAA
ATAACTTTA---ACCCCTTCTCTATTATGATCCTTAGGATTTGTATTTTTATTTACTGTA
GGAGGATTAACAGGAGTTATCTTAGCAAACTCCTCTATTGATATTATCCTTCATGACACT
TATTATGTC
>C.arakawae JAP4 [GenBank:AB360975]
GCTATTCTTTTACTTCTTTCTTTACCTGTATTAGCTGGAGCTATTACTATATTATTAACC
GATCGAAATATTAATACTTCTTTTTTTGACCCCGCCGGTGGGGGAGACCCAATTTTATAC
CAACATTTATTTTGATTTTTTGGCCACCCCGAAGTTTATATTTTAATTTTACCAGGATTC
GGAATAATCTCTCATATTATCAGTCAAGAAAGAGGGAAAAAAGAAACTTTTGGAGCTTTA
GGAATAATTTACGCTATACTTGCTATTGGATTATTAGGATTCATTGTTTGAGCTCACCAC
ATATTTACAGTAGGAATAGACGTTGATACTCGAGCATATTTTACTTCCGCTACTATAATT
ATTGCGGTTCCTACCGGAATTAAAATTTTTAGATGATTGGCCACCCTTCATGGAACACAA
ATAACTTTA---ACCCCTTCTCTATTATGATCCTTAGGATTTGTATTTTTATTTACTGTA
GGAGGATTAACAGGAGTTATCTTAGCAAACTCCTCTATTGATATTATCCTTCATGACACT
TATTATGTC

>C.arakawae JAP5 [GenBank:AB361004-Yng05_2
GCTATTCTTTTACTTCTTTCTTTACCTGTATTAGCTGGAGCTATTACTATATTATTAACC
GATCGAAATATTAATACTTCTTTTTTTGACCCCGCCGGTGGGGGAGACCCAATTTTATAC
CAACATTTATTTTGATTTTTTGGCCACCCCGAAGTTTATATTTTAATTTTACCAGGATTC
GGAATAATCTCTCATATTATCAGTCAAGAAAGAGGGAAAAAAGAAACTTTTGGAGCTTTA
GGAATAATTTACGCTATACTTGCTATTGGATTATTAGGATTCATTGTTTGAGCTCACCAC
ATATTTACAGTAGGAATAGACGTTGATACTCGAGCATATTTTACTTCCGCTACTATAATT
ATTGCGGTTCCTACTGGAATTAAAATTTTTAGATGATTGGCCACCCTTCATGGAACACAA
ATAACTTTA---ACCCCTTCTCTATTATGATCCTTAGGATTTGTATTTTTATTTACTGTA
GGAGGATTAACAGGAGTTATCTTAGCAAACTCCTCTATTGATATTATCCTTCATGACACT
TATTATGTC
>C.verbosus [GenBank:AB646615]
GCCATTCTCCTTCTCCTATCTCTACCTGTATTAGCCGGAGCTATTACTATATTATTAACT
GATCGAAATATTAATACTTCATTTTTTGACCCAGCTGGCGGGGGAGACCCCATCTTATAC
CAACATTTATTTTGATTCTTTGGACACCCAGAAGTTTATATTTTAATTTTACCAGGATTC
GGGATAATCTCCCATATTATTAGTCAAGAAAGCGGTAAAAAAGAAACCTTTGGTTCTTTA
GGAATAATTTACGCTATACTAGCTATTGGATTATTAGGATTTATTGTTTGAGCCCATCAT
ATATTTACTGTAGGAATAGATGTAGATACCCGGGCTTACTTTACTTCAGCTACCATAATT
ATTGCGGTTCCAACAGGAATTAAAATTTTTAGTTGACTGGCCACTCTTCATGGAACTCAA
ATAACCCTT---AACCCATCCCTTTTATGATCCTTAGGATTTGTTTTTTTATTTACTGTA
GGAGGACTTACTGGAGTAATCCTGGCTAATTCATCTATTGACATTGTTCTTCATGATACT
TATTACGTA
>C.brevitarsis Cbrev-AB1 [GenBank:AB360994]
GCTATTTTATTATTATTATCTTTACCAGTATTGGCTGGAGCAATTACTATATTATTGACT
GATCGAAATATTAATACTTCTTTTTTTGATCCTGCAGGAGGAGGGGATCCAATTTTATAT
CAACATTTGTTTTGATTTTTTGGGCATCCAGAAGTTTATATTTTAATTTTACCTGGGTTT
GGGATTGTTTCTCATATTATTTGTCAAGAAAGAGGTAAAAAAGAAGCTTTTGGTGTATTG
GGAATAATGTATGCTATTGCTGCAATTGGTTTATTAGGATTTATTGTATGAGCTCATCAT
ATATTTACTGTTGGACTAGACGTAGATACTCGAGCTTATTTTACTTCTGCTACTATAATT
ATTGCTGTACCAACAGGAATTAAAATTTTTAGTTGAATGGCTACTATATATGGAACTCAA
TTAAATTTA---ACTCCTGCTTTATTATGATCTTTAGGGTTTGTATTTTTATTTACTGTA
GGAGGATTAACGGGGGTTGTTTTAGCTAATTCTTCAATTGATATTATTTTACATGATACA
TATTATGTA
>C.brevitarsis Cbrev-AB2 [GenBank:AB360995]
GCTATTTTATTATTATTATCTTTACCAGTATTGGCTGGAGCAATTACTATATTATTGACT
GATCGAAATATTAATACTTCTTTTTTTGATCCTGCAGGAGGAGGGGATCCAATTTTATAT
CAACATTTGTTTTGATTTTTTGGGCATCCAGAAGTTTATATTTTAATTTTACCTGGGTTT
GGGATTGTTTCTCATATTATTTGTCAAGAAAGAGGTAAAAAAGAAGCTTTTGGTGTATTG
GGAATAATGTATGCTATTGCTGCAATTGGTTTATTAGGATTTATTGTATGAGCTCATCAT
ATATTTACTGTTGGACTAGACGTAGATACTCGAGCTTATTTTACTTCTGCTACTATAATT
ATTGCTGTACCAACAGGAATTAAAATTTTTAGTTGAATGGCTACTATATATGGAACTCAA
TTAAATTTA---ACTCCTGCTTTATTATGATCTTTAGGGTTTGTATTTTTATTTACTGTA
GGAGGATTAACGGGGGTTGTTTTAGCTAATTCTTCAATTGATATTATTTTACATGATACA
TATTATGTA
>C.brevipalpis JAP [GenBank:AB360998]
GCTATTTTACTTTTATTATCTTTACCAGTTTTAGCTGGAGCAATTACAATATTATTAACA
GATCGAAATCTTAATACTTCCTTTTTTGACCCGGCAGGAGGTGGTGATCCTATTCTTTAC
CAGCATTTATTTTGATTTTTTGGTCACCCAGAAGTTTATATTTTAATTTTACCAGGATTT
GGTATTGTTTCTCATGTTATTTATAATGAAAGAAGAAAAAAAGAAGCTTTTGGATCTTTA
GGAATAATATATGCTATATCTACTATTGGATTATTAGGATTTATTGTCTGAGCACATCAT
ATATTTACAGTAGGAATAGATATTGATACTCGAGCTTATTTTACTTCAGCAACAATAATT
ATTGCGGTACCTACAGGAATTAAGGTATTTAGTTGATTTGCTTCATTATCGGGATCAAAA
TTTAATCAT---ACACCTGCTTTATTATGAAGTATTGGATTTTTATTTTTATTTACTGTA
GGAGGTTTAACAGGAGTAATTTTAGCTAACTCTTCTATTGACTTAGTATTACATGACACA
TATTATGTT
>C.cylindratus JAP1 [GenBank:AB361006]
GCTATTTTACTACTTCTCTCCCTTCCAGTTTTAGCAGGAGCTATTACAATACTTTTAACA
GATCGAAACATCAATACTTCATTTTTTGACCCAGCAGGAGGAGGTGACCCTATTCTTTAT
CAACACTTATTTTGATTTTTCGGACACCCAGAAGTTTACATTTTAATTTTACCAGGATTT
GGAATTGTGTCCCATATTATTACTCAAGAAAGAGGAAAAAATGAAACTTTTGGCCCCCTA
GGAATAATTTATGCCATAACAGCTATTGGGCTCCTAGGTTTTATCGTCTGAGCTCATCAT
ATATTCACAGTAGGAATAGATGTAGATACCCGAGCTTATTTTACCTCAGCTACAATAGTT
ATTGCAATTCCAACCGGAATCAAAATTTTTAGCTGAATCGCAACATTACAAGGAACCCAA

ATAATTTTT---TCAACATCCCTTCTATGATCCCTCGGATTTATTTTCCTATTTACAGTA
GGAGGATTAACTGGAGTTGTTTTAGCTAACTCTTCAATTGATATTATCTTACATGATACC
TATTATGTA
>C.cylindratus JAP2 [GenBank:AB361007]
GCTATTTTACTACTTCTCTCCCTTCCAGTTTTAGCAGGAGCTATTACAATACTTTTAACA
GATCGAAACATCAATACTTCATTTTTTGACCCAGCAGGAGGAGGTGACCCTATTCTTTAT
CAACACTTATTTTGATTTTTCGGGCACCCAGAAGTTTACATTTTAATTTTACCAGGATTT
GGAATTGTGTCCCATATTATTACTCAAGAAAGAGGAAAAAATGAAACTTTTGGCCCCCTA
GGAATAATTTATGCCATAACAGCTATTGGGCTCCTAGGTTTTATCGTCTGAGCTCATCAT
ATATTCACAGTAGGAATAGATGTAGATACCCGAGCTTATTTTACCTCAGCTACAATAGTT
ATTGCAATTCCAACCGGAATCAAAATTTTTAGCTGAATCGCAACATTACAAGGAACCCAA
ATAATTTTT---TCAACATCCCTTCTATGATCCCTCGGATTTATTTTCCTATTTACAGTA
GGAGGATTAACTGGAGTTGTTTTAGCTAACTCTTCAATTGATATTATCTTACATGATACC
TATTATGTA
>C.dubius JAP [GenBank:AB361000]
GCTATTTTATTACTTTTATCATTACCTGTATTAGCCGGAGCTATTACAATATTATTAACA
GATCGAAATATTAATACTTCTTTTTTTGACCCTGCAGGAGGAGGAGACCCTATTTTATAT
CAACATTTATTTTGATTTTTTGGACACCCAGAAGTTTACATTTTAATTTTACCTGGATTT
GGTATAATTTCTCATATTATTAGTCAAGAAAGAGGAAAAAAGGAAACCTTTGGTGCTTTA
GGTATAATTTATGCCATATTAGCTATTGGTTTATTAGGATTTATTGTTTGAGCACATCAT
ATATTCACTGTAGGTATAGATGTTGATACACGAGCATATTTTACTTCAGCTACTATAATT
ATTGCTGTTCCTACAGGAATTAAAATTTTTAGTTGATTAGCAACTTTACATGGAACTCAA
ATAACCTTA---AACCCATCTCTTTTATGATCATTAGGGTTTGTATTCTTATTTACTGTA
GGAGGATTAACAGGAGTAATTTTAGCTAATTCATCTATTGATATTGTTCTTCATGATACC
TATTATGTT
>C.humeralis JAP1 [GenBank:AB360992]
GCTATCTTACTTCTTCTCTCCCTACCTGTCCTAGCCGGAGCAATTACTATATTACTAACC
GACCGAAACATTAATACATCATTCTTTGACCCAGCAGGAGGAGGGGACCCAATTCTATAC
CAACATTTATTTTGATTTTTTGGCCACCCAGAAGTTTATATCCTCATTCTTCCTGGCTTT
GGAATAATCTCCCATATTATTAGCCATGAATGCGGGAAAAAAGAAACATTTGGAGCATTA
GGTATAATTTATGCTATATTCTCCATTGGACTATTAGGATTTATCGTTTGAGCCCACCAT
ATATTTACCGTAGGAATAGACATCGATACGCGAGCTTACTTTACTGCCGCCACTATAATT
ATCGCAGTACCCACAGGAATTAAAATCTTTAGCTGACTAGCCACCCTTCACGGAGCTCAA
TTAAACTTT---TCCCCTTCAATCCTATGATCATTAGGATTTGTGTTTCTATTCACTGTA
GGAGGATTAACAGGAGTTATTTTAGCTAACTCCTCCATTGATATTATTCTTCATGACACT
TATTATGTT
>C.humeralis JAP2 [GenBank:AB360993]
GCTATCTTACTTCTTCTCTCCCTACCTGTCCTAGCCGGAGCAATTACTATATTACTAACC
GACCGAAACATTAATACATCATTCTTTGACCCAGCAGGAGGAGGGGACCCAATTCTATAC
CAACATTTATTTTGATTTTTTGGCCACCCAGAAGTTTATATCCTCATTCTTCCTGGCTTT
GGAATAATCTCCCATATTATTAGCCATGAATGCGGGAAAAAAGAAACATTTGGAGCATTA
GGTATAATTTATGCTATATTCTCCATTGGACTATTAGGATTTATCGTTTGAGCCCACCAT
ATATTTACCGTAGGAATAGACATCGATACGCGAGCTTACTTTACTGCCGCCACTATAATT
ATCGCAGTACCCACAGGAATTAAAATCTTTAGCTGACTAGCCACCCTTCACGGAGCTCAA
TTAAACTTT---TCCCCTTCAATCCTATGATCATTAGGATTTGTGTTTCTATTCACTGTA
GGAGGATTAACAGGAGTTATTTTAGCTAACTCCTCCATTGATATTATTCTTCATGACACT
TATTATGTT
>C.humeralis JAP3 [GenBank:AB364651]
GCTATCTTACTTCTTCTCTCCCTACCTGTCCTAGCCGGAGCAATTACTATATTACTAACC
GACCGAAACATTAATACATCATTCTTTGACCCAGCAGGAGGAGGGGACCCAATTCTATAC
CAACATTTATTTTGATTTTTTGGCCACCCAGAAGTTTATATCCTCATTCTTCCTGGCTTT
GGAATAATCTCCCATATTATTAGCCATGAATGCGGGAAAAAAGAAACATTTGGAGCATTA
GGTATAATTTATGCTATATTCTCCATTGGACTATTAGGATTTATCGTTTGAGCCCACCAT
ATATTTACCGTAGGAATAGACATCGATACGCGAGCTTACTTTACTGCCGCCACTATAATT
ATCGCAGTACCCACAGGAATTAAAATCTTTAGCTGACTAGCCACCCTTCACGGAGCTCAA
TTAAACTTT---TCCCCTTCAATCCTATGATCATTAGGATTTGTGTTTCTATTCACTGTA
GGAGGATTAACAGGAGTTATTTTAGCTAACTCCTCCATTGATATTATTCTTCATGACACT
TATTATGTT
>C.jacobsoni JAP2 [GenBank:AB360991]
GCTGTATTATTATTATTATCATTACCAGTATTAGCTGGAGCTATTACAATATTATTAATA
GACCGTAATATTAATACTTCATTTTTTGATCCAAGAGGAGGTGGAGATCCAATTTTATAT
CAACATCTTTTTTGATTTTTTGGTCATCCTGAAGTTTATATTTTAATTTTACCAGGATTT
GGAATTATTTCTCATATCGTATTACATGAAAGAGGAAAAAAAATTGCTTTTGGAGCATTA

GGAATAATTTATGCTATAATTACTATTGGAATTTTAGGTTTTATTGTATGAGCTCATCAT
ATATTTACAGTTGGAATAGATGTAGATACTCGAGCATATTTTACATCAGCTACTATAATT
ATTGCTATTCCTACAGGAATTAAAATTTTTAGATGAATTGCTACCTTTCAAGGTAGAAAT
TTAAATCTT---TCTACTTCTTTACTTTGAACTTTAGGATTTTTATTTTTATTTACAATA
GGTGGACTGACAGGAGTAATTTTAGCTAATTCTTCTCTTGATATTGTATTACATGATACT
TATTATGTA
>C.jacobsoni JAP1 [GenBank:AB360990]
GCTGTATTATTATTATTATCATTACCAGTATTAGCTGGAGCTATTACAATATTATTAATA
GACCGTAATATTAATACTTCATTTTTTGATCCAAGAGGAGGTGGAGATCCAATTTTATAT
CAACATCTTTTTTGATTTTTTGGTCATCCTGAAGTTTATATTTTAATTTTACCAGGATTT
GGAATTATTTCTCATATCGTATTACATGAAAGAGGAAAAAAAATTGCTTTTGGAGCATTA
GGAATAATTTATGCTATAATTACTATTGGAATTTTAGGTTTTATTGTATGAGCTCATCAT
ATATTTACAGTTGGAATAGATGTAGATACTCGAGCATATTTTACATCAGCTACTATAATT
ATTGCTATTCCTACAGGAATTAAAATTTTTAGATGAATTGCTACCTTTCAAGGTAGAAAT
TTAAATCTT---TCTACTTCTTTACTTTGAACTTTAGGATTTTTATTTTTATTTACAGTA
GGTGGACTGACAGGAGTAATTCTAGCTAATTCTTCTCTTGATATTGTATTACATGATACT
TATTATGTA
>C.japonicus JAP1 [GenBank:AB361001]
GCAATTTTGTTACTTTTATCTTTACCAGTTTTAGCAGGAGCCATTACTATACTTTTAACA
GATCGTAATATTAATACTTCTTTTTTTGACCCTGCAGGAGGGGGAGACCCTATTTTATAC
CAGCACTTATTTTGATTTTTTGGACACCCAGAAGTTTATATTTTAATTCTCCCTGGATTC
GGGATAATTTCTCATATTATTAGTCAAGAAAGAGGGAAAAAAGAAACATTTGGAGCGCTA
GGAATAATTTACGCTATACTAGCTATTGGACTTTTAGGATTTATTGTTTGAGCTCATCAT
ATATTCACAGTAGGAATAGACGTCGATACACGAGCTTATTTCACATCAGCTACTATAATT
ATTGCTGTTCCGACAGGAATTAAAATTTTTAGCTGATTAGCTACCCTTCACGGAACACAA
ATAACTTTA---AATCCTTCTTTACTTTGGTCATTAGGATTTGTATTTTTATTTACAGTA
GGAGGTCTAACAGGAGTAATTTTAGCTAACTCTTCCATTGATATTGTTCTTCATGATACT
TATTATGTA
>C.japonicus JAP2 [GenBank:AB361002]
GCAATTTTGTTACTTTTATCTTTACCAGTTTTAGCAGGAGCCATTACTATACTTTTAACA
GATCGTAATATTAATACTTCTTTTTTTGACCCTGCAGGAGGGGGAGACCCTATTTTATAC
CAGCACTTATTTTGATTTTTTGGACACCCAGAAGTTTATATTTTAATTCTCCCTGGATTC
GGGATAATTTCTCATATTATTAGTCAAGAAAGAGGGAAAAAAGAAACATTTGGAGCGCTA
GGAATAATTTACGCTATACTAGCTATTGGACTTTTAGGATTTATTGTTTGAGCTCATCAT
ATATTCACAGTAGGAATAGACGTCGATACACGAGCTTATTTCACATCAGCTACTATAATT
ATTGCTGTTCCGACAGGAATTAAAATTTTTAGCTGATTAGCTACCCTTCACGGAACACAA
ATAACTTTA---AATCCTTCTTTACTTTGGTCATTAGGATTTGTATTTTTATTTACAGTA
GGAGGTCTAACAGGAGTAATTTTAGCTAACTCTTCCATTGATATTGTTCTTCATGATACT
TATTATGTA
>C.kibunensis JAP1 [GenBank:AB646610]
GCTATTCTTCTTTTATTATCTTTGCCAGTATTAGCAGGGGCTATTACAATACTTTTAACT
GATCGAAATATTAACACTTCATTTTTTGACCCTGCAGGAGGAGGCGACCCTATTTTGTAC
CAACACCTATTTTGATTCTTTGGTCACCCAGAAGTTTATATTTTAATTTTACCAGGATTC
GGAATAATTTCTCACATTATTAGCCAAGAAAGCGGGAAAAAGGAAACCTTTGGGTCTTTA
GGAATGATTTATGCTATACTAGCTATTGGCTTATTAGGATTTATTGTATGAGCCCATCAC
ATATTCACTGTAGGGATAGACGTAGACACCCGAGCTTATTTTACATCGGCAACTATAATT
ATTGCCGTCCCAACTGGCATTAAAATTTTTAGTTGACTAGCAACTCTCCATGGAACCCAA
ATAACGCTT---AACCCTTCACTTTTATGATCCTTAGGATTTGTATTTTTATTTACTGTT
GGGGGATTAACAGGGGTAATCCTAGCTAATTCTTCTATTGATATTGTCCTTCATGACACT
TATTATGTA
>C.kibunensis JAP2 [GenBank:AB646611]
GCTATTCTTCTTTTATTATCTTTGCCAGTATTAGCAGGGGCTATTACAATACTTTTAACT
GATCGAAATATTAACACTTCATTTTTTGACCCTGCAGGAGGAGGCGACCCTATTTTGTAC
CAACACCTATTTTGATTCTTTGGTCACCCAGAAGTTTATATTTTAATTTTACCAGGATTC
GGAATAATTTCTCACATTATTAGCCAAGAAAGCGGGAAAAAGGAAACCTTTGGGTCTTTA
GGAATGATTTATGCTATACTAGCTATTGGCTTATTAGGATTTATTGTATGAGCCCATCAC
ATATTCACTGTTGGGATAGACGTAGACACCCGAGCTTATTTTACATCGGCAACTATAATT
ATTGCCGTCCCAACTGGCATTAAAATTTTTAGTTGACTAGCAACTCTCCATGGAACCCAA
ATAACGCTT---AACCCTTCACTTTTATGATCCTTAGGATTTGTATTTTTATTTACTGTT
GGGGGATTAACAGGAGTAATCCTAGCTAATTCTTCTATTGATATTGTCCTTCATGACACT
TATTATGTA
>C.maculatus JAP1 [GenBank:AB360986]
GCAATTTTATTACTTTTATCCCTGCCCGTTTTAGCAGGGGCTATCACAATACTTTTAACA

GATCGAAATATTAATACTTCTTTTTTTGACCCCGCGGGAGGAGGGGACCCTATTTTATAC
CAACATCTATTTTGATTCTTCGGACACCCTGAAGTTTATATTTTAATTTTACCTGCATTC
GGCATTATTTCACATATTATCGCCCATGAGAGAGGGAAAAAGGAAGCATTCGGAGCTTTA
GCTATAATATATGCAATTACAACAATTGGACTTTTAGGATTTATTGTATGAGCTCATCAT
ATATTTACAGTAGGGTTAGACGTAGACACTCGAGCTTACTTTTCCTCTGCGACTATAGTA
ATTGCCGTTCCAACCGGAATTAAAATTTTTAGTTGAATGGCCACTCTTTACGGAGCCCAT
TGAAAATAC---ACCCCTTCATTGCTATGAGCGTTAGGATTTATTTTTTTATTTACCTTA
GGAGGATTAACAGGAATTATTTTAGCTAATTCGTCAATTGATATTGTTCTCCATGACACA
TACTATGTA
>C.maculatus JAP2 [GenBank:AB360987]
GCAATTTTATTACTTTTATCCCTGCCCGTTTTAGCAGGGGCTATCACAATACTTTTAACA
GATCGAAATATTAATACTTCTTTTTTTGACCCCGCGGGAGGAGGGGACCCTATTTTATAC
CAACATCTATTTTGATTCTTCGGACACCCTGAAGTTTATATTTTAATTTTACCTGCATTC
GGCATTATTTCACATATTATCGCCCATGAGAGAGGGAAAAAGGAAGCATTCGGAGCTTTA
GCTATAATATATGCAATTACAACAATTGGACTTTTAGGATTTATTGTATGAGCTCATCAT
ATATTTACAGTAGGGTTAGACGTAGACACTCGAGCTTACTTTTCCTCTGCGACTATAGTA
ATTGCCGTTCCAACCGGGATTAAAATTTTTAGTTGAATGGCCACTCTTTACGGAGCCCAT
TGAAAATAC---ACCCCTTCATTATTATGAGCGTTAGGATTTATTTTTTTATTTACCTTA
GGAGGGTTAACAGGAATTATTTTAGCTAATTCATCAATTGATATTGTTCTTCATGACACA
TACTATGTA
>C.matsuzawai JAP1 [GenBank:AB364649]
GCTATTTTACTTTTATTATCACTTCCAGTGTTAGCCGGAGCTATTACTATACTGCTTACT
GATCGAAATATTAATACATCATTTTTTGACCCTGCAGGAGGAGGAGACCCTATTCTATAC
CAGCATTTATTTTGATTTTTTGGCCATCCAGAGGTATACATTTTAATTTTGCCAGGATTT
GGGATAATTTCCCATATTATCAGCCAAGAAAGAGGGAAAAAAGAAACTTTTGGGGCTTTA
GGAATAATTTATGCTATACTTGCAATTGGATTATTAGGTTTTATCGTATGAGCCCACCAT
ATATTTACTGTAGGCATAGATATTGACACTCGAGCTTACTTTACATCTGCTACTATAATT
ATTGCAGTTCCCACAGGAATTAAAATTTTTAGCTGACTAGCTACTCTTCATGGAACTCAA
ATAATTTTT---ACCCCTCAAATTTTATGATCCCTAGGCTTTGTATTTTTATTTACATTA
GGGGGATTAACAGGAGTAATTTTAGCAAATTCTTCTATTGATATTATTCTTCACGATACC
TACTATGTA
>C.matsuzawai JAP2 [GenBank:AB364650]
GCTATTTTACTTTTATTATCACTTCCAGTATTAGCAGGAGCTATTACTATACTTCTTACT
GACCGAAATATTAATACATCATTTTTTGACCCTGCAGGAGGGGGCGACCCTATTTTATAC
CAACATCTATTTTGATTCTTTGGCCATCCAGAAGTATATATTTTAATTTTACCAGGATTT
GGTATAATTTCCCATATTATTAGACAAGAAAGAGGGAAAAAAGAAACCTTTGGGGCCTTA
GGAATAATCTATGCTATACTTGCAATTGGATTACTAGGTTTTATCGTATGAGCTCACCAT
ATATTTACTGTAGGAATAGATATTGACACTCGAGCTTACTTTACATCTGCTACTATAATT
ATTGCAGTTCCTACAGGAATTAAAATTTTTAGCTGACTAGCTACTCTTCATGGAACTCAA
ATAATTTTT---ACCCCACAAATTTTATGATCCCTCGGTTTTGTATTTTTATTTACATTA
GGAGGATTGACAGGAGTAATTTTAGCAAATTCTTCTATTGATATCATTCTACACGATACC
TATTATGTA
>C.nipponensis JAP [GenBank:AB360999]
GCTATTTTATTACTTTTATCTTTACCTGTATTAGCAGGAGCTATTACTATACTTTTAACA
GATCGAAATTTTAACACTTCATTTTTTGACCCCGCAGGAGGAGGAGACCCAATTCTATAC
CAACATTTATTTTGATTCTTTGGTCACCCAGAAGTTTATATTTTAATTTTACCAGGATTT
GGTATTATTTCTCATATTATTTCTCATGAAAGAGGAAAAAAAGAAAGTTTTGGAAACTTA
GGAATAATTTATGCTATAACAACTATTGGATTATTAGGTTTCATCGTATGAGCACACCAC
ATATTTACAGTAGGAATAGACGTAGATACCCGAGCTTATTTTACTTCAGCAACAATAATT
ATTGCTGTGCCTACAGGTATTAAAATTTTTAGTTGATTAGCAACTATTTATGGCTCTCAA
ATTATATTTAAAAATCCAGCTATTTTATGAGCTATTGGATTCGTATTTTTATTTACAATA
GGGGGATTAACAGGAGTAATTTTAGCTAATTCTTCCATTGATGTTATTCTTCATGATACA
TATTATGTA
>C.ohmorii JAP1 [GenBank:AB360976]
GCAATCCTTCTACTATTATCCCTTCCAGTCTTAGCCGGAGCCATCACGATATTACTAACA
GATCGAAATATTAATACTTCATTTTTTGATCCTGCAGGAGGAGGAGACCCTATTCTTTAC
CAACATTTATTTTGATTTTTTGGGCACCCAGAAGTTTATATCTTAATTTTACCAGGATTT
GGGATTATTTCTCATATTATTAGAAGAGAAAGAGGTAAAAAAGAATCTTTTGGAAATCTA
GGAATAATTTATGCAATAACTACAATTGGATTATTGGGATTTATCGTTTGAGCTCACCAT
ATATTTACAGTAGGAATAGATGTAGACACACGAGCATATTTTACATCAGCTACAATAATT
ATTGCTGTCCCCACAGGAATTAAAATTTTTAGCTGACTAGCATCAATAATAGGTTCAACA
AATCGTATA---ACATCTAGAACTCTTTGAGCTCTAGGATTTATTTATTTATTTACTTTA
GGAGGAATAACAGGTATTATCCTATCTAATGCCATTATTGATGTTATACTTCATGATACA

TATTATGTA
>C.ohmorii JAP2 [GenBank:AB360977]
GCAATCCTTCTACTATTATCCCTTCCAGTCTTAGCCGGAGCCATCACGATATTACTAACA
GATCGAAATATTAATACTTCATTTTTTGATCCTGCAGGAGGAGGAGACCCTATTCTTTAC
CAACATTTATTTTGATTTTTTGGGCACCCAGAAGTTTATATCTTAATTTTACCAGGATTT
GGGATTATTTCTCATATTATTAGAAGAGAAAGAGGTAAAAAAGAATCTTTTGGAAATCTA
GGAATAATTTATGCAATAACTACAATTGGATTATTGGGATTTATCGTTTGAGCTCACCAT
ATATTTACAGTAGGAATAGATGTAGACACACGAGCATATTTTACATCAGCTACAATAATT
ATTGCTGTCCCCACAGGAATTAAAATTTTTAGCTGACTAGCATCAATAATAGGTTCAACA
AATCGTATA---ACATCTAGAACTCTTTGAGCTCTAGGATTTATTTATTTATTTACTTTA
GGAGGAATAACAGGTATTATCCTATCTAATGCCATTATTGATGTTATACTTCATGATACA
TATTATGTA
>C.oxystoma JAP1 [GenBank:AB360978]
GCTATTCTTTTACTACTTTCTTTACCTGTTCTAGCTGGAGCTATTACTATATTATTAACT
GACCGTAATATTAATACTTCATTCTTTGACCCTGCAGGAGGGGGAGACCCTATTCTTTAC
CAACATTTATTTTGATTTTTTGGACACCCTGAAGTTTATATTTTAATTTTACCAGGATTT
GGAATAATTTCTCATATTATTAGCCAAGAAAGAGGTAAAAAGGAAACATTTGGTTCTTTA
GGAATAATTTATGCTATACTAGCTATTGGGCTATTAGGATTTATTGTATGAGCTCATCAC
ATATTTACTGTTGGAATAGACGTAGATACACGAGCTTATTTTACTTCAGCTACAATAATT
ATTGCTGTACCAACCGGTATTAAAATTTTTAGTTGATTAGCTACTCTTCATGGTACTCAA
ATAACTTTA---ACTCCTTCTCTTTTATGAGCTTTAGGATTTGTATTCTTATTTACAGTA
GGAGGTTTAACAGGAGTAATTTTGGCTAATTCTTCTATTGATATTGTATTACATGATACA
TATTATGTT
>C.oxystoma JAP2 [GenBank:AB360979]
GCTATTCTTTTACTACTTTCTTTACCTGTTCTAGCTGGAGCTATTACTATATTATTAACT
GACCGTAATATTAATACTTCATTCTTTGACCCTGCAGGAGGGGGAGACCCTATTCTTTAC
CAACATTTATTTTGATTTTTTGGACACCCTGAAGTTTATATTTTAATTTTACCAGGATTT
GGAATAATTTCTCATATTATTAGCCAAGAAAGAGGTAAAAAGGAAACATTTGGTTCTTTA
GGAATAATTTATGCTATACTAGCTATTGGGCTATTAGGATTTATTGTATGAGCCCATCAC
ATATTTACTGTTGGGATAGACGTAGATACACGAGCTTATTTTACTTCAGCTACAATAATT
ATTGCTGTACCAACCGGTATTAAAATTTTTAGTTGATTAGCTACTCTTCATGGTACTCAA
ATAACTTTA---ACTCCTTCTCTTTTATGAGCTTTAGGATTTGTATTCTTATTTACAGTA
GGGGGTTTAACAGGAGTAATTTTGGCTAATTCTTCTATTGATATCGTATTACATGATACA
TATTATGTT
>C.oxystom JAP3 [GenBank:AB360980]
GCTATTCTTTTACTACTTTCTTTACCTGTTCTAGCTGGAGCTATTACTATATTATTAACT
GACCGTAATATTAATACTTCATTCTTTGACCCTGCAGGAGGGGGAGACCCTATTCTTTAC
CAACATTTATTTTGATTTTTTGGACACCCTGAAGTTTATATTTTAATTTTACCAGGATTT
GGGATAATTTCTCATATTATTAGCCAAGAAAGAGGTAAAAAGGAAACATTTGGTTCTTTA
GGAATAATTTATGCTATACTAGCTATTGGACTATTAGGATTTATTGTATGAGCTCATCAC
ATATTTACTGTTGGGATAGACGTAGATACACGAGCTTATTTTACTTCAGCTACAATAATT
ATTGCTGTACCAACCGGTATTAAAATTTTTAGTTGATTAGCTACTCTTCATGGTACTCAA
ATAACTTTA---ACTCCTTCTCTTTTATGAGCTTTAGGATTTGTATTCTTATTTACAGTA
GGGGGTTTAACAGGAGTAATTTTGGCTAATTCTTCTATTGATATCGTATTACATGATACA
TATTATGTT
>C.oxystoma JAP4 [GenBank:AB360981]
GCTATTCTTTTACTACTTTCTTTACCTGTTCTAGCTGGAGCTATTACTATATTATTAACT
GACCGTAATATTAATACTTCATTCTTTGACCCTGCAGGAGGGGGAGACCCTATTCTTTAC
CAACATTTATTTTGATTTTTTGGACACCCTGAAGTTTATATTTTAATTTTACCAGGATTT
GGGATAATTTCTCATATTATTAGCCAAGAAAGAGGTAAAAAGGAAACATTTGGTTCTTTA
GGAATAATTTATGCTATACTAGCTATTGGGTTATTAGGATTTATTGTATGAGCTCATCAC
ATATTTACTGTTGGAATAGACGTAGATACACGAGCTTATTTTACTTCAGCTACAATAATT
ATTGCTGTACCAACCGGTATTAAAATTTTTAGTTGATTAGCTACTCTTCATGGTACTCAA
ATAACTTTA---ACTCCTTCTCTTTTATGAGCTTTAGGATTTGTATTCTTATTTACAGTA
GGGGGTTTAACAGGAGTAATTTTGGCTAATTCTTCTATTGATATCGTATTACATGATACA
TATTATGTT
>C.oxystoma JAP5 [GenBank:AB360982]
GCTATTCTTTTACTACTTTCTTTACCTGTTCTAGCTGGAGCTATTACTATATTATTAACT
GACCGTAATATTAATACTTCATTTTTTGACCCTGCAGGAGGGGGAGACCCTATTCTTTAC
CAACATTTATTTTGATTTTTTGGACACCCTGAAGTTTATATTTTAATTTTACCAGGATTT
GGGATAATTTCTCATATTATTAGCCAAGAAAGAGGTAAAAAGGAAACATTTGGTTCTTTA
GGAATAATTTATGCTATACTAGCTATTGGGCTATTAGGATTTATTGTATGAGCTCATCAC
ATATTTACTGTCGGGATAGACGTAGATACACGAGCTTATTTTACTTCAGCTACAATAATT

ATTGCTGTACCAACCGGTATTAAAATTTTTAGTTGATTAGCTACTCTTCATGGTACTCAA
ATAACTTTA---ACTCCTTCTCTTTTATGAGCTTTAGGATTTGTATTCTTATTTACAGTA
GGAGGTTTAACAGGAGTAATTTTGGCTAATTCTTCTATTGATATCGTATTACATGATACA
TATTATGTT
>C.oxystoma JAP6 [GenBank:AB360983]
GCTATTCTTTTACTACTTTCTTTACCTGTTCTAGCTGGAGCTATTACTATATTATTAACT
GACCGTAATATTAATACTTCATTTTTTGACCCTGCAGGAGGGGGAGACCCTATTCTTTAC
CAACATTTATTTTGATTTTTTGGACACCCTGAAGTTTATATTTTAATTTTACCAGGATTT
GGGATAATTTCTCATATTATTAGCCAAGAAAGAGGTAAAAAGGAAACATTTGGTTCTTTA
GGAATAATTTATGCTATACTAGCTATTGGGCTATTAGGATTTATTGTATGAGCTCATCAC
ATATTTACTGTCGGGATAGACGTAGATACACGAGCTTATTTTACTTCAGCTACAATAATT
ATTGCTGTACCAACCGGTATTAAAATTTTTAGTTGATTAGCTACTCTTCATGGTACTCAA
ATAACTTTA---ACTCCTTCTCTTTTATGAGCTTTAGGATTTGTATTCTTATTTACAGTA
GGAGGTTTAACAGGAGTAATTTTGGCTAATTCTTCTATTGATATCGTATTACATGATACA
TATTATGTT
>C.oxystoma JAP7 [GenBank:AB360984]
GCTATTCTTTTACTACTTTCTTTACCTGTTCTAGCTGGAGCTATTACTATATTATTAACT
GACCGTAATATTAATACTTCATTCTTTGACCCTGCAGGAGGGGGAGACCCTATTCTTTAC
CAACATTTATTTTGATTTTTTGGACACCCTGAAGTTTATATTTTAATTTTACCAGGATTT
GGGATAATTTCTCATATTATTAGCCAAGAAAGAGGTAAAAAGGAAACATTTGGTTCTTTA
GGAATAATTTATGCTATACTAGCTATTGGGCTATTAGGATTTATTGTATGAGCTCATCAC
ATATTTACTGTTGGAATAGACGTAGATACACGAGCTTATTTTACTTCAGCTACAATAATT
ATTGCTGTACCAACCGGTATTAAAATTTTTAGTTGATTAGCTACTCTTCATGGTACTCAA
ATAACTTTA---ACTCCTTCTCTTTTATGAGCTTTAGGATTTGTATTCTTATTTACAGTA
GGGGGTTTAACAGGAGTAATTTTGGCTAATTCTTCTATTGATATCGTATTACATGATACA
TATTATGTT
>C.oxystoma JAP8 [GenBank:AB360985]
GCTATTCTTTTACTACTTTCTTTACCTGTTCTAGCTGGGGCTATTACTATATTATTAACT
GACCGTAATATTAATACTTCATTCTTTGACCCTGCAGGAGGGGGAGACCCTATTCTTTAC
CAACATTTATTTTGATTTTTTGGACACCCTGAAGTTTATATTTTAATTTTACCAGGATTC
GGGATAATTTCTCATATTATTAGCCAAGAAAGAGGTAAAAAGGAAACATTTGGTTCTTTA
GGAATAATTTATGCTATACTAGCTATTGGGCTATTAGGATTTATTGTATGAGCTCATCAC
ATATTTACTGTTGGGATAGACGTAGATACACGAGCTTATTTTACTTCAGCTACAATAATT
ATTGCTGTACCAACCGGTATTAAAATTTTTAGTTGATTAGCTACTCTTCATGGTACTCAA
ATAACTTTA---ACTCCTTCTCTTTTATGAGCTTTAGGATTTGTATTCTTATTTACAGTA
GGGGGTTTGACAGGAGTAATTTTGGCTAATTCTTCTATTGATATCGTATTACATGATACA
TATTATGTT
>C.peregrinus JAP [GenBank:AB361003]
GCTATTCTTTTACTTTTATCTCTTCCAGTTCTAGCCGGAGCTATTACTATACTTTTAACA
GATCGAAATATTAATACTACTTTCTTTGACCCAACAGGAGGAGGAGACCCAATTTTATAC
CAACATTTATTTTGATTTTTTGGACATCCAGAAGTCTATATTCTTATTTTACCTGGATTT
GGTATTGTTTCTCATATTATATCTCAAGAAAGAGGAAAAAAGGAAGCTTTTGGGGCATTA
GGAATAATTTATGCTATAACAACTATTGGACTTTTAGGATTTATTGTATGAGCACACCAC
ATATTTACAGTTGGAATAGATGTTGATACACGAGCATACTTCACTTCTGCTACAATAATT
ATTGCTATTCCCACAGGAATTAAAATTTTTAGATGATTAGCAACAATTTTAGGATCCCCT
CTTTCTTCAATTAATCCTCCTTTAATATGATCATTAGGATTTGTATTCTTATTCACTATT
GGGGGTTTAACTGGAATTATCCTAGCCAATTCATCAATTGATACTGTTTTACACGATACT
TATTATGTA
>C.pictimargo JAP1 [GenBank:AB646612]
GCCATCTTACTATTACTTTCATTACCTGTCTTAGCAGGAGCAATTACTATACTTTTGACC
GATCGAAATATTAATACTTCTTTTTTTGACCCAGCTGGGGGAGGAGACCCAATTCTTTAC
CAACACTTATTTTGATTCTTTGGACACCCAGAAGTATATATTTTAATTCTTCCAGGGTTT
GGTATAATTTCTCATATTATTAGTCAAGAAAGAGGTAAAAAAGAAACTTTTGGGGCTCTT
GGTATAATTTATGCAATATTAGCTATTGGACTATTAGGATTTATTGTATGGGCCCACCAC
ATATTTACAGTAGGAATAGATGTAGATACACGAGCATATTTCACTTCAGCTACTATAATT
ATTGCTGTACCTACTGGTATTAAAATTTTTAGTTGACTTGCAACTCTTCACGGAACACAA
ATAACCCTT---AATCCATCTTTACTATGATCCCTAGGATTTGTATTTTTATTTACTGTG
GGAGGATTAACTGGAGTAATTTTAGCTAATTCTTCTATTGACATTGTTCTTCATGATACT
TATTATGTA
>C.pictimargo JAP2 [GenBank:AB646613]
GCCATTTTACTATTACTTTCATTACCTGTCTTAGCAGGAGCAATTACTATACTTTTAACT
GATCGAAATATTAATACTTCTTTTTTTGACCCAGCTGGAGGAGGAGATCCAATTCTTTAC
CAACACTTATTTTGATTCTTTGGACACCCAGAAGTATATATTCTAATTCTTCCAGGATTT

GGTATAATTTCTCATATTATTAGTCAAGAAAGAGGTAAAAAAGAAACTTTTGGGGCTCTT
GGTATAATTTATGCAATATTAGCTATTGGGCTATTAGGATTTATTGTATGAGCCCACCAC
ATATTTACAGTAGGAATAGATGTAGACACACGAGCATATTTCACTTCAGCTACTATAATT
ATTGCTGTACCTACTGGTATTAAAATTTTTAGTTGACTTGCGACTCTCCACGGAACACAA
ATAACCCTT---AACCCATCTTTACTATGATCCCTAGGATTTGTATTTTTATTTACTGTG
GGAGGATTAACTGGAGTAATTTTGGCTAATTCTTCTATTGACATTGTTCTTCATGATACT
TATTATGTA
>C.punctatus JAP1 [GenBank:AB360988]
GCTATTTTACTTCTTTTATCTTTACCAGTTTTAGCCGGAGCTATTACAATACTATTAACA
GACCGAAATATTAATACTTCATTTTTTGACCCAGCAGGAGGTGGAGACCCTATTTTATAT
CAACATTTATTTTGATTTTTCGGGCACCCTGAGGTATACATTTTAATTTTACCCGGATTC
GGGATAATTTCTCACATTATTGCCCAAGAAAGAGGGAAAAAAGAAACTTTTGGGGCCTTA
GGTATAATTTATGCCATATTAGCCATTGGCCTATTAGGATTCATTGTTTGAGCTCATCAT
ATATTTACTGTAGGAATAGATGTAGACACCCGGGCTTATTTCACCTCAGCAACTATAATT
ATTGCTGTACCAACAGGAATTAAAATTTTTAGTTGACTTGCTACTCTCCATGGTACTCAA
ATAAATTTA---AATGCATCTCTTTTGTGATCATTAGGTTTTGTATTTTTATTTACAGTA
GGGGGACTTACCGGAGTAATTTTAGCTAATTCATCAATTGACATTGTTCTTCATGATACT
TATTATGTA
>C.punctatus JAP2 [GenBank:AB360989]
GCTATTTTACTTCTTTTATCTTTACCAGTTTTAGCCGGAGCTATTACAATACTATTAACA
GACCGAAACATTAATACTTCATTTTTTGACCCAGCAGGAGGTGGAGACCCTATTTTATAT
CAACATTTATTTTGATTTTTCGGGCACCCTGAGGTATACATTTTAATTTTACCCGGATTC
GGGATAATTTCTCACATTATTGCCCAAGAAAGAGGGAAAAAAGAAACTTTTGGGGCCTTA
GGTATAATTTATGCCATACTAGCCATTGGCCTATTAGGATTCATTGTTTGAGCTCATCAT
ATATTTACTGTAGGAATAGATGTAGACACCCGGGCTTATTTCACCTCAGCAACTATAATT
ATTGCTGTACCAACAGGAATTAAAATTTTTAGTTGACTTGCTACTCTCCATGGTACTCAA
ATAAATTTA---AATGCATCTCTTTTATGATCATTAGGTTTTGTATTTTTATTTACAGTA
GGGGGACTTACCGGAGTAATTTTAGCTAATTCATCAATTGACATTGTTCTTCATGATACT
TATTATGTA
>C.wadai JAP1 [GenBank:AB360996]
GCTATTTTACTATTATTATCTTTACCAGTTTTAGCCGGAGCTATCACAATACTTTTAACT
GATCGAAATATTAATACTTCCTTTTTCGATCCAGCAGGAGGAGGTGACCCCATTTTATAT
CAACATTTATTTTGATTCTTTGGTCATCCAGAAGTATATATTTTAATTTTACCTGGATTT
GGAATAATCTCCCATATTTTAAGTCAAGAAAGAGGGAAAAAGGAAACATTCGGAGCTTTA
GGGATAATTTATGCTATACTAGCAATTGGATTATTAGGTTTTATTGTTTGAGCTCATCAT
ATATTTACAGTTGGAATAGACGTAGACACCCGAGCTTATTTTACATCAGCAACTATAATT
ATTGCTGTACCTACTGGAATTAAAATTTTTAGTTGATTAGCAACAATCTATGGAACTCAA
TTAACTCTT---AATGCCTCATTACTTTGATCTCTAGGATTTGTATTTTTATTTACTATG
GGAGGATTAACAGGAGTAGTGCTAGCTAATTCTTCTATTGATGTAGTTCTTCATGATACT
TACTATGTA
>C.wadai JAP2 [GenBank:AB360997]
GCTATTTTACTATTATTATCTTTACCAGTTTTAGCCGGAGCTATCACAATACTTTTAACT
GATCGAAATATTAATACTTCCTTTTTCGATCCAGCAGGAGGAGGTGACCCCATTTTATAT
CAACATTTATTTTGATTCTTTGGTCATCCAGAAGTATATATTTTAATTTTACCTGGATTT
GGAATAATCTCCCATATTTTAAGTCAAGAAAGAGGGAAAAAGGAAACATTCGGAGCTTTA
GGGATAATTTATGCTATACTAGCAATTGGATTATTAGGTTTTATTGTTTGAGCTCATCAT
ATATTTACAGTTGGAATAGACGTAGACACCCGAGCTTATTTTACATCAGCAACTATAATT
ATTGCTGTACCTACTGGAATTAAAATTTTTAGTTGATTAGCAACAATCTATGGAACTCAA
TTAACTCTT---AATGCCTCATTACTTTGATCTCTAGGATTTGTATTTTTATTTACTATG
GGAGGATTAACAGGAGTAGTGCTAGCTAATTCTTCTATTGATGTAGTTCTTCATGATACT
TACTATGTA
>C.wadai JAP3 [GenBank:AB361005]
GCTATTTTACTATTATTATCTTTACCAGTTTTAGCCGGAGCTATCACAATACTTTTAACT
GATCGAAATATTAATACTTCCTTTTTCGATCCAGCAGGAGGAGGTGACCCCATTTTATAT
CAACATTTATTTTGATTCTTTGGTCATCCAGAAGTATATATTTTAATTTTACCTGGATTT
GGAATAATCTCCCATATTTTAAGTCAAGAAAGAGGGAAAAAGGAAACATTCGGAGCTTTA
GGGATAATTTATGCTATACTAGCAATTGGATTATTAGGTTTTATTGTTTGAGCTCATCAT
ATATTTACAGTTGGAATAGACGTAGACACCCGAGCTTATTTTACATCAGCAACTATAATT
ATTGCTGTACCTACTGGAATTAAAATTTTTAGTTGATTAGCAACAATCTATGGAACTCAA
TTAACTCTT---AATGCCTCATTACTTTGATCTCTAGGATTTGTATTTTTATTTACTATG
GGAGGATTAACAGGAGTAGTGCTAGCTAATTCTTCTATTGATGTAGTTCTTCATGATACT
TACTATGTA
>Anopheles gambiae [GenBank:Nc_002084][1991..2537]

GCAGTATTATTATTATTATCATTACCAGTATTAGCAGGAGCTATTACTATATTATTAACT
GATCGAAATTTAAATACATCTTTCTTTGATCCAGCAGGAGGAGGTGATCCAATTTTATAT
CAACACTTATTCTGATTTTTTGGTCATCCAGAAGTATATATTTTAATTTTACCTGGATTC
GGAATAATTTCGCATATTATTACTCAAGAAAGTGGAAAGAAGGAAACATTTGGAAACTTA
GGAATAATCTATGCTATACTAGCAATTGGCTTACTTGGATTTATTGTTTGAGCTCATCAT
ATATTTACAGTTGGAATAGACGTTGATACTCGAGCTTACTTTACATCAGCAACTATAATT
ATTGCTGTGCCAACTGGAATTAAGATTTTTAGTTGATTAGCTACATTACACGGAACACAA
TTGACTTAT---AGCCCAGCTATATTGTGAGCATTTGGATTCGTTTTCTTATTTACAGTT
GGTGGTCTAACAGGAGTTGTACTAGCTAATTCATCTATTGATATTGTTCTTCACGATACT
TATTATGTT
>Anopheles quadriannulatus [GenBank:DQ792581]
GCAGTATTATTATTATTATCATTACCAGTATTAGCAGGAGCTATTACTATATTATTAACT
GATCGAAATTTAAATACATCTTTCTTTGATCCAGCAGGAGGAGGTGACCCAATTTTATAC
CAACATTTATTCTGATTTTTTGGTCATCCAGAAGTATATATTTTAATTTTACCTGGATTC
GGAATAATTTCACATATTATTACTCAAGAAAGTGGAAAGAAGGAAACATTTGGAAACTTA
GGGATAATCTATGCTATACTAGCAATTGGTTTACTTGGATTTATTGTTTGAGCCCATCAT
ATATTTACAGTTGGAATAGACGTTGATACTCGAGCTTACTTTACATCAGCAACTATAATT
ATTGCTGTACCAACTGGAATTAAGATTTTTAGTTGATTAGCTACATTACACGGAACACAA
TTAACTTAT---AGCCCAGCTATATTATGAGCATTTGGATTCGTTTTCTTATTTACAGTT
GGTGGTCTAACAGGAGTTGTACTAGCTAATTCATCTATTGATATTGTTCTTCACGATACT
TATTATGTT
>Anopheles dirus_D [GenBank:AJ877572]
GCAATTTTATTACTTTTATCTTTACCCGTATTAGCAGGAGCAATTACTATATTATTAACA
GACCGAAATTTAAATACTTCATTTTTTGATCCAGCTGGTGGAGGAGATCCTATTTTATAT
CAACACTTATTCTGATTTTTCGGACATCCAGAAGTTTACATTTTAATTTTACCTGGATTT
GGAATAATTTCTCATATTATTACTCAAGAAAGAGGAAAGAAGGAAACTTTTGGAAATTTA
GGAATAATTTATGCTATATTAGCAATTGGATTATTAGGATTTATTGTTTGAGCTCATCAT
ATATTTACTGTTGGAATAGACGTAGATACTCGAGCTTATTTTACTTCTGCAACTATAATT
ATTGCTGTACCAACTGGAATTAAAATTTTTAGTTGATTAGCAACATTACACGGAACACAA
TTAACTTAT---AGACCAGCTATACTTTGAGCATTTGGATTTGTTTTTTTATTTACAGTA
GGGGGTTTAACTGGAGTTGTACTGGCTAATTCATCAATTGATATTGTATTACATGATACT
TATTATGTA
>Anopheles janconnae [GenBank:HQ335348]
GCTGTATTATTATTACTATCATTACCTGTATTAGCTGGAGCTATTACTATATTATTAACT
GATCGAAATTTAAACACATCATTTTTTGATCCAGCCGGAGGAGGTGATCCTATTTTATAC
CAACATTTATTCTGATTTTTTGGACATCCAGAAGTTTACATTTTAATTCTACCAGGATTT
GGAATAATTTCTCACATTATTACTCAAGAAAGAGGAAAGAAGGAAACATTTGGAAATTTA
GGAATAATTTATGCTATATTAGCTATTGGATTATTAGGATTTATTGTTTGAGCTCATCAT
ATATTTACTGTTGGGATAGATGTAGATACACGAGCTTACTTCACATCAGCTACTATAATT
ATTGCTGTACCTACAGGAATTAAAATTTTTAGTTGATTAGCTACATTACATGGAACACAG
CTAACGTAT---AGCCCAGCTATACTTTGAGCATTTGGATTCGTATTTTTATTTACTGTA
GGAGGACTAACTGGAGTAGTTTTAGCTAATTCTTCTATTGATATTGTATTACATGATACT
TATTATGTA
