# Supplementary material for: Population Genetic Structure and Potential Incursion Pathways of the Bluetongue Virus Vector Culicoides brevitarsis (Diptera: Ceratopogonidae) in Australia
Source: PLoS One. 2016 Jan 15;11(1):e0146699. doi: 10.1371/journal.pone.0146699 (PMC4714883; doi:10.1371/journal.pone.0146699)
Supplement: S2 File — (PDF) [file pone.0146699.s002.pdf]

S2 File: Alignment of 184 amino acids of partial *Culicoides* mtDNA COI gene in fasta+gap format for used in phylogenetic analysis.

>Cbrev-01 [GenBank:KP201844]
AILLLLSLPVLAGAITMLLTDRNINTSFFDPAGGGDPILYQHLFWFFGHPEVYILILPGF
GIVSHIICQESGKKEAFGVLGMMYAIAAIGLLGFIVWAHHMFTVGLDVDTRAYFTSATMI
IAVPTGIKIFSWMATMYGTQLNL-TPALLWSLGFVFLFTVGGLTGIVLANSSLDIVLHDT
YYV
>Cbrev-02 [GenBank:KP201845]
AILLLLSLPVLAGAITMLLTDRNINTSFFDPAGGGDPILYQHLFWFFGHPEVYILILPGF
GIVSHIICQESGKKEAFGVLGMMYAIAAIGLLGFIVWAHHMFTVGLDVDTRAYFTSATMI
IAVPTGIKIFSWMATMYGTQLNL-TPALLWSLGFVFLFTVGGLTGIVLANSSLDIVLHDT
YYV
>Cbrev-03 [GenBank:KP201846]
AILLLLSLPVLAGAITMLLTDRNINTSFFDPAGGGDPILYQHLFWFFGHPEVYILILPGF
GIVSHIICQESGKKEAFGVLGMMYAIAAIGLLGFIVWAHHMFTVGLDVDTRAYFTSATMI
IAVPTGIKIFSWMATMYGTQLNL-TPALLWSLGFVFLFTVGGLTGIVLANSSLDIVLHDT
YYV
>Cbrev-04 [GenBank:KP201847]
AILLLLSLPVLAGAITMLLTDRNINTSFFDPAGGGDPILYQHLFWFFGHPEVYILILPGF
GIVSHIICQESGKKEAFGVLGMMYAIAAIGLLGFIVWAHHMFTVGLDVDTRAYFTSATMI
IAVPTGIKIFSWMATMYGTQLNL-TPALLWSLGFVFLFTVGGLTGIVLANSSLDIVLHDT
YYV
>Cbrev-05 [GenBank:KP201848]
AILLLLSLPVLAGAITMLLTDRNINTSFFDPAGGGDPILYQHLFWFFGHPEVYILILPGF
GIVSHIICQESGKKEAFGVLGMMYAIAAIGLLGFIVWAHHMFTVGLDVDTRAYFTSATMI
IAVPTGIKIFSWMATMYGTQLNL-TPALLWSLGFVFLFTVGGLTGIVLANSSLDIVLHDT
YYV
>Cbrev-06 [GenBank:KP201849]
AILLLLSLPVLAGAITMLLTDRNINTSFFDPAGGGDPILYQHLFXFFGHPEVYILILPGF
GIVSHIICQESGKKEAFGVLGMMYAIAAIGLLGFIVWAHHMFTVGLDVDTRAYFTSATMI
IAVPTGIKIFSWMATMYGTQLNL-TPALLWSLGFVFLFTVGGLTGIVLANSSLDIVLHDT
YYV
>Cbrev-07 [GenBank:KP2018450]
AILLLLSLPVLAGAITMLLTDRNINTSFFDPAGGGDPILYQHLFWFFGHPEVYILILPGF
GIVSHIICQESGKKEAFGVLGMMYAIAAIGLLGFIVWAHHMFTVGLDVDTRAYFTSATMI
IAVPTGIKIFSWMATMYGTQLNL-TPALLWSLGFVFLFTVGGLTGIVLANSSLDIVLHDT
YYV
>Cbrev-08 [GenBank:KP2018451]
AILLLLSLPVLAGAITMLLTDRNINTSFFDPAGGGDPILYQHLFWFFGHPEVYILILPGF
GIVSHIICQESGKKEAFGVLGMMYAIAAIGLLGFIVWAHHMFTVGLDVDTRAYFTSATMI
IAVPTGIKIFSWMATMYGTQLNL-TPALLWSLGFVFLFTVGGLTGIVLANSSLDIVLHDT
YYG
>Cbrev-09 [GenBank:KP2018452]
AILLLLSLPVLAGAITMLLTDRNINTSFFDPAGGGDPILYQHLFWFFGHPEVYILILPGF
GIVSHIICQESGKKEAFGVLGMMYAIAAIGLLGFIVWAHHMFTVGLDVDTRAYFTSATMI
IAVPTGIKIFSWMATMYGTQLNL-TPALLWSLGFVFLFTVGGLTGIVLANSSLDIVLHDT
YYV
>Cmarksi-01 [GenBank:KP2018453]
AILLFLSLPVLAGAITMLLTDRNINTSFFDPAGGGDPILYQHLFWFFGHPEVYILILPGF
GMISHIISQESGKKETFGSLGMIYAMLAIGLLGFIVWAHHMFTVGMDVDTRAYFTSATMI
IAVPTGIKIFSWLATLHGTQMTL-NPSLLWALGFVFLFTVGGLTGVILANSSIDIVLHDT
YYV
>Cmarksi-03 [GenBank:KP2018454]
AILLFLSLPVLAGAITMLLTDRNINTSFFDPAGGGDPILYQHLFWFFGHPEVYILILPGF
GMISHIISQESGKKETFGSLGMIYAMLAIGLLGFIVWAHHMFTVGMDVDTRAYFTSATMI
IAVPTGIKIFSWLATLHGTQMTL-NPSLLWALGFVFLFTVGGLTGVILANSSIDIVLHDT
YYV
>Cmarksi-4 [GenBank:KP2018455]
AILLFLSLPVLAGAITMLLTDRNINTSFFDPAGGGDPILYQHLFWFFGHPEVYILILPGF
GMISHIISQESGKKETFGSLGMIYAMLAIGLLGFIVWAXHMFTVGMDVDTRAYFTSATMI
IAVPTGIKIFSWLATLHGTQMTL-NPSLLWALGFVFLFTVGGLTGVILANSSIDIVLHDT

YYV
>Cmarksi-05 [GenBank:KP2018456]
AILLFLSLPVLAGAITMLLTDRNINTSFFDPAGGGDPILYQHLFWFFGHPEVYILILPGF
GMISHIISQESGKKETFGSLGMIYAMLAIGLLGFIVWAXHMFTVGMDVDTRAYFTSATMI
IAVPTGIKIFSWLATLHGTQMTL-NPSLLWALGFVFLFTVGGLTGVILANSSIDIVLHDT
YYV
>Cmarksi-06 [GenBank:KP2018457]
AILLFLSLPVLAGAITMLLTDRNINTSFFDPAGGGDPILYQHLFWFFGHPEVYILILPGF
GMISHIISQESGKKETFGSLGMIYAMLAIGLLGFIVWAHHMFTVGMDVDTRAYFTSATMI
IAVPTGIKIFSWLATLHGTQMTL-NPSLLWALGFVFLFTVGGLTGVILANSSIDIVLHDT
YYV
>Cmarksi-07 [GenBank:KP2018458]
AILLFLSLPVLAGAITMLLTDRNINTSFFDPAGGGDPILYQHLFWFFGHPEVYILILPGF
GMISHIISQESGKKETFGSLGMIYAMLAIGLLGFIVWAXHMFTVGMDVDTRAYFTSATMI
IAVPTGIKIFSWLATLHGTQMTL-NPSLLWALGFVFLFTVGGLTGVILANSSIDIVLHDT
YYV
>Cmarksi-08 [GenBank:KP2018459]
AILLFLSLPVLAGAITMLLTDRNINTSFFDPAGGGDPILYQHLFWFFGHPEVYILILPGF
GMISHIISQESGKKETFGSLGMIYAMLAIGLLGFIVWAXHMFTVGMDVDTRAYFTSATMI
IAVPTGIKIFSWLATLHGTQMTL-NPSLLWALGFVFLFTVGGLTGVILANSSIDIVLHDT
YYV
>Cmarksi-09 [GenBank:KP2018460]
AILLFLSLPVLAGAITMLLTDRNINTSFFDPAGGGDPILYQHLFWFFGHPEVYILILPGF
GMISHIISQESGKKETFGSLGMIYAMLAIGLLGFIVWAXHMFTVGMDVDTRAYFTSATMI
IAVPTGIKIFSWLATLHGTQMTL-NPSLLWALGFVFLFTVGGLTGVILANSSIDIVLHDT
YYV
>Cmarksi-10 [GenBank:KP2018461]
AILLFLSLPVLAGAITMLLTDRNINTSFFDPAGGGDPILYQHLFWFFGHPEVYILILPGF
GMISHIISQESGKKETFGSLGMIYAMLAIGLLGFIVWAHHMFTVGMDVDTRAYFTSATMI
IAVPTGIKIFSWLATLHGTQMTL-NPSLLWALGFVFLFTVGGLTGVILANSSIDIVLHDT
YYV
>Cmarksi-11 [GenBank:KP2018462]
AILLFLSLPVLAGAITMLLTDRNINTSFFDPAGGGDPILYQHLFWFFGHPEVYILILPGF
GMISHIISQESGKKETFGSLGMIYAMLAIGLLGFIVWAHHMFTVGMDVDTRAYFTSATMI
IAVPTGIKIFSWLATLHGTQMTL-NPSLLWALGFVFLFTVGGLTGVILANSSIDIVLHDT
YYV
>Cmarksi-12 [GenBank:KP2018463]
AILLFLSLPVLAGAITMLLTDRNINTSFFDPAGGGDPILYQHLFWFFGHPEVYILILPGF
GMISHIISQESGKKETFGSLGMIYAMLAIGLLGFIVWAHHMFTVGMDVDTRAYFTSATMI
IAVPTGIKIFSWLATLHGTQMTL-NPSLLWALGFVFLFTVGGLTGVILANSSIDIVLHDT
YYV
>Cmarksi-13 [GenBank:KP2018464]
AILLFLSLPVLAGAITMLLTDRNINTSFFDPAGGGDPILYQHLFWFFGHPEVYILILPGF
GMISHIISQESGKKETFGSLGMIYAMLAIGLLGFIVWAXHMFTVGMDVDTRAYFTSATMI
IAVPTGIKIFSWLATLHGTQMTL-NPSLLWALGFVFLFTVGGLTGVILANSSIDIVLHDT
YYV
>Cmarksi-14 [GenBank:KP2018465]
AILLFLSLPVLAGAITMLLTDRNINTSFFDPAGGGDPILYQHLFWFFGHPEVYILILPGF
GMISHIISQESGKKETFGSLGMIYAMLAIGLLGFIVWAHHMFTVGMDVDTRAYFTSATMI
IAVPTGIKIFSWLATLHGTQMTL-NPSLLWALGFVFLFTVGGLTGVILANSSIDIVLHDT
YYV
>Cmarksi-15 [GenBank:KP2018466]
AILLFLSLPVLAGAITMLLTDRNINTSFFDPAGGGDPILYQHLFWFFGHPEVYILILPGF
GMISHIISQESGKKETFGSLGMIYAMLAIGLLGFIVWAHHMFTVGMDVDTRAYFTSATMI
IAVPTGIKIFSWLATLHGTQMTL-NPSLLWALGFVFLFTVGGLTGVILANSSIDIVLHDT
YYV
>Cmarksi-16 [GenBank:KP2018467]
AILLFLSLPVLAGAITMLLTDRNINTSFFDPAGGGDPILYQHLFWFFGHPEVYILILPGF
GMISHIISQESGKKETFGSLGMIYAMLAIGLLGFIVWAHHMFTVGMDVDTRAYFTSATMI
IAVPTGIKIFSWLATLHGTQMTL-NPSLLWALGFVFXFTVGGLTGVILANSSIDIVLHDT
YYV
>Cpallid-221 [GenBank:KP2018468]
AILLLLSLPVLAGAITMLLTDRNINTSFFDPAGGGDPILYQHLFWFFGHPEVYILILPGF

GMISHIISQESGKKETFGSLGMIYAMLAIGLLGFIVWAHHMFTVGMDVDTRAYFTSATMI
IAVPTGIKIFSWLATLHGTQMTL-NPSLLWALGFVFLFTVGGLTGVILANSSIDIVLHDT
YYV
>Chenryi-02 [GenBank:KP2018469]
AILLLLSLPVLAGAITMLLTDRNINTSFFDPAGGGDPILYQHLFWFFGHPEVYILILPGF
GMISHIISQESGKKETFGSLEMIYAMLAIGLLGFIVWAHHMFTVGMDVDTRAYFTSATMI
IAVPTGIKIFSWLATLHGTQMTL-NPSLLWALEWVFLFTVGGLTGVILANSSXXXXXXXX
XXX
>Chenryi-01 [GenBank:KP2018470]
AILLLLSLPVLAGAITMLLTDRNINTSFFDPAGGGDPILYQHLFWFFGHPEVYILILPGF
GMISHIISQESGKKETFGSLGMIYAMLAIGLLGFIVWAHHMFTVGMDVDTRAYFTSATMI
IAVPTGIKIFSWLATLHGTQMTL-NPSLLWALGFVFLFTVGGLTGVILANSSIDIVLHDT
YYV
>Cbundye-01 [GenBank:KP2018471]
XXXXXXXXXXXXXXXXXLTDRNINTSFFDPAGGGDSILYQHLFWFFGHPEVYILILPGF
GMVSHIISQESGKKETFGSLGMIYAMLAIGLLGFIVWAHHMFTVGMDVDTRAYFTSATMI
IAVPTGIKIFSWLATLHGTQMTP-NASLLWALGFVFLFTVGSLTGVILANSSIXIGLHDT
YYV
>Cbunroo-01 [GenBank:KP2018472]
AILLXLSLPVLAGAITMLLTDRNINTSFFDPAGGGDPILYQHLFWFFGHPEVYILILPGF
GMISHIISQESGKKETFGSLGMIYAMLAIGLLGFIVWAHHMFTVGMDVDTRAYFTSATMI
IAVPTGIKIFSWLATLHGTQMTL-NPSLLWSLGFVFLFTVGGLTGVILANSSIDIVLHDT
YYV
>C.actoni JAP [GenBank:AB360971]
ALLLLLSLPVLAGAITMLLTDRNFNTSFFDPAGGGDPVLYQHLFWFFGHPEVYILILPAF
GIISHIMAGESGKKEPFGVLAMQYAISAIGLLGFFVWAHHMFTVGLDVDTRAYFSAATMV
IGIPTGIKIFSWLATIYGSPWQF-TPAMLWALGFIFLFTLGGLTGIVLSNTAIDIVLHDT
YYV
>C.arakawae JAP 1 [GenBank:AB360972]
AILLLLSLPVLAGAITMLLTDRNINTSFFDPAGGGDPILYQHLFWFFGHPEVYILILPGF
GMISHIISQESGKKETFGALGMIYAMLAIGLLGFIVWAHHMFTVGMDVDTRAYFTSATMI
IAVPTGIKIFSWLATLHGTQMTL-TPSLLWSLGFVFLFTVGGLTGVILANSSIDIILHDT
YYV
>C.arakawae JAP 2 [GenBank:AB360973]
AILLLLSLPVLAGAITMLLTDRNINTSFFDPAGGGDPILYQHLFWFFGHPEVYILILPGF
GMISHIISQESGKKETFGALGMIYAMLAIGLLGFIVWAHHMFTVGMDVDTRAYFTSATMI
IAVPTGIKIFSWLATLHGTQMTL-TPSLLWSLGFVFLFTVGGLTGVILANSSIDIILHDT
YYV
>C.arakawae JAP3 [GenBank:AB360974]
AILLLLSLPVLAGAITMLLTDRNINTSFFDPAGGGDPILYQHLFWFFGHPEVYILILPGF
GMISHIISQESGKKETFGALGMIYAMLAIGLLGFIVWAHHMFTVGMDVDTRAYFTSATMI
IAVPTGIKIFSWLATLHGTQMTL-TPSLLWSLGFVFLFTVGGLTGVILANSSIDIILHDT
YYV
>C.arakawae JAP4 [GenBank:AB360975]
AILLLLSLPVLAGAITMLLTDRNINTSFFDPAGGGDPILYQHLFWFFGHPEVYILILPGF
GMISHIISQESGKKETFGALGMIYAMLAIGLLGFIVWAHHMFTVGMDVDTRAYFTSATMI
IAVPTGIKIFSWLATLHGTQMTL-TPSLLWSLGFVFLFTVGGLTGVILANSSIDIILHDT
YYV
>C.arakawae JAP5 [GenBank:AB361004-Yng05_2
AILLLLSLPVLAGAITMLLTDRNINTSFFDPAGGGDPILYQHLFWFFGHPEVYILILPGF
GMISHIISQESGKKETFGALGMIYAMLAIGLLGFIVWAHHMFTVGMDVDTRAYFTSATMI
IAVPTGIKIFSWLATLHGTQMTL-TPSLLWSLGFVFLFTVGGLTGVILANSSIDIILHDT
YYV
>C.verbosus [GenBank:AB646615]
AILLLLSLPVLAGAITMLLTDRNINTSFFDPAGGGDPILYQHLFWFFGHPEVYILILPGF
GMISHIISQESGKKETFGSLGMIYAMLAIGLLGFIVWAHHMFTVGMDVDTRAYFTSATMI
IAVPTGIKIFSWLATLHGTQMTL-NPSLLWSLGFVFLFTVGGLTGVILANSSIDIVLHDT
YYV
>C.brevitarsis Cbrev-AB1 [GenBank:AB360994]
AILLLLSLPVLAGAITMLLTDRNINTSFFDPAGGGDPILYQHLFWFFGHPEVYILILPGF
GIVSHIICQESGKKEAFGVLGMMYAIAAIGLLGFIVWAHHMFTVGLDVDTRAYFTSATMI
IAVPTGIKIFSWMATMYGTQLNL-TPALLWSLGFVFLFTVGGLTGVVLANSSIDIILHDT
YYV

>C.brevitarsis Cbrev-AB2 [GenBank:AB360995]
AILLLLSLPVLAGAITMLLTDRNINTSFFDPAGGGDPILYQHLFWFFGHPEVYILILPGF
GIVSHIICQESGKKEAFGVLGMMYAIAAIGLLGFIVWAHHMFTVGLDVDTRAYFTSATMI
IAVPTGIKIFSWMATMYGTQLNL-TPALLWSLGFVFLFTVGGLTGVVLANSSIDIILHDT
YYV
>C.brevipalpis JAP [GenBank:AB360998]
AILLLLSLPVLAGAITMLLTDRNLNTSFFDPAGGGDPILYQHLFWFFGHPEVYILILPGF
GIVSHVIYNESSKKEAFGSLGMMYAMSTIGLLGFIVWAHHMFTVGMDIDTRAYFTSATMI
IAVPTGIKVFSWFASLSGSKFNH-TPALLWSIGFLFLFTVGGLTGVILANSSIDLVLHDT
YYV
>C.cylindratus JAP1 [GenBank:AB361006]
AILLLLSLPVLAGAITMLLTDRNINTSFFDPAGGGDPILYQHLFWFFGHPEVYILILPGF
GIVSHIITQESGKNETFGPLGMIYAMTAIGLLGFIVWAHHMFTVGMDVDTRAYFTSATMV
IAIPTGIKIFSWIATLQGTQMIF-STSLLWSLGFIFLFTVGGLTGVVLANSSIDIILHDT
YYV
>C.cylindratus JAP2 [GenBank:AB361007]
AILLLLSLPVLAGAITMLLTDRNINTSFFDPAGGGDPILYQHLFWFFGHPEVYILILPGF
GIVSHIITQESGKNETFGPLGMIYAMTAIGLLGFIVWAHHMFTVGMDVDTRAYFTSATMV
IAIPTGIKIFSWIATLQGTQMIF-STSLLWSLGFIFLFTVGGLTGVVLANSSIDIILHDT
YYV
>C.dubius JAP [GenBank:AB361000]
AILLLLSLPVLAGAITMLLTDRNINTSFFDPAGGGDPILYQHLFWFFGHPEVYILILPGF
GMISHIISQESGKKETFGALGMIYAMLAIGLLGFIVWAHHMFTVGMDVDTRAYFTSATMI
IAVPTGIKIFSWLATLHGTQMTL-NPSLLWSLGFVFLFTVGGLTGVILANSSIDIVLHDT
YYV
>C.humeralis JAP1 [GenBank:AB360992]
AILLLLSLPVLAGAITMLLTDRNINTSFFDPAGGGDPILYQHLFWFFGHPEVYILILPGF
GMISHIISHECGKKETFGALGMIYAMFSIGLLGFIVWAHHMFTVGMDIDTRAYFTAATMI
IAVPTGIKIFSWLATLHGAQLNF-SPSILWSLGFVFLFTVGGLTGVILANSSIDIILHDT
YYV
>C.humeralis JAP2 [GenBank:AB360993]
AILLLLSLPVLAGAITMLLTDRNINTSFFDPAGGGDPILYQHLFWFFGHPEVYILILPGF
GMISHIISHECGKKETFGALGMIYAMFSIGLLGFIVWAHHMFTVGMDIDTRAYFTAATMI
IAVPTGIKIFSWLATLHGAQLNF-SPSILWSLGFVFLFTVGGLTGVILANSSIDIILHDT
YYV
>C.humeralis JAP3 [GenBank:AB364651]
AILLLLSLPVLAGAITMLLTDRNINTSFFDPAGGGDPILYQHLFWFFGHPEVYILILPGF
GMISHIISHECGKKETFGALGMIYAMFSIGLLGFIVWAHHMFTVGMDIDTRAYFTAATMI
IAVPTGIKIFSWLATLHGAQLNF-SPSILWSLGFVFLFTVGGLTGVILANSSIDIILHDT
YYV
>C.jacobsoni JAP2 [GenBank:AB360991]
AVLLLLSLPVLAGAITMLLMDRNINTSFFDPSGGGDPILYQHLFWFFGHPEVYILILPGF
GIISHIVLHESGKKIAFGALGMIYAMITIGILGFIVWAHHMFTVGMDVDTRAYFTSATMI
IAIPTGIKIFSWIATFQGSNLNL-STSLLWTLGFLFLFTMGGLTGVILANSSLDIVLHDT
YYV
>C.jacobsoni JAP1 [GenBank:AB360990]
AVLLLLSLPVLAGAITMLLMDRNINTSFFDPSGGGDPILYQHLFWFFGHPEVYILILPGF
GIISHIVLHESGKKIAFGALGMIYAMITIGILGFIVWAHHMFTVGMDVDTRAYFTSATMI
IAIPTGIKIFSWIATFQGSNLNL-STSLLWTLGFLFLFTVGGLTGVILANSSLDIVLHDT
YYV
>C.japonicus JAP1 [GenBank:AB361001]
AILLLLSLPVLAGAITMLLTDRNINTSFFDPAGGGDPILYQHLFWFFGHPEVYILILPGF
GMISHIISQESGKKETFGALGMIYAMLAIGLLGFIVWAHHMFTVGMDVDTRAYFTSATMI
IAVPTGIKIFSWLATLHGTQMTL-NPSLLWSLGFVFLFTVGGLTGVILANSSIDIVLHDT
YYV
>C.japonicus JAP2 [GenBank:AB361002]
AILLLLSLPVLAGAITMLLTDRNINTSFFDPAGGGDPILYQHLFWFFGHPEVYILILPGF
GMISHIISQESGKKETFGALGMIYAMLAIGLLGFIVWAHHMFTVGMDVDTRAYFTSATMI
IAVPTGIKIFSWLATLHGTQMTL-NPSLLWSLGFVFLFTVGGLTGVILANSSIDIVLHDT
YYV
>C.kibunensis JAP1 [GenBank:AB646610]
AILLLLSLPVLAGAITMLLTDRNINTSFFDPAGGGDPILYQHLFWFFGHPEVYILILPGF
GMISHIISQESGKKETFGSLGMIYAMLAIGLLGFIVWAHHMFTVGMDVDTRAYFTSATMI

IAVPTGIKIFSWLATLHGTQMTL-NPSLLWSLGFVFLFTVGGLTGVILANSSIDIVLHDT
YYV
>C.kibunensis JAP2 [GenBank:AB646611]
AILLLLSLPVLAGAITMLLTDRNINTSFFDPAGGGDPILYQHLFWFFGHPEVYILILPGF
GMISHIISQESGKKETFGSLGMIYAMLAIGLLGFIVWAHHMFTVGMDVDTRAYFTSATMI
IAVPTGIKIFSWLATLHGTQMTL-NPSLLWSLGFVFLFTVGGLTGVILANSSIDIVLHDT
YYV
>C.maculatus JAP1 [GenBank:AB360986]
AILLLLSLPVLAGAITMLLTDRNINTSFFDPAGGGDPILYQHLFWFFGHPEVYILILPAF
GIISHIIAHESGKKEAFGALAMMYAITTIGLLGFIVWAHHMFTVGLDVDTRAYFSSATMV
IAVPTGIKIFSWMATLYGAHWKY-TPSLLWALGFIFLFTLGGLTGIILANSSIDIVLHDT
YYV
>C.maculatus JAP2 [GenBank:AB360987]
AILLLLSLPVLAGAITMLLTDRNINTSFFDPAGGGDPILYQHLFWFFGHPEVYILILPAF
GIISHIIAHESGKKEAFGALAMMYAITTIGLLGFIVWAHHMFTVGLDVDTRAYFSSATMV
IAVPTGIKIFSWMATLYGAHWKY-TPSLLWALGFIFLFTLGGLTGIILANSSIDIVLHDT
YYV
>C.matsuzawai JAP1 [GenBank:AB364649]
AILLLLSLPVLAGAITMLLTDRNINTSFFDPAGGGDPILYQHLFWFFGHPEVYILILPGF
GMISHIISQESGKKETFGALGMIYAMLAIGLLGFIVWAHHMFTVGMDIDTRAYFTSATMI
IAVPTGIKIFSWLATLHGTQMIF-TPQILWSLGFVFLFTLGGLTGVILANSSIDIILHDT
YYV
>C.matsuzawai JAP2 [GenBank:AB364650]
AILLLLSLPVLAGAITMLLTDRNINTSFFDPAGGGDPILYQHLFWFFGHPEVYILILPGF
GMISHIISQESGKKETFGALGMIYAMLAIGLLGFIVWAHHMFTVGMDIDTRAYFTSATMI
IAVPTGIKIFSWLATLHGTQMIF-TPQILWSLGFVFLFTLGGLTGVILANSSIDIILHDT
YYV
>C.nipponensis JAP [GenBank:AB360999]
AILLLLSLPVLAGAITMLLTDRNFNTSFFDPAGGGDPILYQHLFWFFGHPEVYILILPGF
GIISHIISHESGKKESFGNLGMIYAMTTIGLLGFIVWAHHMFTVGMDVDTRAYFTSATMI
IAVPTGIKIFSWLATIYGSQIMFKNPAILWAIGFVFLFTMGGLTGVILANSSIDVILHDT
YYV
>C.ohmorii JAP1 [GenBank:AB360976]
AILLLLSLPVLAGAITMLLTDRNINTSFFDPAGGGDPILYQHLFWFFGHPEVYILILPGF
GIISHIISSESGKKESFGNLGMIYAMTTIGLLGFIVWAHHMFTVGMDVDTRAYFTSATMI
IAVPTGIKIFSWLASMMGSTNRM-TSSTLWALGFIYLFTLGGMTGIILSNAIIDVMLHDT
YYV
>C.ohmorii JAP2 [GenBank:AB360977]
AILLLLSLPVLAGAITMLLTDRNINTSFFDPAGGGDPILYQHLFWFFGHPEVYILILPGF
GIISHIISSESGKKESFGNLGMIYAMTTIGLLGFIVWAHHMFTVGMDVDTRAYFTSATMI
IAVPTGIKIFSWLASMMGSTNRM-TSSTLWALGFIYLFTLGGMTGIILSNAIIDVMLHDT
YYV
>C.oxystoma JAP1 [GenBank:AB360978]
AILLLLSLPVLAGAITMLLTDRNINTSFFDPAGGGDPILYQHLFWFFGHPEVYILILPGF
GMISHIISQESGKKETFGSLGMIYAMLAIGLLGFIVWAHHMFTVGMDVDTRAYFTSATMI
IAVPTGIKIFSWLATLHGTQMTL-TPSLLWALGFVFLFTVGGLTGVILANSSIDIVLHDT
YYV
>C.oxystoma JAP2 [GenBank:AB360979]
AILLLLSLPVLAGAITMLLTDRNINTSFFDPAGGGDPILYQHLFWFFGHPEVYILILPGF
GMISHIISQESGKKETFGSLGMIYAMLAIGLLGFIVWAHHMFTVGMDVDTRAYFTSATMI
IAVPTGIKIFSWLATLHGTQMTL-TPSLLWALGFVFLFTVGGLTGVILANSSIDIVLHDT
YYV
>C.oxystom JAP3 [GenBank:AB360980]
AILLLLSLPVLAGAITMLLTDRNINTSFFDPAGGGDPILYQHLFWFFGHPEVYILILPGF
GMISHIISQESGKKETFGSLGMIYAMLAIGLLGFIVWAHHMFTVGMDVDTRAYFTSATMI
IAVPTGIKIFSWLATLHGTQMTL-TPSLLWALGFVFLFTVGGLTGVILANSSIDIVLHDT
YYV
>C.oxystoma JAP4 [GenBank:AB360981]
AILLLLSLPVLAGAITMLLTDRNINTSFFDPAGGGDPILYQHLFWFFGHPEVYILILPGF
GMISHIISQESGKKETFGSLGMIYAMLAIGLLGFIVWAHHMFTVGMDVDTRAYFTSATMI
IAVPTGIKIFSWLATLHGTQMTL-TPSLLWALGFVFLFTVGGLTGVILANSSIDIVLHDT
YYV
>C.oxystoma JAP5 [GenBank:AB360982]

```
AILLLLSLPVLAGAITMLLTDRNINTSFFDPAGGGDPILYQHLFWFFGHPEVYILILPGF
GMISHIISQESGKKETFGSLGMIYAMLAIGLLGFIVWAHHMFTVGMDVDTRAYFTSATMI
IAVPTGIKIFSWLATLHGTQMTL-TPSLLWALGFVFLFTVGGLTGVILANSSIDIVLHDT
YYV
>C.oxystoma JAP6 [GenBank:AB360983]
AILLLLSLPVLAGAITMLLTDRNINTSFFDPAGGGDPILYQHLFWFFGHPEVYILILPGF
GMISHIISQESGKKETFGSLGMIYAMLAIGLLGFIVWAHHMFTVGMDVDTRAYFTSATMI
IAVPTGIKIFSWLATLHGTQMTL-TPSLLWALGFVFLFTVGGLTGVILANSSIDIVLHDT
YYV
>C.oxystoma JAP7 [GenBank:AB360984]
AILLLLSLPVLAGAITMLLTDRNINTSFFDPAGGGDPILYQHLFWFFGHPEVYILILPGF
GMISHIISQESGKKETFGSLGMIYAMLAIGLLGFIVWAHHMFTVGMDVDTRAYFTSATMI
IAVPTGIKIFSWLATLHGTQMTL-TPSLLWALGFVFLFTVGGLTGVILANSSIDIVLHDT
YYV
>C.oxystoma JAP8 [GenBank:AB360985]
AILLLLSLPVLAGAITMLLTDRNINTSFFDPAGGGDPILYQHLFWFFGHPEVYILILPGF
GMISHIISQESGKKETFGSLGMIYAMLAIGLLGFIVWAHHMFTVGMDVDTRAYFTSATMI
IAVPTGIKIFSWLATLHGTQMTL-TPSLLWALGFVFLFTVGGLTGVILANSSIDIVLHDT
YYV
>C.peregrinus JAP [GenBank:AB361003]
AILLLLSLPVLAGAITMLLTDRNINTTFFDPTGGGDPILYQHLFWFFGHPEVYILILPGF
GIVSHIMSQESGKKEAFGALGMIYAMTTIGLLGFIVWAHHMFTVGMDVDTRAYFTSATMI
IAIPTGIKIFSWLATILGSPLSSINPPLMWSLGFVFLFTIGGLTGIILANSSIDTVLHDT
YYV
>C.pictimargo JAP1 [GenBank:AB646612]
AILLLLSLPVLAGAITMLLTDRNINTSFFDPAGGGDPILYQHLFWFFGHPEVYILILPGF
GMISHIISQESGKKETFGALGMIYAMLAIGLLGFIVWAHHMFTVGMDVDTRAYFTSATMI
IAVPTGIKIFSWLATLHGTQMTL-NPSLLWSLGFVFLFTVGGLTGVILANSSIDIVLHDT
YYV
>C.pictimargo JAP2 [GenBank:AB646613]
AILLLLSLPVLAGAITMLLTDRNINTSFFDPAGGGDPILYQHLFWFFGHPEVYILILPGF
GMISHIISQESGKKETFGALGMIYAMLAIGLLGFIVWAHHMFTVGMDVDTRAYFTSATMI
IAVPTGIKIFSWLATLHGTQMTL-NPSLLWSLGFVFLFTVGGLTGVILANSSIDIVLHDT
YYV
>C.punctatus JAP1 [GenBank:AB360988]
AILLLLSLPVLAGAITMLLTDRNINTSFFDPAGGGDPILYQHLFWFFGHPEVYILILPGF
GMISHIIAQESGKKETFGALGMIYAMLAIGLLGFIVWAHHMFTVGMDVDTRAYFTSATMI
IAVPTGIKIFSWLATLHGTQMNL-NASLLWSLGFVFLFTVGGLTGVILANSSIDIVLHDT
YYV
>C.punctatus JAP2 [GenBank:AB360989]
AILLLLSLPVLAGAITMLLTDRNINTSFFDPAGGGDPILYQHLFWFFGHPEVYILILPGF
GMISHIIAQESGKKETFGALGMIYAMLAIGLLGFIVWAHHMFTVGMDVDTRAYFTSATMI
IAVPTGIKIFSWLATLHGTQMNL-NASLLWSLGFVFLFTVGGLTGVILANSSIDIVLHDT
YYV
>C.wadai JAP1 [GenBank:AB360996]
AILLLLSLPVLAGAITMLLTDRNINTSFFDPAGGGDPILYQHLFWFFGHPEVYILILPGF
GMISHILSQESGKKETFGALGMIYAMLAIGLLGFIVWAHHMFTVGMDVDTRAYFTSATMI
IAVPTGIKIFSWLATIYGTQLTL-NASLLWSLGFVFLFTMGGLTGVVLANSSIDVVLHDT
YYV
>C.wadai JAP2 [GenBank:AB360997]
AILLLLSLPVLAGAITMLLTDRNINTSFFDPAGGGDPILYQHLFWFFGHPEVYILILPGF
GMISHILSQESGKKETFGALGMIYAMLAIGLLGFIVWAHHMFTVGMDVDTRAYFTSATMI
IAVPTGIKIFSWLATIYGTQLTL-NASLLWSLGFVFLFTMGGLTGVVLANSSIDVVLHDT
YYV
>C.wadai JAP3 [GenBank:AB361005]
AILLLLSLPVLAGAITMLLTDRNINTSFFDPAGGGDPILYQHLFWFFGHPEVYILILPGF
GMISHILSQESGKKETFGALGMIYAMLAIGLLGFIVWAHHMFTVGMDVDTRAYFTSATMI
IAVPTGIKIFSWLATIYGTQLTL-NASLLWSLGFVFLFTMGGLTGVVLANSSIDVVLHDT
YYV
>Anopheles gambiae [GenBank:Nc_002084][1991..2537]
AVLLLLSLPVLAGAITMLLTDRNLNTSFFDPAGGGDPILYQHLFWFFGHPEVYILILPGF
GMISHIITQESGKKETFGNLGMIYAMLAIGLLGFIVWAHHMFTVGMDVDTRAYFTSATMI
IAVPTGIKIFSWLATLHGTQLTY-SPAMLWAFGFVFLFTVGGLTGVVLANSSIDIVLHDT
```

```
YYV
>Anopheles quadriannulatus  [GenBank:DQ792581]
AVLLLLSLPVLAGAITMLLTDRNLNTSFFDPAGGGDPILYQHLFWFFGHPEVYILILPGF
GMISHIITQESGKKETFGNLGMIYAMLAIGLLGFIVWAHHMFTVGMDVDTRAYFTSATMI
IAVPTGIKIFSWLATLHGTQLTY-SPAMLWAFGFVFLFTVGGLTGVVLANSSIDIVLHDT
YYV
>Anopheles dirus_D [GenBank:AJ877572]
AILLLLSLPVLAGAITMLLTDRNLNTSFFDPAGGGDPILYQHLFWFFGHPEVYILILPGF
GMISHIITQESGKKETFGNLGMIYAMLAIGLLGFIVWAHHMFTVGMDVDTRAYFTSATMI
IAVPTGIKIFSWLATLHGTQLTY-SPAMLWAFGFVFLFTVGGLTGVVLANSSIDIVLHDT
YYV
>Anopheles janconnae [GenBank:HQ335348]
AVLLLLSLPVLAGAITMLLTDRNLNTSFFDPAGGGDPILYQHLFWFFGHPEVYILILPGF
GMISHIITQESGKKETFGNLGMIYAMLAIGLLGFIVWAHHMFTVGMDVDTRAYFTSATMI
IAVPTGIKIFSWLATLHGTQLTY-SPAMLWAFGFVFLFTVGGLTGVVLANSSIDIVLHDT
YYV
```
