# Supplementary material for: Population Genetic Structure and Potential Incursion Pathways of the Bluetongue Virus Vector Culicoides brevitarsis (Diptera: Ceratopogonidae) in Australia
Source: PLoS One. 2016 Jan 15;11(1):e0146699. doi: 10.1371/journal.pone.0146699 (PMC4714883; doi:10.1371/journal.pone.0146699)
Supplement: S1 Table — (DOCX) [file pone.0146699.s003.docx]

**S1 Table:** Uncorrected (‘p’)-pairwise distances between Australian *C. brevitarsis* (Cbrev-01 to -09) and [GenBank: AB360994] (Japan).

|  | mtDNA haplotypes | 1 | 2 | 3 | 4 | 5 | 6 | 7 | 8 | 9 | 10 |
| --- | --- | --- | --- | --- | --- | --- | --- | --- | --- | --- | --- |
| 1 | Cbrev-01 | - | 0.001 | 0.001 | 0.005 | 0.001 | 0.004 | 0.006 | 0.002 | 0.004 | 0.070 |
| 2 | Cbrev-02 | 0.002 | - | 0.002 | 0.004 | 0.002 | 0.002 | 0.005 | 0.001 | 0.002 | 0.068 |
| 3 | Cbrev-03 | 0.002 | 0.004 | - | 0.006 | 0.002 | 0.005 | 0.007 | 0.004 | 0.005 | 0.071 |
| 4 | Cbrev-04 | 0.004 | 0.002 | 0.005 | - | 0.006 | 0.006 | 0.001 | 0.005 | 0.006 | 0.070 |
| 5 | Cbrev-05 | 0 | 0.002 | 0.002 | 0.004 | - | 0.005 | 0.007 | 0.004 | 0.005 | 0.068 |
| 6 | Cbrev-06 | 0.004 | 0.002 | 0.005 | 0.004 | 0.004 | - | 0.007 | 0.004 | 0.005 | 0.071 |
| 7 | Cbrev-07 | 0.005 | 0.004 | 0.007 | 0.002 | 0.005 | 0.005 | - | 0.006 | 0.007 | 0.071 |
| 8 | Cbrev-08 | 0.004 | 0.002 | 0.005 | 0.004 | 0.004 | 0.004 | 0.005 | - | 0.004 | 0.070 |
| 9 | Cbrev-09 | 0.004 | 0.002 | 0.005 | 0.004 | 0.004 | 0.004 | 0.005 | 0.004 | - | 0. 068 |
| 10 | AB360994 | 0.086 | 0.084 | 0.089 | 0.082 | 0.086 | 0.087 | 0.084 | 0.086 | 0.082 | - |

Divergence of partial 547bp mtDNA COI sequences between Cbrev-01 and Cbrev09 (nucleotide (nt) 204 to nt 750) were generally low (range 0.0 – 0.7%, lower triangle), and between 8.2% and 8.9% when compared with [GenBank: AB360994] (nt 326 to nt 872). Similar low pairwise distances (0.1% - 0.7%) were observed across 835bp (upper triangle) from Australian *C. brevitarsis* haplotypes, and range from 6.8% to 7.1% when compared with [GenBank: AB360994] (nt 123 to nt 957).
