# Supplementary material for: Population Genetic Structure and Potential Incursion Pathways of the Bluetongue Virus Vector Culicoides brevitarsis (Diptera: Ceratopogonidae) in Australia
Source: PLoS One. 2016 Jan 15;11(1):e0146699. doi: 10.1371/journal.pone.0146699 (PMC4714883; doi:10.1371/journal.pone.0146699)
Supplement: S2 Table — (DOCX) [file pone.0146699.s004.docx]

**S2 Table:** Uncorrected (‘p’)-pairwise distances between *Culicoides marksi*. Pairwise distances between *C. marksi* mtDNA COI haplotypes (547bp) ranged from 0.2% to 2.8% (b).

|  | mtDNA haplotype | 1 | 2 | 3 | 4 | 5 | 6 | 7 | 8 | 9 | 10 | 11 | 12 | 13 | 14 | 15 |
| --- | --- | --- | --- | --- | --- | --- | --- | --- | --- | --- | --- | --- | --- | --- | --- | --- |
| 1 | Cmarksi-01 | - |  |  |  |  |  |  |  |  |  |  |  |  |  |  |
| 2 | Cmarksi-03 | 0.011 | - |  |  |  |  |  |  |  |  |  |  |  |  |  |
| 3 | Cmarksi-04 | 0.020 | 0.020 | - |  |  |  |  |  |  |  |  |  |  |  |  |
| 4 | Cmarksi-05 | 0.017 | 0.006 | 0.026 | - |  |  |  |  |  |  |  |  |  |  |  |
| 5 | Cmarksi-06 | 0.015 | 0.004 | 0.024 | 0.006 | - |  |  |  |  |  |  |  |  |  |  |
| 6 | Cmarksi-07 | 0.022 | 0.022 | 0.002 | 0.028 | 0.026 | - |  |  |  |  |  |  |  |  |  |
| 7 | Cmarksi-08 | 0.020 | 0.020 | 0.004 | 0.026 | 0.024 | 0.006 | - |  |  |  |  |  |  |  |  |
| 8 | Cmarksi-09 | 0.002 | 0.013 | 0.022 | 0.018 | 0.017 | 0.024 | 0.022 | - |  |  |  |  |  |  |  |
| 9 | Cmarksi-10 | 0.026 | 0.022 | 0.017 | 0.028 | 0.026 | 0.018 | 0.017 | 0.028 | - |  |  |  |  |  |  |
| 10 | Cmarksi-11 | 0.026 | 0.026 | 0.017 | 0.028 | 0.026 | 0.018 | 0.017 | 0.028 | 0.022 | - |  |  |  |  |  |
| 11 | Cmarksi-12 | 0.020 | 0.017 | 0.011 | 0.020 | 0.020 | 0.013 | 0.011 | 0.022 | 0.013 | 0.017 | - |  |  |  |  |
| 12 | Cmarksi-13 | 0.009 | 0.009 | 0.018 | 0.015 | 0.013 | 0.020 | 0.018 | 0.011 | 0.024 | 0.024 | 0.018 | - |  |  |  |
| 13 | Cmarksi-14 | 0.002 | 0.013 | 0.022 | 0.018 | 0.017 | 0.020 | 0.022 | 0.004 | 0.028 | 0.028 | 0.022 | 0.011 | - |  |  |
| 14 | Cmarksi-15 | 0.020 | 0.017 | 0.011 | 0.022 | 0.020 | 0.009 | 0.011 | 0.022 | 0.017 | 0.017 | 0.004 | 0.018 | 0.018 | - |  |
| 15 | Cmarksi-16 | 0.011 | 0.011 | 0.020 | 0.013 | 0.011 | 0.022 | 0.020 | 0.013 | 0.022 | 0.022 | 0.020 | 0.002 | 0.013 | 0.020 | - |
