# Supplementary material for: Population Genetic Structure and Potential Incursion Pathways of the Bluetongue Virus Vector Culicoides brevitarsis (Diptera: Ceratopogonidae) in Australia
Source: PLoS One. 2016 Jan 15;11(1):e0146699. doi: 10.1371/journal.pone.0146699 (PMC4714883; doi:10.1371/journal.pone.0146699)
Supplement: S3 Table — Upper-triangle values are standard errors. (DOCX) [file pone.0146699.s005.docx]

**S3 Table:** Uncorrected (‘p’)-pairwise distances between *C. brevitarsis* from Australia, China, Japan, Solomon Islands and Timor-Leste based on 323bp of over-lapping gene region. Upper-triangle values are standard errors.

|  | mtDNA haplotypes | 1 | 2 | 3 | 4 | 5 | 6 | 7 | 8 | 9 | 10 | 11 | 12 | 13 | 14 | 15 | 16 | 17 | 18 | 19 | 20 | 21 |
| --- | --- | --- | --- | --- | --- | --- | --- | --- | --- | --- | --- | --- | --- | --- | --- | --- | --- | --- | --- | --- | --- | --- |
| 1 | Solomon Is [KJ162971] |  | 0.000 | 0.000 | 0.000 | 0.000 | 0.004 | 0.000 | 0.010 | 0.010 | 0.010 | 0.012 | 0.012 | 0.000 | 0.003 | 0.003 | 0.006 | 0.000 | 0.004 | 0.006 | 0.003 | 0.004 |
| 2 | Solomon Is [KJ162970] | 0.000 |  | 0.000 | 0.000 | 0.000 | 0.004 | 0.000 | 0.010 | 0.010 | 0.010 | 0.012 | 0.012 | 0.000 | 0.003 | 0.003 | 0.006 | 0.000 | 0.004 | 0.006 | 0.003 | 0.004 |
| 3 | Solomon Is [KJ162969] | 0.000 | 0.000 |  | 0.000 | 0.000 | 0.004 | 0.000 | 0.010 | 0.010 | 0.010 | 0.012 | 0.012 | 0.000 | 0.003 | 0.003 | 0.006 | 0.000 | 0.004 | 0.006 | 0.003 | 0.004 |
| 4 | Solomon Is [KJ162967] | 0.000 | 0.000 | 0.000 |  | 0.000 | 0.004 | 0.000 | 0.010 | 0.010 | 0.010 | 0.012 | 0.012 | 0.000 | 0.003 | 0.003 | 0.006 | 0.000 | 0.004 | 0.006 | 0.003 | 0.004 |
| 5 | Australia [KJ162974] | 0.000 | 0.000 | 0.000 | 0.000 |  | 0.004 | 0.000 | 0.010 | 0.010 | 0.010 | 0.012 | 0.012 | 0.000 | 0.003 | 0.003 | 0.006 | 0.000 | 0.004 | 0.006 | 0.003 | 0.004 |
| 6 | Timor-Leste [KJ162975] | 0.006 | 0.006 | 0.006 | 0.006 | 0.006 |  | 0.004 | 0.010 | 0.010 | 0.010 | 0.012 | 0.012 | 0.004 | 0.003 | 0.005 | 0.006 | 0.004 | 0.004 | 0.006 | 0.003 | 0.004 |
| 7 | Timor-Leste [KJ162968] | 0.000 | 0.000 | 0.000 | 0.000 | 0.000 | 0.006 |  | 0.010 | 0.010 | 0.010 | 0.012 | 0.012 | 0.000 | 0.003 | 0.003 | 0.006 | 0.000 | 0.004 | 0.006 | 0.003 | 0.004 |
| 8 | China [KJ162966] | 0.034 | 0.034 | 0.034 | 0.034 | 0.034 | 0.034 | 0.034 |  | 0.000 | 0.000 | 0.012 | 0.012 | 0.010 | 0.009 | 0.010 | 0.010 | 0.010 | 0.010 | 0.010 | 0.009 | 0.010 |
| 9 | China [KJ162972] | 0.034 | 0.034 | 0.034 | 0.034 | 0.034 | 0.034 | 0.034 | 0.000 |  | 0.000 | 0.012 | 0.012 | 0.010 | 0.009 | 0.010 | 0.010 | 0.010 | 0.010 | 0.010 | 0.009 | 0.010 |
| 10 | China [KJ162973] | 0.034 | 0.034 | 0.034 | 0.034 | 0.034 | 0.034 | 0.034 | 0.000 | 0.000 |  | 0.012 | 0.012 | 0.010 | 0.009 | 0.010 | 0.010 | 0.010 | 0.010 | 0.010 | 0.009 | 0.010 |
| 11 | Japan [AB360994] | 0.056 | 0.056 | 0.056 | 0.056 | 0.056 | 0.050 | 0.056 | 0.056 | 0.056 | 0.056 |  | 0.000 | 0.012 | 0.012 | 0.012 | 0.012 | 0.012 | 0.012 | 0.012 | 0.012 | 0.012 |
| 12 | Japan [AB360995] | 0.056 | 0.056 | 0.056 | 0.056 | 0.056 | 0.050 | 0.056 | 0.056 | 0.056 | 0.056 | 0.000 |  | 0.012 | 0.012 | 0.012 | 0.012 | 0.012 | 0.012 | 0.012 | 0.012 | 0.012 |
| 13 | Cbrev-01 [KP201844] | 0.000 | 0.000 | 0.000 | 0.000 | 0.000 | 0.006 | 0.000 | 0.034 | 0.034 | 0.034 | 0.056 | 0.056 |  | 0.003 | 0.003 | 0.006 | 0.000 | 0.004 | 0.006 | 0.003 | 0.004 |
| 14 | Cbrev-02 [KP201845] | 0.003 | 0.003 | 0.003 | 0.003 | 0.003 | 0.003 | 0.003 | 0.031 | 0.031 | 0.031 | 0.053 | 0.053 | 0.003 |  | 0.004 | 0.005 | 0.003 | 0.003 | 0.005 | 0.000 | 0.003 |
| 15 | Cbrev-03 [KP201846] | 0.003 | 0.003 | 0.003 | 0.003 | 0.003 | 0.009 | 0.003 | 0.037 | 0.037 | 0.037 | 0.059 | 0.059 | 0.003 | 0.006 |  | 0.007 | 0.003 | 0.005 | 0.007 | 0.004 | 0.005 |
| 16 | Cbrev-04 [KP201847] | 0.012 | 0.012 | 0.012 | 0.012 | 0.012 | 0.012 | 0.012 | 0.034 | 0.034 | 0.034 | 0.056 | 0.056 | 0.012 | 0.009 | 0.015 |  | 0.006 | 0.006 | 0.000 | 0.005 | 0.006 |
| 17 | Cbrev-05 [KP201848] | 0.000 | 0.000 | 0.000 | 0.000 | 0.000 | 0.006 | 0.000 | 0.034 | 0.034 | 0.034 | 0.056 | 0.056 | 0.000 | 0.003 | 0.003 | 0.012 |  | 0.004 | 0.006 | 0.003 | 0.004 |
| 18 | Cbrev-06 [KP201849] | 0.006 | 0.006 | 0.006 | 0.006 | 0.006 | 0.006 | 0.006 | 0.034 | 0.034 | 0.034 | 0.056 | 0.056 | 0.006 | 0.003 | 0.009 | 0.012 | 0.006 |  | 0.006 | 0.003 | 0.004 |
| 19 | Cbrev-07 [KP201850] | 0.012 | 0.012 | 0.012 | 0.012 | 0.012 | 0.012 | 0.012 | 0.034 | 0.034 | 0.034 | 0.056 | 0.056 | 0.012 | 0.009 | 0.016 | 0.000 | 0.012 | 0.012 |  | 0.005 | 0.006 |
| 20 | Cbrev-08 [KP201851] | 0.003 | 0.003 | 0.003 | 0.003 | 0.003 | 0.003 | 0.003 | 0.031 | 0.031 | 0.031 | 0.053 | 0.053 | 0.003 | 0.000 | 0.006 | 0.009 | 0.003 | 0.003 | 0.009 |  | 0.003 |
| 21 | Cbrev-09 [KP201852] | 0.006 | 0.006 | 0.006 | 0.006 | 0.006 | 0.006 | 0.006 | 0.034 | 0.034 | 0.034 | 0.056 | 0.056 | 0.006 | 0.003 | 0.009 | 0.012 | 0.006 | 0.006 | 0.012 | 0.003 |  |

**Note:** Due to the much shorter gene region being compared, Cbrev-01 and -05 shared identical partial mtDNA COI gene sequences across this region. Similarly, Cbrev-04 and -07 are also identical across this partial gene region. Nucleotide distances ranged between 5.6% (Australia-Japan) to 3.4% (Australia-China), 0.6% (Australia-Timor-Leste) and 0% (Australia-Solomon Islands-Timor-Leste).
