# Supplementary material for: Population Genetic Structure and Potential Incursion Pathways of the Bluetongue Virus Vector Culicoides brevitarsis (Diptera: Ceratopogonidae) in Australia
Source: PLoS One. 2016 Jan 15;11(1):e0146699. doi: 10.1371/journal.pone.0146699 (PMC4714883; doi:10.1371/journal.pone.0146699)
Supplement: S5 Table — (DOCX) [file pone.0146699.s007.docx]

**S5 Table:** Uncorrected (“p”)-distances between 25 *Culicoides* species (75 nucleotide sequences) across 547bp of partial mtDNA COI gene using all codon positions, excluding all positions containing gaps and missing data. Nucleotide distances between species range from 11.0% (e.g., *C. henryi* vs. *C. pallidothorax*) to 26.4% (e.g., *C. humeralis* vs. *C. actoni*). The number of base differences per site from averaging over all sequence pairs between groups are shown. Standard error estimate(s) are shown above the diagonal.

|  | *Culicoides* | 1 | 2 | 3 | 4 | 5 | 6 | 7 | 8 | 9 | 10 | 11 | 12 | 13 | 14 | 15 | 16 | 17 | 18 | 19 | 20 | 21 | 22 | 23 | 24 | 25 |
| --- | --- | --- | --- | --- | --- | --- | --- | --- | --- | --- | --- | --- | --- | --- | --- | --- | --- | --- | --- | --- | --- | --- | --- | --- | --- | --- |
| 1 | *brevitarsis* | - | 0.015 | 0.016 | 0.016 | 0.016 | 0.017 | 0.019 | 0.016 | 0.017 | 0.015 | 0.017 | 0.015 | 0.020 | 0.017 | 0.016 | 0.016 | 0.017 | 0.016 | 0.016 | 0.017 | 0.015 | 0.019 | 0.016 | 0.017 | 0.016 |
| 2 | *marksi* | 0.158 | - | 0.014 | 0.014 | 0.016 | 0.015 | 0.020 | 0.015 | 0.015 | 0.017 | 0.017 | 0.015 | 0.020 | 0.018 | 0.015 | 0.015 | 0.018 | 0.015 | 0.016 | 0.019 | 0.015 | 0.017 | 0.014 | 0.017 | 0.017 |
| 3 | *pallidothorax* | 0.160 | 0.124 | - | 0.014 | 0.016 | 0.016 | 0.021 | 0.016 | 0.016 | 0.018 | 0.018 | 0.016 | 0.020 | 0.018 | 0.016 | 0.017 | 0.019 | 0.015 | 0.017 | 0.019 | 0.015 | 0.017 | 0.016 | 0.016 | 0.016 |
| 4 | *henryi* | 0.172 | 0.117 | 0.110 | - | 0.016 | 0.014 | 0.020 | 0.015 | 0.016 | 0.018 | 0.018 | 0.014 | 0.019 | 0.019 | 0.015 | 0.015 | 0.018 | 0.015 | 0.016 | 0.018 | 0.014 | 0.018 | 0.016 | 0.016 | 0.017 |
| 5 | *bundyensis* | 0.156 | 0.151 | 0.132 | 0.133 | - | 0.017 | 0.021 | 0.016 | 0.015 | 0.018 | 0.019 | 0.015 | 0.020 | 0.018 | 0.016 | 0.017 | 0.019 | 0.016 | 0.016 | 0.019 | 0.016 | 0.019 | 0.017 | 0.017 | 0.018 |
| 6 | *bunrooensis* | 0.162 | 0.133 | 0.132 | 0.140 | 0.148 | - | 0.021 | 0.016 | 0.016 | 0.019 | 0.017 | 0.015 | 0.020 | 0.018 | 0.014 | 0.016 | 0.019 | 0.016 | 0.016 | 0.018 | 0.013 | 0.018 | 0.016 | 0.016 | 0.017 |
| 7 | *actoni* | 0.186 | 0.223 | 0.240 | 0.243 | 0.240 | 0.233 | - | 0.020 | 0.021 | 0.022 | 0.021 | 0.019 | 0.020 | 0.021 | 0.020 | 0.020 | 0.020 | 0.019 | 0.018 | 0.019 | 0.019 | 0.019 | 0.020 | 0.021 | 0.020 |
| 8 | *arakawae* | 0.154 | 0.142 | 0.125 | 0.133 | 0.134 | 0.148 | 0.236 | - | 0.015 | 0.018 | 0.018 | 0.015 | 0.019 | 0.019 | 0.015 | 0.016 | 0.018 | 0.016 | 0.017 | 0.019 | 0.015 | 0.018 | 0.016 | 0.017 | 0.017 |
| 9 | *verbosus* | 0.169 | 0.128 | 0.141 | 0.139 | 0.132 | 0.145 | 0.233 | 0.119 | - | 0.018 | 0.016 | 0.014 | 0.019 | 0.020 | 0.015 | 0.015 | 0.020 | 0.016 | 0.018 | 0.019 | 0.016 | 0.020 | 0.015 | 0.016 | 0.017 |
| 10 | *brevipalpis* | 0.146 | 0.204 | 0.198 | 0.209 | 0.183 | 0.198 | 0.242 | 0.181 | 0.207 | - | 0.018 | 0.017 | 0.021 | 0.018 | 0.017 | 0.018 | 0.020 | 0.017 | 0.017 | 0.018 | 0.017 | 0.019 | 0.018 | 0.019 | 0.018 |
| 11 | *cylindratus* | 0.191 | 0.190 | 0.210 | 0.196 | 0.215 | 0.169 | 0.244 | 0.198 | 0.188 | 0.209 | - | 0.016 | 0.020 | 0.018 | 0.017 | 0.017 | 0.019 | 0.017 | 0.017 | 0.018 | 0.016 | 0.019 | 0.017 | 0.017 | 0.017 |
| 12 | *dubius* | 0.120 | 0.132 | 0.132 | 0.121 | 0.132 | 0.126 | 0.209 | 0.122 | 0.117 | 0.178 | 0.166 | - | 0.020 | 0.019 | 0.015 | 0.015 | 0.018 | 0.015 | 0.016 | 0.018 | 0.014 | 0.018 | 0.015 | 0.015 | 0.016 |
| 13 | *humeralis* | 0.207 | 0.188 | 0.176 | 0.195 | 0.211 | 0.189 | 0.264 | 0.167 | 0.185 | 0.229 | 0.218 | 0.185 | - | 0.019 | 0.017 | 0.018 | 0.020 | 0.016 | 0.018 | 0.019 | 0.017 | 0.019 | 0.017 | 0.018 | 0.018 |
| 14 | *jacobsoni* | 0.176 | 0.193 | 0.185 | 0.218 | 0.198 | 0.178 | 0.254 | 0.185 | 0.216 | 0.187 | 0.216 | 0.200 | 0.238 | - | 0.018 | 0.019 | 0.019 | 0.018 | 0.018 | 0.020 | 0.018 | 0.018 | 0.018 | 0.019 | 0.017 |
| 6 | *japonicus* | 0.169 | 0.136 | 0.139 | 0.140 | 0.161 | 0.130 | 0.236 | 0.117 | 0.148 | 0.192 | 0.186 | 0.121 | 0.174 | 0.178 | - | 0.017 | 0.018 | 0.016 | 0.017 | 0.018 | 0.015 | 0.019 | 0.015 | 0.016 | 0.016 |
| 16 | *kibunensis* | 0.166 | 0.139 | 0.153 | 0.140 | 0.154 | 0.154 | 0.247 | 0.145 | 0.121 | 0.214 | 0.200 | 0.130 | 0.205 | 0.222 | 0.159 | - | 0.018 | 0.016 | 0.017 | 0.019 | 0.015 | 0.020 | 0.015 | 0.017 | 0.016 |
| 17 | *maculatus* | 0.175 | 0.197 | 0.219 | 0.215 | 0.204 | 0.210 | 0.203 | 0.191 | 0.209 | 0.228 | 0.222 | 0.200 | 0.217 | 0.228 | 0.199 | 0.198 | - | 0.018 | 0.019 | 0.019 | 0.018 | 0.019 | 0.018 | 0.018 | 0.019 |
| 18 | *matsuzawai* | 0.168 | 0.144 | 0.139 | 0.155 | 0.162 | 0.159 | 0.227 | 0.143 | 0.151 | 0.205 | 0.192 | 0.144 | 0.169 | 0.203 | 0.162 | 0.149 | 0.210 | - | 0.016 | 0.018 | 0.016 | 0.019 | 0.016 | 0.016 | 0.016 |
| 19 | *nipponensis* | 0.160 | 0.161 | 0.165 | 0.167 | 0.163 | 0.156 | 0.214 | 0.166 | 0.174 | 0.156 | 0.189 | 0.152 | 0.209 | 0.196 | 0.178 | 0.165 | 0.211 | 0.166 | - | 0.019 | 0.016 | 0.018 | 0.017 | 0.017 | 0.017 |
| 20 | *ohmorii* | 0.193 | 0.225 | 0.205 | 0.213 | 0.222 | 0.192 | 0.238 | 0.207 | 0.220 | 0.207 | 0.217 | 0.207 | 0.240 | 0.228 | 0.211 | 0.224 | 0.248 | 0.200 | 0.196 | - | 0.018 | 0.019 | 0.018 | 0.020 | 0.019 |
| 21 | *oxystoma* | 0.154 | 0.112 | 0.127 | 0.118 | 0.137 | 0.112 | 0.208 | 0.133 | 0.129 | 0.183 | 0.180 | 0.111 | 0.192 | 0.184 | 0.120 | 0.124 | 0.197 | 0.150 | 0.143 | 0.203 | - | 0.018 | 0.016 | 0.015 | 0.017 |
| 22 | *peregrinus* | 0.184 | 0.175 | 0.178 | 0.185 | 0.203 | 0.198 | 0.227 | 0.181 | 0.207 | 0.205 | 0.217 | 0.167 | 0.225 | 0.196 | 0.183 | 0.211 | 0.215 | 0.211 | 0.172 | 0.220 | 0.189 | - | 0.018 | 0.020 | 0.019 |
| 23 | *pictimargo* | 0.165 | 0.131 | 0.130 | 0.140 | 0.140 | 0.152 | 0.243 | 0.140 | 0.135 | 0.192 | 0.187 | 0.124 | 0.186 | 0.205 | 0.137 | 0.145 | 0.217 | 0.167 | 0.162 | 0.213 | 0.139 | 0.182 | - | 0.016 | 0.016 |
| 24 | *punctatus* | 0.173 | 0.162 | 0.155 | 0.144 | 0.162 | 0.143 | 0.240 | 0.168 | 0.144 | 0.224 | 0.186 | 0.127 | 0.206 | 0.197 | 0.150 | 0.155 | 0.207 | 0.160 | 0.176 | 0.237 | 0.136 | 0.220 | 0.163 | - | 0.017 |
| 25 | *wadai* | 0.140 | 0.162 | 0.159 | 0.180 | 0.170 | 0.154 | 0.229 | 0.157 | 0.156 | 0.198 | 0.198 | 0.145 | 0.200 | 0.181 | 0.154 | 0.141 | 0.205 | 0.165 | 0.167 | 0.211 | 0.163 | 0.194 | 0.153 | 0.172 | - |

**Note:** average distance (d ± s.e.) for samples with multiple haplotypes (N_Hap_): *C. brevitarsis* (N_Hap_ = 10, 0.018 ± 0.003), *C. marksi* (N_Hap_ = 15, 0.018 ± 0.004); *C. henryi* (N_Hap_ = 2, 0.022 ± 0.006); *C. arakawae* (N_Hap_ = 5, 0.004 ± 0.002); *C. cylindratus* (N_Hap_ = 2, 0.002 ± 0.002); *C. humeralis* (N_Hap_ = 3, 0.0 ± 0.0); *C. jacobsoni* (N _Hap_ = 2, 0.004 ± 0.003); *C. japonicus* (N_Hap =_ 2, 0.0 ± 0.0); *C. kibunensis* (N_Hap_ = 2, 0.004 ± 0.003); *C. maculatus* (N_Hap_ = 2, 0.011 ± 0.005); *C. matsuzawai* (N_Hap_ = 2, 0.055 ± 0.010); *C. ohmorii* (N_Hap_ = 2, 0.0 ± 0.0); *C. oxystoma* (N_Hap_ = 10, 0.008 ± 0.003); *C. pictimargo* (N_Hap_ = 2, 0.024 ± 0.007); *C. punctatus* (N_Hap_ = 2, 0.007 ± 0.003); *C. wadai* (N_Hap_ = 3, 0.0 ± 0.0).
